# Supplementary material for: The varying impacts of COVID-19 and its related measures in the UK: A year in review
Source: PLoS One. 2021 Sep 29;16(9):e0257286. doi: 10.1371/journal.pone.0257286 (PMC8480884; doi:10.1371/journal.pone.0257286)
Supplement: S1 Table — (DOCX) [file pone.0257286.s001.docx]

**S1 Table. Samples and sample selection.**

| COVID study survey wave number | Period | Full sample (20-65 years old) | Samples with no missing value in predictors | People worked in January/February 2020 and followed in each wave | *Worker sample*: no missing value in labor income | *Worker sample*: no missing value in weekly working hours | No missing value in subjective wellbeing score | No missing value in weekly housework hours | No missing value in weekly childcare hours (asked *parents*) |
| --- | --- | --- | --- | --- | --- | --- | --- | --- | --- |
| 1 | Apr 20 | 13,119 | 12,943 | 10,106 | 9,257 | 10,018 | 11,512 | 11,687 | 5,065 |
| 2 | May 20 | 10,649 | 10,532 | 8,410 | 7,695 | 8,360 | 10,253 | 10,117 | 4,129 |
| 3 | Jun 20 | 10,048 | 9,943 | 7,883 | 7,114 | 7,833 | 9,618 | 9,505 | 3,742 |
| 4 | Jul 20 | 9,739 | 9,630 | 7,608 | 6,773 | 7,565 | 9,347 | - | - |
| 5 | Sep 20 | 9,060 | 8,966 | 7,050 | 6,266 | 6,998 | 8,591 | 8,515 | 3,163 |
| 6 | Nov 20 | 8,392 | 8,317 | 6,541 | 5,728 | 6,490 | 8,033 | - | - |
| 7 | Jan 2021 | 8,325 | 8,237 | 6,459 | 5,601 | 6,390 | 7,902 | 7,748 | 2,796 |
| 8 | Mar 2021 | 8,975 | 8,880 | 6,963 | 6,078 | 6,916 | 8,560 | - | - |
| Total |  | 78,307 | 77,448 | 61,020 | 54,512 | 60,570 | 73,816 | 47,572 | 18,895 |
| Number of person-years with repeated observations | | | | | 52,710 | 58,306 | 70,363 | 48,364 | 14,895 |
