# Supplementary material for: The varying impacts of COVID-19 and its related measures in the UK: A year in review
Source: PLoS One. 2021 Sep 29;16(9):e0257286. doi: 10.1371/journal.pone.0257286 (PMC8480884; doi:10.1371/journal.pone.0257286)
Supplement: S2 Table — (DOCX) [file pone.0257286.s002.docx]

**S2 Table. Baseline model: Changes in the five indicators across waves.**

|  |  |  | Ln net earnings | Weekly working hours | Subjective wellbeing | Weekly housework hours | Weekly childcare hours |
| --- | --- | --- | --- | --- | --- | --- | --- |
| UK  lockdown stage |  |  | Reference period:  Jan/Feb 2020 | | Reference period:  2018/19 | | Reference period: Apr 2020 |
| 1^st^ lockdown | Apr-20 | | -0.916^***^ | -12.725^***^ | 1.360^***^ | 3.254^***^ |  |
|  |  |  | (0.040) | (0.290) | (0.102) | (0.149) |  |
| 1^st^ lockdown | May-20 | | -0.555^***^ | -10.877^***^ | 1.347^***^ | 3.156^***^ | -0.712 |
|  |  |  | (0.036) | (0.279) | (0.102) | (0.156) | (0.540) |
| Schools reopened | Jun-20 | | -0.566^***^ | -8.696^***^ | 1.346^***^ | 2.165^***^ | -3.534^***^ |
|  |  |  | (0.034) | (0.281) | (0.098) | (0.154) | (0.526) |
| Easing | Jul-20 | | -0.597^***^ | -7.772^***^ | 0.762^***^ |  |  |
|  |  |  | (0.035) | (0.269) | (0.099) |  |  |
| Easing | Sep-20 | | -0.660^***^ | -4.576^***^ | 0.734^***^ | 1.354^***^ | -4.389^***^ |
|  |  |  | (0.036) | (0.242) | (0.100) | (0.146) | (0.633) |
| 2^nd^ lockdown | Nov-20 | | -0.733^***^ | -5.772^***^ | 1.681^***^ |  |  |
|  |  |  | (0.041) | (0.300) | (0.102) |  |  |
| 3^rd^ lockdown | Jan-21 | | -0.823^***^ | -6.768^***^ | 1.636^***^ | 1.645^***^ | -3.958^***^ |
|  |  |  | (0.046) | (0.310) | (0.109) | (0.146) | (0.628) |
| 3^rd^ lockdown, but | Mar-21 | | -0.736^***^ | -6.022^***^ | 1.154^***^ |  |  |
| schools reopened |  |  | (0.042) | (0.310) | (0.106) |  |  |
|  | Living with a partner | | 0.032 | 1.145^*^ | 0.249 | 0.144 | 0.508 |
|  |  |  | (0.066) | (0.581) | (0.177) | (0.308) | (1.577) |
|  | Child<=15yrs | | -0.137 | -1.876^*^ | 0.079 | 1.096^*^ |  |
|  |  |  | (0.150) | (0.913) | (0.210) | (0.450) |  |
|  | COVID test result (ref: No test) | |  |  |  |  |  |
|  |  | Positive | -0.245 | -4.642^***^ | 0.907^**^ | -1.409^**^ | 0.765 |
|  |  |  | (0.250) | (1.368) | (0.288) | (0.510) | (1.216) |
|  |  | Negative | 0.159^***^ | 0.960^**^ | 0.017 | -0.428^**^ | -0.411 |
|  |  |  | (0.039) | (0.296) | (0.107) | (0.159) | (0.759) |
|  |  | Pending | -0.138 | -1.315 | 0.277 | 0.746 | 5.712 |
|  |  |  | (0.218) | (1.174) | (0.287) | (0.638) | (3.643) |
|  | Constant | | 7.214^***^ | 34.571^***^ | 11.542^***^ | 8.599^***^ | 16.477^***^ |
|  |  |  | (0.077) | (0.558) | (0.141) | (0.249) | (1.284) |
|  | R2 | | 0.012 | 0.040 | 0.003 | 0.027 | 0.006 |
|  | Within R2 | | 0.038 | 0.120 | 0.021 | 0.046 | 0.020 |
|  | Between R2 | | 0.012 | 0.004 | 0.001 | 0.031 | 0.001 |
|  | Rho | | 0.618 | 0.591 | 0.613 | 0.627 | 0.658 |
|  | Number of individuals | | 8621 | 9047 | 11043 | 10946 | 4542 |
|  | Number of person-years | | 52710 | 58306 | 70363 | 48364 | 14895 |

Data: UKHLS & Understanding Society Covid survey waves 1-8.

Note: * p<0.05 ** p<0.01 *** p<0.001
