# Supplementary material for: The varying impacts of COVID-19 and its related measures in the UK: A year in review
Source: PLoS One. 2021 Sep 29;16(9):e0257286. doi: 10.1371/journal.pone.0257286 (PMC8480884; doi:10.1371/journal.pone.0257286)
Supplement: S3 Table — (DOCX) [file pone.0257286.s003.docx]

**S3 Table. Gender and period interaction model results.**

|  |  | Ln net earnings | Ln net earnings - non-key worker | Weekly working hours | Weekly working hours - non-key worker | Subjective wellbeing | Weekly housework hours | Weekly childcare hours |
| --- | --- | --- | --- | --- | --- | --- | --- | --- |
|  |  | Reference period:  Jan/Feb 2020 | | | | Reference period:  2018/19 | | Reference period: Apr 2020 |
| Apr-20 |  | -1.020^***^ | -1.487^***^ | -14.424^***^ | -19.265^***^ | 0.857^***^ | 3.484^***^ |  |
|  |  | (0.066) | (0.096) | (0.488) | (0.620) | (0.158) | (0.219) |  |
| May-20 |  | -0.579^***^ | -0.810^***^ | -12.041^***^ | -15.229^***^ | 1.171^***^ | 3.041^***^ | -0.424 |
|  |  | (0.056) | (0.081) | (0.461) | (0.610) | (0.158) | (0.208) | (0.766) |
| Jun-20 |  | -0.580^***^ | -0.745^***^ | -9.342^***^ | -11.744^***^ | 1.262^***^ | 2.172^***^ | -3.498^***^ |
|  |  | (0.056) | (0.079) | (0.460) | (0.610) | (0.150) | (0.231) | (0.640) |
| Jul-20 |  | -0.633^***^ | -0.799^***^ | -7.971^***^ | -9.503^***^ | 0.863^***^ |  |  |
|  |  | (0.055) | (0.079) | (0.426) | (0.549) | (0.158) |  |  |
| Sep-20 |  | -0.665^***^ | -0.805^***^ | -5.024^***^ | -6.131^***^ | 0.672^***^ | 1.404^***^ | -3.490^***^ |
|  |  | (0.056) | (0.077) | (0.406) | (0.528) | (0.158) | (0.195) | (0.789) |
| Nov-20 |  | -0.756^***^ | -0.912^***^ | -6.530^***^ | -8.183^***^ | 1.476^***^ |  |  |
|  |  | (0.066) | (0.092) | (0.500) | (0.699) | (0.157) |  |  |
| Jan-21 |  | -0.806^***^ | -1.017^***^ | -7.351^***^ | -9.209^***^ | 1.417^***^ | 1.669^***^ | -3.185^***^ |
|  |  | (0.075) | (0.104) | (0.527) | (0.720) | (0.172) | (0.193) | (0.773) |
| Mar-21 |  | -0.682^***^ | -0.798^***^ | -6.608^***^ | -7.849^***^ | 0.990^***^ |  |  |
|  |  | (0.063) | (0.084) | (0.496) | (0.679) | (0.169) |  |  |
| Women # Apr-20 | | 0.202^*^ | 0.038 | 3.315^***^ | 3.631^***^ | 0.929^***^ | -0.425 |  |
|  |  | (0.080) | (0.124) | (0.576) | (0.742) | (0.194) | (0.290) |  |
| Women # May-20 | | 0.050 | -0.081 | 2.253^***^ | 1.769^*^ | 0.330 | 0.205 | -0.502 |
|  |  | (0.068) | (0.105) | (0.556) | (0.747) | (0.196) | (0.295) | (1.038) |
| Women # Jun-20 | | 0.030 | -0.094 | 1.284^*^ | 0.588 | 0.163 | -0.016 | -0.077 |
|  |  | (0.068) | (0.098) | (0.544) | (0.722) | (0.189) | (0.293) | (0.960) |
| Women # Jul-20 | | 0.071 | -0.088 | 0.434 | 0.366 | -0.179 |  |  |
|  |  | (0.071) | (0.107) | (0.522) | (0.685) | (0.191) |  |  |
| Women # Sep-20 | | 0.013 | -0.145 | 0.917 | 0.487 | 0.123 | -0.093 | -1.553 |
|  |  | (0.072) | (0.106) | (0.520) | (0.709) | (0.195) | (0.281) | (1.060) |
| Women # Nov-20 | | 0.045 | -0.089 | 1.488^*^ | 1.016 | 0.382 |  |  |
|  |  | (0.082) | (0.122) | (0.587) | (0.819) | (0.200) |  |  |
| Women # Jan-21 | | -0.028 | -0.075 | 1.164 | 0.802 | 0.405 | -0.046 | -1.334 |
|  |  | (0.096) | (0.141) | (0.627) | (0.873) | (0.216) | (0.281) | (1.008) |
| Women # Mar-21 | | -0.102 | -0.216 | 1.161^*^ | 0.374 | 0.306 |  |  |
|  |  | (0.080) | (0.116) | (0.576) | (0.815) | (0.205) |  |  |
| Living with a partner | | 0.034 | 0.043 | 1.148^*^ | 1.361 | 0.250 | 0.147 | 0.622 |
|  |  | (0.066) | (0.103) | (0.580) | (0.737) | (0.179) | (0.309) | (1.566) |
| Child<=15yrs | | -0.136 | -0.253 | -1.857^*^ | -2.045 | 0.092 | 1.094^*^ |  |
|  |  | (0.149) | (0.249) | (0.905) | (1.229) | (0.212) | (0.447) |  |
| COVID test result (ref: No test) | |  |  |  |  |  |  |  |
|  | Positive | -0.241 | -0.447 | -4.625^***^ | -5.060^*^ | 0.913^**^ | -1.416^**^ | 0.816 |
|  |  | (0.250) | (0.476) | (1.363) | (2.467) | (0.288) | (0.511) | (1.224) |
|  | Negative | 0.164^***^ | 0.202^**^ | 0.973^***^ | 1.000^*^ | 0.017 | -0.433^**^ | -0.375 |
|  |  | (0.039) | (0.063) | (0.294) | (0.456) | (0.105) | (0.159) | (0.758) |
|  | Pending | -0.137 | -0.089 | -1.325 | -1.414 | 0.274 | 0.731 | 5.698 |
|  |  | (0.219) | (0.295) | (1.168) | (1.644) | (0.289) | (0.638) | (3.625) |
|  |  |  |  |  |  |  |  |  |
| Constant | | 7.211^***^ | 7.211^***^ | 34.532^***^ | 33.958^***^ | 11.534^***^ | 8.600^***^ | 16.386^***^ |
|  |  | (0.077) | (0.118) | (0.555) | (0.679) | (0.142) | (0.248) | (1.278) |
| R2 | | 0.011 | 0.023 | 0.028 | 0.058 | 0.007 | 0.025 | 0.003 |
| Within R2 | | 0.038 | 0.064 | 0.122 | 0.195 | 0.023 | 0.046 | 0.021 |
| Between R2 | | 0.009 | 0.038 | 0.003 | 0.001 | 0.001 | 0.026 | 0.000 |
| Rho |  | 0.618 | 0.613 | 0.597 | 0.594 | 0.612 | 0.627 | 0.659 |
| Number of individuals | | 8621 | 5339 | 9047 | 5631 | 11043 | 10946 | 4542 |
| Number of person-years | | 52710 | 30885 | 58306 | 34367 | 70363 | 48364 | 14895 |

Data: UKHLS & Understanding Society Covid survey waves 1-8.

Note: * p<0.05 ** p<0.01 *** p<0.001
