# Supplementary material for: The varying impacts of COVID-19 and its related measures in the UK: A year in review
Source: PLoS One. 2021 Sep 29;16(9):e0257286. doi: 10.1371/journal.pone.0257286 (PMC8480884; doi:10.1371/journal.pone.0257286)
Supplement: S4 Table — (DOCX) [file pone.0257286.s004.docx]

**S4 Table. Ethnicity and period interaction models.**

|  |  | Ln net earnings | Ln net earnings - non-key worker | Weekly working hours | Weekly working hours - non-key worker | Subjective wellbeing | Weekly housework hours | Weekly childcare hours |
| --- | --- | --- | --- | --- | --- | --- | --- | --- |
|  |  | Reference period:  Jan/Feb 2020 | | | | Reference period:  2018/19 | | Reference period: Apr 2020 |
| Apr-20 |  | -0.870^***^ | -1.405^***^ | -12.825^***^ | -17.725^***^ | 1.310^***^ | 3.177^***^ |  |
|  |  | (0.042) | (0.066) | (0.307) | (0.422) | (0.100) | (0.150) |  |
| May-20 |  | -0.522^***^ | -0.802^***^ | -10.928^***^ | -14.475^***^ | 1.305^***^ | 2.999^***^ | -0.603 |
|  |  | (0.037) | (0.058) | (0.293) | (0.399) | (0.103) | (0.147) | (0.588) |
| Jun-20 |  | -0.541^***^ | -0.754^***^ | -8.693^***^ | -11.457^***^ | 1.284^***^ | 2.093^***^ | -3.548^***^ |
|  |  | (0.035) | (0.053) | (0.295) | (0.398) | (0.100) | (0.156) | (0.561) |
| Jul-20 |  | -0.566^***^ | -0.804^***^ | -7.861^***^ | -9.439^***^ | 0.723^***^ |  |  |
|  |  | (0.036) | (0.056) | (0.278) | (0.379) | (0.101) |  |  |
| Sep-20 |  | -0.622^***^ | -0.825^***^ | -4.580^***^ | -5.905^***^ | 0.727^***^ | 1.231^***^ | -4.377^***^ |
|  |  | (0.036) | (0.054) | (0.246) | (0.338) | (0.104) | (0.144) | (0.674) |
| Nov-20 |  | -0.706^***^ | -0.918^***^ | -5.719^***^ | -7.695^***^ | 1.673^***^ |  |  |
|  |  | (0.042) | (0.063) | (0.311) | (0.445) | (0.104) |  |  |
| Jan-21 |  | -0.810^***^ | -1.044^***^ | -6.826^***^ | -9.021^***^ | 1.643^***^ | 1.596^***^ | -3.789^***^ |
|  |  | (0.048) | (0.068) | (0.320) | (0.451) | (0.112) | (0.152) | (0.647) |
| Mar-21 |  | -0.723^***^ | -0.885^***^ | -6.276^***^ | -8.033^***^ | 1.140^***^ |  |  |
|  |  | (0.043) | (0.062) | (0.319) | (0.442) | (0.110) |  |  |
| BAME # Apr-20 | | -0.598^***^ | -0.811^***^ | 1.244 | 1.696 | 0.595 | 0.866 |  |
|  |  | (0.151) | (0.228) | (0.952) | (1.229) | (0.517) | (0.565) |  |
| BAME # May-20 | | -0.407^**^ | -0.535^*^ | 0.550 | 0.725 | 0.495 | 1.933^*^ | -1.225 |
|  |  | (0.145) | (0.208) | (0.980) | (1.171) | (0.395) | (0.839) | (1.151) |
| BAME # Jun-20 | | -0.297^*^ | -0.426^*^ | -0.257 | -0.924 | 0.788^*^ | 0.810 | 0.164 |
|  |  | (0.143) | (0.207) | (0.937) | (1.246) | (0.373) | (0.740) | (1.204) |
| BAME # Jul-20 | | -0.405^**^ | -0.418^*^ | 1.112 | 0.845 | 0.460 |  |  |
|  |  | (0.155) | (0.210) | (1.099) | (1.572) | (0.358) |  |  |
| BAME # Sep-20 | | -0.510^**^ | -0.630^*^ | -0.149 | -0.749 | 0.033 | 1.558^*^ | -0.133 |
|  |  | (0.170) | (0.263) | (1.129) | (1.667) | (0.405) | (0.775) | (1.361) |
| BAME # Nov-20 | | -0.341^*^ | -0.414 | -1.015 | -0.622 | 0.031 |  |  |
|  |  | (0.161) | (0.238) | (1.026) | (1.304) | (0.505) |  |  |
| BAME # Jan-21 | | -0.114 | 0.044 | 0.670 | 2.312 | -0.202 | 0.469 | -1.807 |
|  |  | (0.218) | (0.343) | (1.317) | (1.907) | (0.454) | (0.585) | (1.881) |
| BAME # Mar-21 | | -0.138 | -0.134 | 3.636^**^ | 4.669^*^ | 0.108 |  |  |
|  |  | (0.209) | (0.330) | (1.325) | (2.036) | (0.424) |  |  |
| Living with a partner | | 0.034 | 0.039 | 1.147 | 1.339 | 0.256 | 0.175 | 0.507 |
|  |  | (0.067) | (0.103) | (0.585) | (0.750) | (0.177) | (0.308) | (1.581) |
| Child<=15yrs | | -0.140 | -0.259 | -1.881^*^ | -2.090 | 0.083 | 1.088^*^ |  |
|  |  | (0.150) | (0.251) | (0.911) | (1.242) | (0.211) | (0.452) |  |
| COVID test result (ref: No test) | |  |  |  |  |  |  |  |
|  | Positive | -0.248 | -0.454 | -4.693^***^ | -5.181^*^ | 0.916^**^ | -1.392^**^ | 0.757 |
|  |  | (0.251) | (0.479) | (1.370) | (2.486) | (0.288) | (0.508) | (1.217) |
|  | Negative | 0.161^***^ | 0.198^**^ | 0.983^***^ | 0.990^*^ | 0.016 | -0.436^**^ | -0.426 |
|  |  | (0.039) | (0.062) | (0.298) | (0.458) | (0.106) | (0.158) | (0.758) |
|  | Pending | -0.133 | -0.098 | -1.320 | -1.506 | 0.273 | 0.747 | 5.679 |
|  |  | (0.219) | (0.295) | (1.173) | (1.645) | (0.286) | (0.639) | (3.647) |
|  |  |  |  |  |  |  |  |  |
| Constant | | 7.211^***^ | 7.211^***^ | 7.210^***^ | 34.579^***^ | 34.030^***^ | 11.540^***^ | 8.590^***^ |
|  |  | (0.077) | (0.119) | (0.560) | (0.689) | (0.141) | (0.250) | (1.287) |
| R2 | | 0.016 | 0.025 | 0.040 | 0.067 | 0.004 | 0.030 | 0.007 |
| Within R2 | | 0.039 | 0.065 | 0.121 | 0.194 | 0.021 | 0.046 | 0.020 |
| Between R2 | | 0.022 | 0.044 | 0.004 | 0.010 | 0.000 | 0.037 | 0.002 |
| Rho | 0.616 | 0.611 | 0.591 | 0.589 | 0.613 | 0.626 | 0.658 | 0.659 |
| Number of individuals | | 8621 | 5339 | 9047 | 5631 | 11043 | 10946 | 4542 |
| Number of person-years | | 52710 | 30885 | 58306 | 34367 | 70363 | 48364 | 14895 |

Data: UKHLS & Understanding Society Covid survey waves 1-8.

Note: * p<0.05 ** p<0.01 *** p<0.001
