# Supplementary material for: The varying impacts of COVID-19 and its related measures in the UK: A year in review
Source: PLoS One. 2021 Sep 29;16(9):e0257286. doi: 10.1371/journal.pone.0257286 (PMC8480884; doi:10.1371/journal.pone.0257286)
Supplement: S5 Table — (DOCX) [file pone.0257286.s005.docx]

**S5 Table. Education and period interaction model results.**

|  |  | Ln net earnings | Ln net earnings - non-key worker | Weekly working hours | Weekly working hours - non-key worker | Subjective wellbeing | Weekly housework hours | Weekly childcare hours |
| --- | --- | --- | --- | --- | --- | --- | --- | --- |
|  |  | Reference period:  Jan/Feb 2020 | | | | Reference period:  2018/19 | | Reference period: Apr 2020 |
| Apr-20 |  | -1.030^***^ | -1.721^***^ | -14.617^***^ | -21.235^***^ | 1.329^***^ | 3.310^***^ |  |
|  |  | (0.056) | (0.091) | (0.406) | (0.552) | (0.131) | (0.208) |  |
| May-20 |  | -0.621^***^ | -0.987^***^ | -12.182^***^ | -16.930^***^ | 1.277^***^ | 3.209^***^ | -0.303 |
|  |  | (0.049) | (0.079) | (0.386) | (0.526) | (0.132) | (0.214) | (0.801) |
| Jun-20 |  | -0.649^***^ | -0.928^***^ | -9.458^***^ | -12.985^***^ | 1.302^***^ | 2.233^***^ | -3.418^***^ |
|  |  | (0.047) | (0.072) | (0.389) | (0.531) | (0.123) | (0.199) | (0.750) |
| Jul-20 |  | -0.659^***^ | -0.939^***^ | -7.605^***^ | -9.891^***^ | 0.763^***^ |  |  |
|  |  | (0.049) | (0.077) | (0.364) | (0.508) | (0.130) |  |  |
| Sep-20 |  | -0.739^***^ | -1.001^***^ | -4.654^***^ | -5.928^***^ | 0.675^***^ | 1.444^***^ | -3.685^***^ |
|  |  | (0.049) | (0.072) | (0.332) | (0.461) | (0.131) | (0.195) | (0.877) |
| Nov-20 |  | -0.835^***^ | -1.119^***^ | -6.773^***^ | -9.276^***^ | 1.595^***^ |  |  |
|  |  | (0.058) | (0.089) | (0.410) | (0.596) | (0.131) |  |  |
| Jan-21 |  | -0.953^***^ | -1.276^***^ | -7.979^***^ | -10.713^***^ | 1.484^***^ | 1.740^***^ | -3.320^***^ |
|  |  | (0.065) | (0.096) | (0.425) | (0.627) | (0.140) | (0.202) | (0.792) |
| Mar-21 |  | -0.837^***^ | -1.059^***^ | -7.333^***^ | -9.609^***^ | 1.076^***^ |  |  |
|  |  | (0.059) | (0.087) | (0.418) | (0.607) | (0.135) |  |  |
| First degree # Apr-20 | | 0.305^***^ | 0.631^***^ | 5.171^***^ | 9.355^***^ | 0.094 | -0.163 |  |
|  |  | (0.079) | (0.126) | (0.551) | (0.752) | (0.188) | (0.283) |  |
| First degree # May-20 | | 0.181^**^ | 0.356^***^ | 3.562^***^ | 6.549^***^ | 0.205 | -0.156 | -1.100 |
|  |  | (0.064) | (0.102) | (0.548) | (0.739) | (0.195) | (0.290) | (1.018) |
| First degree # Jun-20 | | 0.226^***^ | 0.351^***^ | 2.118^***^ | 3.916^***^ | 0.131 | -0.198 | -0.290 |
|  |  | (0.063) | (0.095) | (0.548) | (0.724) | (0.198) | (0.289) | (1.004) |
| First degree # Jul-20 | | 0.168^*^ | 0.255^*^ | -0.379 | 1.504^*^ | -0.001 |  |  |
|  |  | (0.067) | (0.103) | (0.551) | (0.726) | (0.182) |  |  |
| First degree # Sep-20 | | 0.213^**^ | 0.324^**^ | 0.324 | 0.237 | 0.177 | -0.260 | -1.895 |
|  |  | (0.067) | (0.098) | (0.492) | (0.664) | (0.185) | (0.240) | (1.121) |
| First degree # Nov-20 | | 0.274^***^ | 0.420^***^ | 2.774^***^ | 4.099^***^ | 0.256 |  |  |
|  |  | (0.076) | (0.113) | (0.515) | (0.726) | (0.194) |  |  |
| First degree # Jan-21 | | 0.347^***^ | 0.566^***^ | 3.349^***^ | 4.878^***^ | 0.446^*^ | -0.279 | -1.705 |
|  |  | (0.085) | (0.126) | (0.571) | (0.814) | (0.202) | (0.259) | (1.073) |
| First degree # Mar-21 | | 0.271^***^ | 0.398^***^ | 3.643^***^ | 5.032^***^ | 0.234 |  |  |
|  |  | (0.077) | (0.114) | (0.531) | (0.760) | (0.195) |  |  |
| Living with a partner | | 0.029 | 0.034 | 1.103 | 1.322 | 0.242 | 0.149 | 0.469 |
|  |  | (0.066) | (0.102) | (0.580) | (0.738) | (0.177) | (0.308) | (1.577) |
| Child<=15yrs | | -0.143 | -0.263 | -1.935^*^ | -2.151 | 0.079 | 1.091^*^ |  |
|  |  | (0.150) | (0.252) | (0.925) | (1.292) | (0.211) | (0.451) |  |
| COVID test result (ref: No test) | |  |  |  |  |  |  |  |
|  | Positive | -0.238 | -0.432 | -4.581^***^ | -5.011^*^ | 0.915^**^ | -1.412^**^ | 0.720 |
|  |  | (0.248) | (0.468) | (1.345) | (2.412) | (0.288) | (0.513) | (1.207) |
|  | Negative | 0.157^***^ | 0.197^**^ | 0.910^**^ | 0.961^*^ | 0.011 | -0.426^**^ | -0.381 |
|  |  | (0.039) | (0.063) | (0.298) | (0.459) | (0.107) | (0.159) | (0.758) |
|  | Pending | -0.142 | -0.098 | -1.324 | -1.438 | 0.271 | 0.752 | 5.819 |
|  |  | (0.218) | (0.295) | (1.183) | (1.684) | (0.287) | (0.638) | (3.642) |
|  |  | 7.217^***^ | 7.217^***^ | 34.600^***^ | 34.005^***^ | 11.546^***^ | 8.598^***^ | 16.498^***^ |
| Constant | | 7.211^***^ | (0.077) | (0.119) | (0.561) | (0.697) | (0.141) | (0.249) |
|  |  | -1.030^***^ | -1.721^***^ | -14.617^***^ | -21.235^***^ | 1.329^***^ | 3.310^***^ |  |
| R2 | | 0.022 | 0.035 | 0.051 | 0.092 | 0.003 | 0.028 | 0.003 |
| Within R2 | | 0.039 | 0.067 | 0.127 | 0.208 | 0.021 | 0.046 | 0.021 |
| Between R2 | | 0.033 | 0.060 | 0.019 | 0.047 | 0.001 | 0.033 | 0.000 |
| Rho | 0.615 | 0.609 | 0.589 | 0.584 | 0.613 | 0.627 | 0.660 | 0.659 |
| Number of individuals | | 8621 | 5339 | 9047 | 5631 | 11043 | 10946 | 4542 |
| Number of person-years | | 52710 | 30885 | 58306 | 34367 | 70363 | 48364 | 14895 |

Data: UKHLS & Understanding Society Covid survey waves 1-8.

Note: * p<0.05 ** p<0.01 *** p<0.001
