# Supplementary material for: The varying impacts of COVID-19 and its related measures in the UK: A year in review
Source: PLoS One. 2021 Sep 29;16(9):e0257286. doi: 10.1371/journal.pone.0257286 (PMC8480884; doi:10.1371/journal.pone.0257286)
Supplement: Attachment — Submitted filename: PONE-D-20-32060_marked up.pdf [file pone.0257286.s006.pdf]

The varying impacts of COVID-19 on labour earnings, time use and well-being at different stages of the United Kingdom lockdown

Abstract

We examine how the impact of the United Kingdom (UK) Covid-19-induced lockdown measures on different social groups had developed at different stages of the lockdown. We analyse longitudinal data from the latest UK Household Longitudinal Survey (UKHLS) COVID study and earlier waves of the UKHLS to investigate changes in people's labour income, time use, and subjective well-being across three key stages of the pandemic—before, during, and just after the ease of the lockdown period (before March, April, and , June 2020). We find that the UK Covid-19-induced lockdown measures have made unequal and varying impacts on people's income, time use, and subjective well-being according to their gender, parental status, ethnicity, and educational level in addition to the lockdown stage. In conclusion, different vulnerabilities of different social groups to the lockdown measures not only affect the extent of the Covid-19 impact, but also the speed at which these effects develop.

Key words: COVID-19; lockdown; Gender; Race; Ethnicity; Income; Time use; Wellbeing

Introduction

The massive lockdown measures introduced in March 2020 slowed the spread of COVID-19 virus in the United Kingdom (UK), but these measures have also greatly changed people's lives. When people were instructed to stay at home and maintain social distancing, the lives of millions of people were affected. For months, many people were unable to go to work and school, nor could they meet with friends and relatives. Six months after the UK announced the lockdown on 23 March 2020, our lives are far from what they were before the lockdown, and the pandemic is still not over.

Research on crises and disasters, including natural disasters and outbreaks of diseases, has shown that different social groups are impacted differently. For example, men's income tends to recover faster than women's after suffering declines in a disaster because of their higher level of economic resources before the disaster [1,2]. Minority ethnic groups, such as Black and Latina women in the US, are more likely than White Americans to suffer from residential instability due to natural hazards [3]. Richer households suffer a greater decline in income following a disaster [4] but are less likely to go bankruptcy than the less wealthy ones[5].

Recent evidence shows that the COVID pandemic and the related measures, such as social distancing and business closure, have differential impacts on various social groups. In the

UK, for example, women and parents are found to experience a larger reduction in subjective wellbeing [6–8]. Black, Asian and minority ethnic (BAME) migrants are more likely to experience economic hardship right after the lockdown [9]. Another study highlights that British men and whites in middle-income jobs are more likely to experience job loss and economic hardship [10]. In the United States, a larger negative COVID impact on mothers' paid work time is noted [11]. Regarding domestic work, some studies reported that in the UK and Australia, the increases in men's domestic work time are slightly higher [12]. In developing countries, evidence supports an enlarged social ineqaulity related to this pandemic [13].

These earlier findings confirm the existence of immediate unequal impacts, but our understanding of the longer-term impacts of COVID-19 remains limited. Past research was often confined by cross-sectional data or data that cover changes in behavior in two time points—before and after the event. Little is known as to how the unequal social impacts reveal themselves at different stages of the pandemic. This omission hinders our understanding of how COVID-induced social policies, such as the social distancing measures and the closure of certain business, which have been changing on a weekly or even daily basis, affect people's lives in a progressive way. Understanding the development of the impacts of the Covid and Covid-induced policy impact is critical for this fast-developing pandemic.

We need a more comprehensive and up to date research on how inequalities change when the COVID-19 pandemic and its corresponding measures keep on changing. In this paper, we contribute to the COVID-19 research by providing a dynamic picture of how people's labour earnings, time use, and wellbeing have varied across different stages of the lockdown. We conduct analyses on data from the latest UK surveys collected before the lockdown, right after the lockdown in March, during the lockdown period, and just after the ease of the lockdown in June. We investigate how initial inequalities by gender, ethnicity, educational level, and parental status, might have been altered at different stages of the lockdown.

In what follows, we first outline the development of the COVID-19 pandemic and the lockdown measures in the UK up to the point of late June 2020. We then introduce the data and its longitudinal design, which enables us to compare the information of the same individuals before, during, and after the lockdown. Our study measures changes in people's behaviours and feelings pre-, during, and after lockdown. We further test the robustness of our findings by using weights and the Heckman selection method to address the possible sample selection bias.

Timeline of the lockdown measures in the UK

On 31 January 2020, the first two positive cases of COVID-19 were confirmed in the UK. On 5 March 2020, the first patient who were tested positive for COVID-19 died. On 23 March 2020, the Prime Minister placed the UK on lockdown to slow down the outbreak of this pandemic. These measures included social distancing, school closure, work from home, and the close of non-essential business. Key sectors include health and social care, education

and childcare, key public services, etc., were allowed to operate if needed. To maintain employment and to protect individuals and businesses from economic harship, a Coronavirus Job Retention Scheme was implemented for the period between late March and the end of October 2020 to cover 80 percent of the regular salary of furloughed employees, up to a maximum of £2,500 per month [14]. In May, phased reopening of shops and schools were announced, and those who were unable to work from home could go back to their jobs. Since 1 June, schools are open for all Reception, Year 1 and Year 6 pupils. All shops were allowed to reopen on 15 June, though the pandemic has far from over. Until 25 July 2020, the recorded death number related to Covid-19 has reached 45,999 in the UK.

Data and methods

Data and sample

We use data from the first four waves of the UK Household Longitudinal Survey (UKHLS) COVID study survey data and preceding waves of the survey [15]. UKHLS is a household panel survey and started its first wave in 2009 with a nationally representative sample of 51,000 adults (aged 16 and above) from approximately 40,000 households. Individuals were followed up annually and were interviewed face-to-face. This research is completely based on analyses of the secondary anonymous data—the UKHLS data, which are publicly available through the UK Data Service (Study number: 6614 and 8644).

Households who participated in previous UKHLS surveys were contacted to fill in a monthly online questionnaire since April 2020. Participation in the survey was voluntary. 17,452 respondents (aged 16 and above) completed this survey with a response rate of 38.7%. Currently, data of the three waves of surveys conducted in April, May, and June are available.

Our analytical sample contains individuals who have participated in the UKHLS survey and at least one of the three waves of the Covid study. Those respondents all have access to the internet to participate in the surveys. These factors might cause a sample selection bias. In a supplementary analysis, the Covid sample is found to be socioeconomically advantaged in terms of employment, occupation, education, and home ownership compared to the full UKHLS sample. They are also more likely to be in a partnership. If we assume that one's socioeconomic status has a protective effect on the negative consequences of the lockdown measures, the reported results may underestimate the potential negative impacts of the lockdown. Nonetheless, one paper discusses this issue of non-random sample selection and indicates that the bias due to sample selection should be very limited once weight is considered [16].Please refer to the "Analytical strategies" section for a more detailed discussion.

We focus on the impact of the lockdown on income, time use, and wellbeing. Our sample includes respondents at prime working age (between 20 and 65) in 2020. 2% of the UKHLS Covid sample have missing values in the predictors to be used in regressions. The number of observations with no missing predictors are 9497, 8126, and 7496 in the first, second, and third waves of the Covid sample. The final sample for each regression varies and is

dependent on the outcome variables with non-missing values and the selection of subgroups (working or parent sample). Please refer to **Supplementary Table A1** for more details of the sample selection. The focus on changes in the outcome variables indicate that the respondents usually need to be followed up for more than one waves. Previous analyses using the same data and selecting individuals interviewed for more than one wave does not find that this selection would bias the results [17].

Measures

Monthly labour income, weekly paid work hours, weekly housework hours, weekly childcare hours, and subjective wellbeing are the five outcomes we are interested.

Change in monthly labour income

Respondent's labour income in January or February 2020 (before the lockdown) was collected retrospectively in the Covid survey. Respondents also provided their current labour income in each month (April, May, and June). We calculate the percentage change in the labour income by dividing the difference between the current and the previous monthly labour income with the previous montly labour income. This value ranges from minus one (the loss of all labour income) and positive two (capped, less than 1.5% of the sample) and is missing if this person was not working in the previous month. Accordingly, only those who worked in previous wave are included.

Change in weekly paid work hours

Respondents reported their current paid work hours per week and their working hours in January or February 2020 retrospectively. We derive the difference between the current month and the previous month. Those who have no job or are not working are assigned the value of zero. This analysis includes the full Covid sample.

Change in weekly housework hours

Respondent's housework hours were collected by the question "Thinking about last week, how much time did you spend on housework, such as time spent cooking, cleaning and doing the laundry?". We calculate the difference in weekly housework hours between the current and the previous months. Information before the Covid survey is derived from the earlier UKHLS wave (collected in the years between 2014 and 2016). We calculate changes in housework hours between different waves of the COVID survey and between the first wave of the Covid survey and the earlier waves of UKHLS in 2014 or 2016.

Change in weekly childcare hours

Respondent's childcare hours were collected by the question "About how many hours did you spend on childcare or home-schooling last week?". This information is only available in the Covid survey. We thus calculate the changes in childcare hours in two episodes, from April to May (during the lockdown) and from May to June (during and shortly after the ease of the lockdown).

Change in subjective wellbeing

Subjective wellbeing is the mental wellbeing reported by the respondents. The value is the sum of 12 items scored on a Lickert scale from 0 to 3: “ability to concentrate”, “losing sleep”, “playing a useful role in life”, “capability of making decisions”, “feeling under stress”, “overcoming difficulties”, “ability to enjoy activities”, “ability to face problems”, “feeling unhappy or depressed”, “losing confidence”, “believing in self-worth”, and “feeling generally happy”. The overall scale ranges from 0 (most distressed) to 36 (least distressed). The change is defined as the difference in the scores between the current and the previous month.

Independent variables

The key socioeconomic independent variables are constant across the waves. These variables are gender and parental status (childless men 31%, childless women 33%, fathers 16%, and mothers 20% of the sample), whether being Black, Asian or other minority ethnic (BAME) or not (reference group: whites 90%), and educational level (General Certificate of Secondary Education or lower (GCSE) 31%, higher secondary 36%, university degree or above 33%). Parents are defined as those who have children under the age 16 in the household.

All models control for respondents’ age (centered at the mean, approximately 42) and its squared form. We do not include Covid-related variables, such as whether the respondents were furloughed and whether they were keyworkers because the primary aim of this article is to document the total impact of Covid disregarding specific policies that could mediate some of these effects.

Variables used for Heckman selection

For the Heckman sample selection models (to be discussed in more details next), variables used to predict whether the respondent is included in the Covid survey are gender and parental status, ethnicity, educational level, age, whether born in the UK, whether partnered, whether have children under the age of 16, logged net household income, home-ownership status (rent, owned outright, owned with mortage, others), regions in the UK (London, North, Yorkshire and Humber, Midlands, East, South, Wales/Scotland/Northern Ireland), and the duration from the last interview to April 2020.

Analytical strategies

We apply Ordinary least squares (OLS) regressions to predict how the change in income, time use, and wellbeing differ across different sociodemographic groups and during different periods of the national lockdown (April vs Pre-lockdown, May vs April, and June vs May). We make three comparisons: before the lockdown and right after the lockdown in April, right after the lockdown in April and during the lockdown period in May, during the lockdown period in May and the gradual ease of the lockdown measures in June. The outcome variables compare the information reported by the same individuals in each time point, and hence reveal within-person changes across the lockdown periods. This analytical approach enables us to investigate trajectories of the lockdown impacts.

The key variables of interest are gender (and parental status), ethnic minority status, and level of education. These variables are constant across waves for each individual. The models examine within-person changes in the outcome variables by the aforementioned socio-economic statuses. Robust standard errors are used to consider clustering at the household level. Weights are used to take into account of unequal selection probabilities and differential non-response to approximate a population-level analysis.

The Covid sample may be biased because respondents who participated in the online survey were a selected group. The Covid sample respondents had good access to the internet, had the time and motivation to visit the website, and were determined to fill in and complete the survey [18].The application of weights could re-balance the sample to make them comparable to a national representative sample. The use of weights is recommended by the user guide (pp. 18 of Understanding Society COVID-19 User Guide v1.0) and Benzeval et al. [16]. However, some sample selection biases may also be derived from observed and unobserved characteristics that are not used in generating these weights. Therefore, the problem that the biases are correlated with both the selection and the outcome cannot be completely solved by using weights. Estimates of the associations between variables are inaccurate if the selection bias problem is not addressed properly.

When there is a full sample of observation including both participants and non-participants, selection models are a useful technique to correct the bias. Wherein this study, we have the full UKHLS sample and 38.7% of them participated in the Covid online survey. We follow previous research that aims to solve similar sample selection issues by using the Heckman selection method [19,20]. The two step Heckman method involves the estimation of a probit model for sample selection (the selection equation), and then using the resulted inverse Mills ratio to predict the outcome of interest. We use the Heckman maximum likelihood method, which combines the two steps in one with a maximum likelihood model and is shown to be more efficient. We test the performance of the Heckman method by reporting results using weights and results using Heckman method and the number of iterations in Heckman models and the p-value for comparison test. The Heckman approach is considered appropriate if the models can be easily converged and the p-value for comparison test is smaller, indicating whether selection matters [21].

These analyses were conducted in Stata/SE 16.1. Replication codes are available at https://github.com/jomuzhi/ukcovidunderstandingsociety

Results

Descriptive results

We first report the basic statistics regarding the key outcomes in **Table 1**. First, among those who worked before the lockdown, there was a clear drop in their income in April. Their income recovered in May, which should be mainly driven by the implementation of the Job Retention scheme. Some workers, such as those on construction sites, also went back to work since May. The drop in working hours was evident and persisted throughout this period, though working hours recovered gradually since May. People's housework

hours increased and reached the highest point in April and May. The average childcare hours per week reached 21 hours for parents in April, but this number gradually declined in the following periods. There was a small but continuous drop in people's subjective wellbeing since the lockdown.

Table 1. Summary statistics of income, time use, and subjective wellbeing in each wave

UK lockdown period	Monthly labour income (GBP) of people who worked in previous wave		Weekly working hours		Weekly housework hours		Weekly childcare hours		Subjective wellbeing	
	Sample with no missing value in predictors									
	Mean	SD	Mean	SD	Mean	SD	Mean	SD	Mean	SD
Before lockdown	1718.53	(1277.07)	26.83	(18.01)	8.95	(8.20)	NA	NA	24.99	(6.09)
Apr-20	1678.27	(1219.95)	17.76	(19.30)	12.47	(10.42)	20.79	(28.09)	23.87	(6.49)
May-20	1729.51	(1380.79)	19.12	(19.42)	12.34	(10.39)	19.95	(28.06)	23.86	(6.51)
Jun-20	1712.99	(1203.96)	20.68	(19.35)	11.54	(10.34)	16.65	(24.85)	23.82	(6.57)

Change in labour income

Fig 1 reports the change in monthly labour earnings. Estimates of the constant (Const.) show that, for the reference group, who are white, childless men with GCSE level of education, there was a 11-13% drop in people's earnings right after the lockdown in April, depending on the model; however, there was a 13% increase from April to May, and the earnings stayed stable from May to June.

Concerning gender and parenthood status, the differences in the changes in labour earnings are not statistically significant in each period across different gender and parental groups.

BAME groups suffer a 6-9% larger decline in labour earnings than whites right after the lockdown in March. From April to June, there was no statistically significant difference in the changes in labour earnings between BAME group and the White. Therefore, the initial larger loss of labour income of the BAME group was not compensated later.

Among the educational groups, the drop in labour earnings just after the lockdown was 4-7% less for university degree holders. Corresponding to this more limited income reduction, the income increase for the more educated group was smaller during the period from April to May. This finding implies that the Job Retention Scheme should have covered a larger group of people with less education.

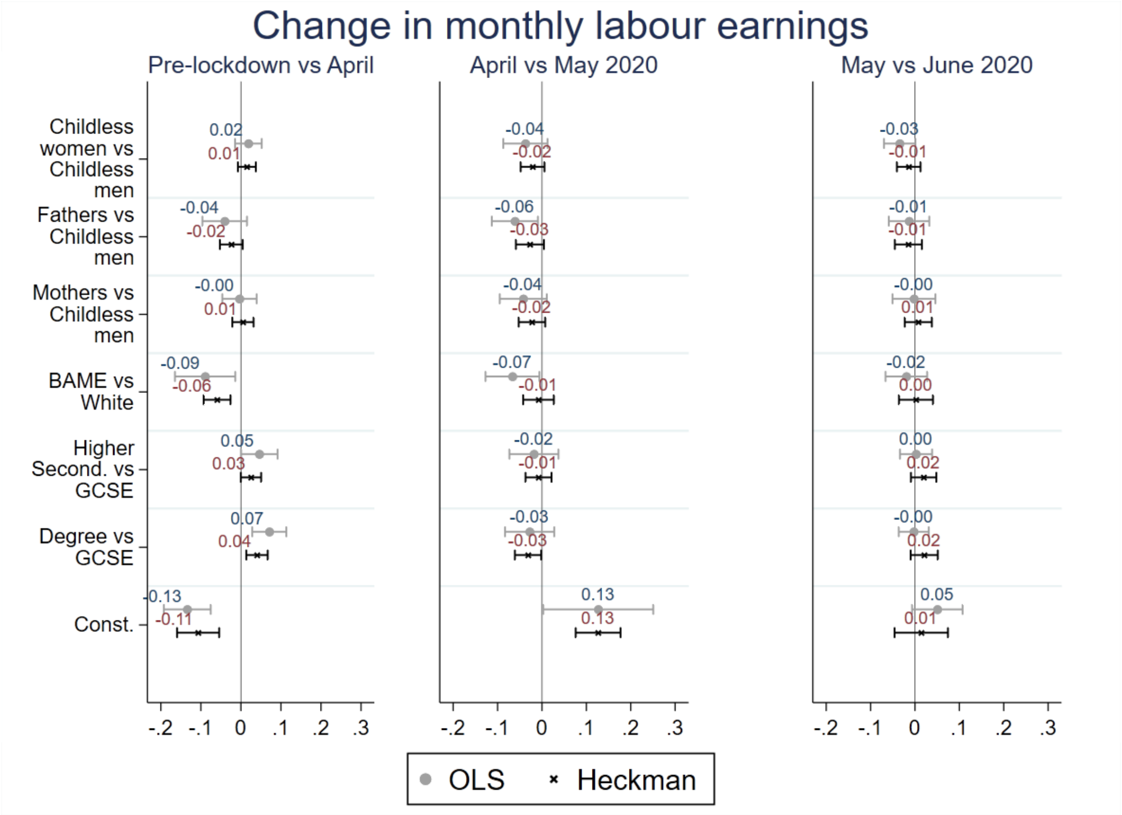

Fig 1. Predicted changes in monthly labour earnings with 95% confidence interval

Change in paid work hours

Fig 2 reports changes in people's paid work hours across different periods of the Covid pandemic.

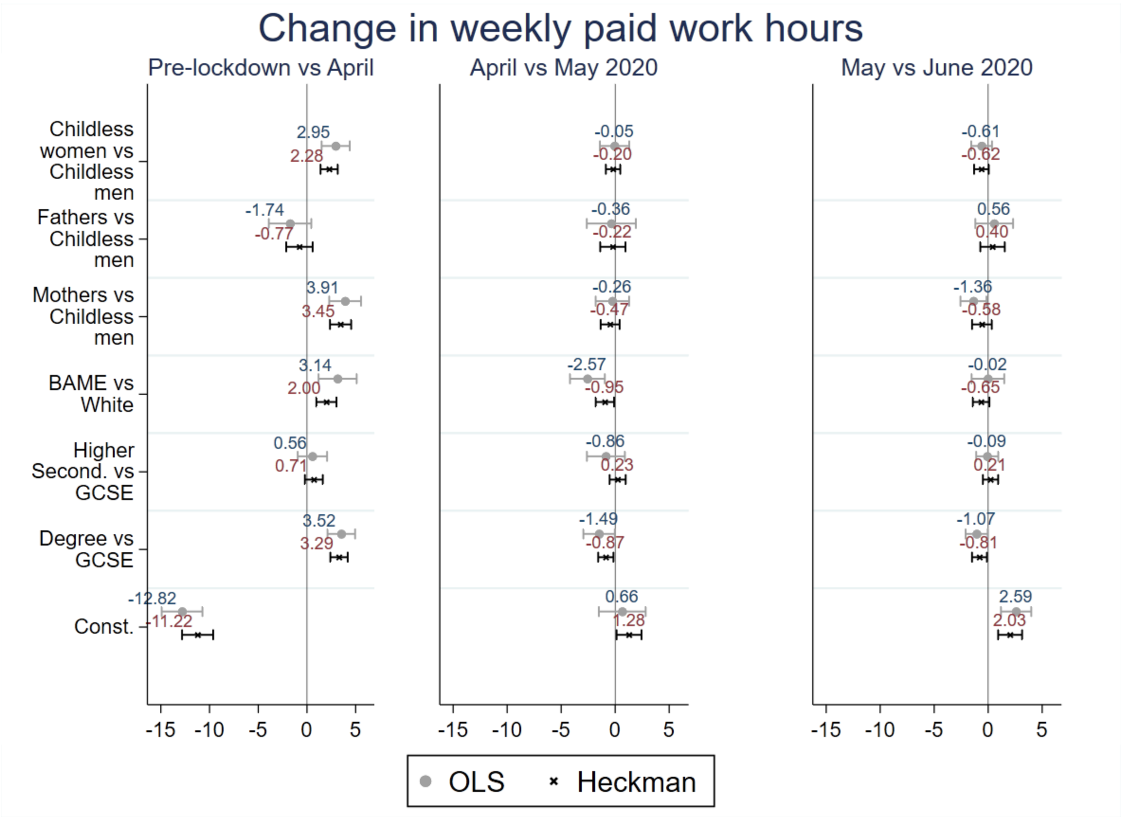

Fig 2. Predicted changes in weekly paid work hours with 95% confidence interval

First, for the reference group, who are white, childless men with GCSE level of education, there was a huge drop in paid work hours at the level of approximately 12 hours per week (estimates of the constant) just after the lockdown. For this group, the paid work time increased by about one hour between April and May; and it further increased by approximately 2.5 hours per week between May and June, reflecting a gradual process of business reopening. But, the paid work time in June did not catch up with the pre-lockdown level.

Childless women's reduction in paid work time was 2.3-3 hours less than childless men just after the lockdown. Likewise, mothers' decline in paid work time is 3.5-3.9 hours less compared with childless men. Parental status made no significant difference in the changes in men's paid work time. In our supplementary analysis, we include whether the respondent is a key worker in our models, and the gender difference disappears. The larger proportion of women working in health and social care sectors should be the reason for their more limited reduction in paid work hours.

In the following periods, there was no substantial difference in the change in paid work time by gender and parental status. Nonetheless, compared with childless men, the increase was slightly smaller for childless women and mothers (0.6 hour less), though these differences are not statistically significant at the 0.05-level.

Just after the lockdown, the reduction in paid work time was 2.0 to 3.1 hours less for BAME groups than for whites. Hu [9] reports the same finding regarding this intial change. We

further tested whether it could be the reason that a larger proportion of BAME were working in key sectors by including the key worker variable. The gap persists even when the key worker status is considered. In the following periods, when other groups' paid work time bounced back, the increase in BAME groups' paid work time was smaller. In conclusion, the BAME groups' paid work time displayed a lagged response of the lockdown measures—compared to other groups, their working hours were less responsive to the various lockdown measures. Whether this phenomenon was related to the higher COVID-19 infection rates among BAME groups warrants further investigation.

More educated people experienced a smaller drop in their paid work time just after the lockdown. People with university level of education experienced 3.3-3.5 hours less in the decline in paid work time than people with GCSE after the lockdown in April. Correspondingly, their paid work time recovered slightly ~~slower~~ in the following periods.

In sum, people who experienced less~~er~~ decline in paid work time just after the lockdown, including, women, the BAME groups, the more educated people, also experienced a smaller increase in paid work time when the lockdown measures had eased. Notably, the reasons behind this phenomenon diffe[illegible]r different groups.

Change in housework and childcare hours

Fig 3 the changes in housework time in different periods of the lockdown.

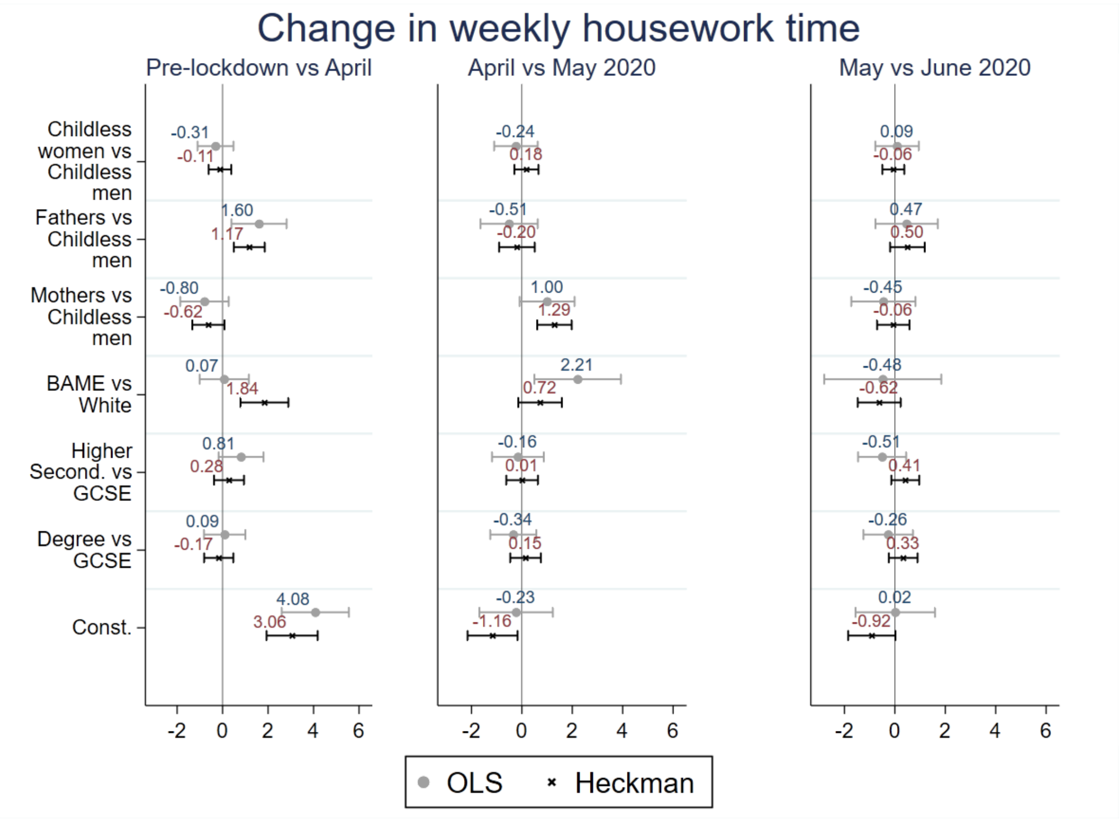

Fig 3. Predicted changes in weekly housework hours with 95% confidence interval

Estimates of the constant term show that right after the lockdown, for the reference group, there was a 3 to 4-hour increase per week in housework time. Over the following two periods, people start to go back to their pre-lockdown housework routine. From April to May, there was a 1.2-hour reduction in housework time as the Heckman selection model indicated. From May to June, there was a further reduction of 0.9 hour.

Parental status made a difference to the changes in housework time. First, there were no significant differences in the change in housework time across the three periods between childless women and childless men. Compared to childless men, fathers had a 1.2 to 1.6-hour further increase in housework time just after the lockdown. The increase in housework time was instead 0.6 to 0.8 hour less for mothers than for childless men, just after the lockdown. However, mother's housework time caught up in May: when the population average housework time decreased from April to May, the reduction was 1 to 1.3 hour less for mothers. Overall, there was little change in the gender gap in housework time if we consider the entire lockdown period.

Interestingly, people from the BAME groups had a larger increase in housework time than whites. This is reflected either from the Heckman selection model result right after the lockdown or the weighted model result in the April vs May model.

We do not observe significant differences in the changes in housework time across different educational groups, although more educated people experienced a smaller drop in paid work time in **Fig 2**.

In **Fig 4**, we restrict our sample to parents for our analyses of the changes in childcare time. The data source does not contain information about childcare time before the lockdown, and therefore we cannot make pre- and after lockdown comparisons in childcare time.

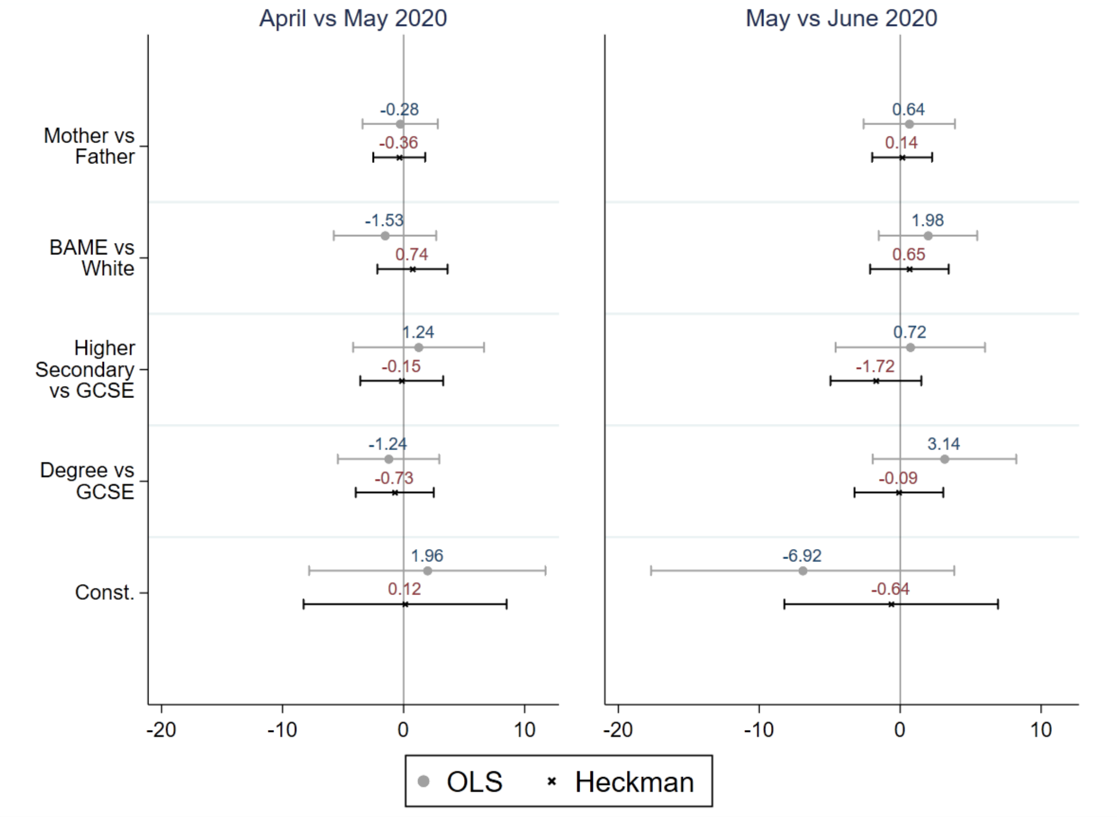

Fig 4. Predicted change in weekly childcare time among parents with 95% confidence interval

From April to May, we do not find any significant changes in childcare time. There was a tendency of reduced childcare time from May to June. There was no evidence showing that childcare time changed differently based on their gender, ethnicity, and educational levels. Overall, people's childcare time remained stable from April to June. This finding is within our expectation given that nurseries and schools remained closed to a vast majority of children in these periods.

Changes in subjective wellbeing

It is important to assess how Covid-19 and the lockdown measures have impacted people's well-being. **Fig 5** shows the changes in the reported subjective well-being by different groups during different periods.

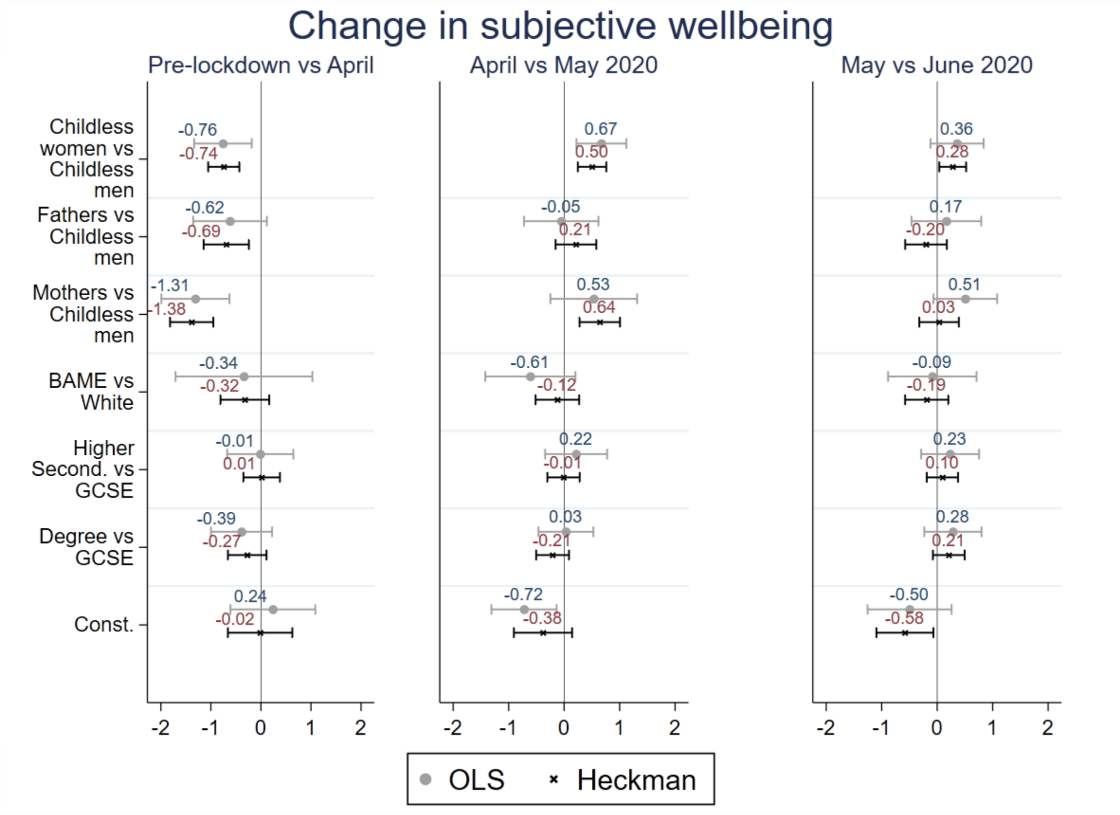

Fig 5. Predicted change in subjective wellbeing with 95% confidence interval

First, the intercept estimates reveal that for the reference group, who are white, childless men with GCSE level of education, there was no significant change in subjective well-being just after the lockdown. However, their subjective wellbeing started to decrease between April and May and then further declined between May and June. These patterns suggest that lockdown measures might have a lagged effect on subjective well-being for this group, such that the negative impacts of the lockdown measures were only observed from May rather than just after the lockdown, and that subjective well-being continued to decline between May and June despite the ease of the lockdown measures.

Gender and parental status made a difference to changes in subjective well-being. Compared to childless men, women and especially mothers, experienced a more severe decline in subjective well-being (the coefficients of the Heckman selection model are -0.74 and -1.38 respectively). Fathers also suffered a greater decline in subjective well-being than childless men. Similar findings were noted in previous studies in the UK considering this intial change [7,8]. Our study futher shows that the well-being of women, including that of mothers, bounced back to some extent between May and June, but did not return to the pre-lockdown level. These results show that women and mothers reacted faster and more strongly in their subjective wellbeing to the Covid pandemic and the lockdown measures than other groups. These changes in subjective wellbeing did not vary significantly according to ethnicity and educational level.

Overall, results from the Heckman selection model and the weighted regressions are very similar. For more details of the results and the comparison of the results between the Heckman selection model and the weighted regressions, please refer to the **Supplementary Tables A2 to A6**.

Discussion and Conclusion

In this article, we utilize the latest UK Covid panel data to provide a comprehensive analysis of how the Covid-19 lockdown measures have made unequal and dynamic impacts on different social groups at different lockdown stages. This work surpasses past studies by providing new insights into whether the inequalities in the changes in income, time-use and well-being are likely to be long-lasting or temporary.

With the gradual relaxation of the lockdown measures and the implementation of the Job Retention Scheme, we observe a recovery in labour earnings and paid work time. However, the restoration of paid work time in June 2020 did not make up for the decline observed since the pre-lockdown level. It remains uncertain as when and whether the working pattern will return to the pre-lock down level.

After the initial sharp increase in housework time, it gradually decreased during and after the ease of the lockdown, suggesting the changes in housework time and the domestic division of labour in the household might not be long lasting. Unfortunately, the data source does not allow us to observe the changes in childcare time just after the lockdown. We can see from the current data that childcare time remained relatively high and stable during and just after the lockdown. We need further waves of data, especially data collected after the summer vacation, to shed light on the continual changes in time use on paid work, housework and childcare when more nurseries and schools have been open.

Childless women and mothers experienced less reduction in their paid work hours, which is due to the reletively high proportion of them working in key sectors, especially in the health and social care industry. Women have made an important contribution to fight against the Covid by working at key sectors during the entire lockdown period [6]. This finding mirrors an earlier work that identifies a higher chance of economic hardship of British men [10] but contrary to a study in the United States, which shows that mothers experience a larger reduction in paid work time [11].

BAME people experienced a larger labour income loss than White people just after the lockdown, and this loss persisted in the following two months. We also observe a lagged reduction in paid work time for the BAME groups, which may be associated with their higher COVID-19 infection rates. The finding that BAME group's less responsive working hours and larger labor income loss corroborate the suspect that BAME groups are less likely to be supported by the government's Job Retention Scheme than whites [22].

The pre-lockdown gender gap in housework time persisted during the lockdown period. Although right after the lockdown in April, fathers increased their contribution in housework and the gender gap was reduced by approximately one hour, mothers' increase in housework time soon caught up in May. This finding contributes to our understanding

about the Covid-related gender gap in domestic work by showing that men's growing contribution of housework in the UK, highlighted in some of the earlier work [12], should only be temporary. Overall, there was little change in the gender gap in housework despite the huge reduction in paid work hours.

The decline in subjective wellbeing was more evident after April 2020, suggesting that for certain groups, e.g., childless men, the negative mental impacts of the lockdown took some time to manifest. By contrast, for childless women, fathers, and mothers, they experienced a faster and larger decline in wellbeing than childless men right after the announcement of the national lockdown measures. These findings again contribute to the literature by highlighting that Covid and the Covid-induced lockdown measures affects the wellbeing of different groups at different paces, which is largely determined by their gender and parental status.

To conclude, our findings suggest that the pandemic and its related lockdown measures have made varying degrees of negative impacts on labour earnings, time use and subjective well-being at different stages of the lockdown. We need further research to understand the reasons and to monitor how the pandemic might further exacerbate inequalities by gender, parental status, educational level, and ethnic minority status. Overall, this study makes an important contribution to our understanding of the impact of this pandemic by highlighting that the different vulnerabilities of different social groups to the Covid-induced lockdown measures not only affect the extent of the Covid impact, but also the speed at which these effects develop.

References

1. Bradshaw S. Socio-economic impacts of natural disasters: A gender analysis. United Nations Economic Commission for Latin America and the Caribbean. 2004. Available: https://www.cepal.org/en/publications/5596-socio-economic-impacts-natural-disasters-gender-analysis

2. Neumayer E, Plümper T. The gendered nature of natural disasters: The impact of catastrophic events on the gender gap in life expectancy, 1981-2002. Annals of the Association of American Geographers. 2007;97: 551–566. doi:10.1111/j.1467-8306.2007.00563.x

3. Elliott JR, Howell J. Beyond Disasters: A Longitudinal Analysis of Natural Hazards' Unequal Impacts on Residential Instability. Social Forces. 2017;95: 1181–1207. doi:10.1093/sf/sow086

4. Keerthiratne S, Tol RSJ. Impact of natural disasters on income inequality in Sri Lanka. World Development. 2018;105: 217–230. doi:https://doi.org/10.1016/j.worlddev.2018.01.001

5. Billings SB, Gallagher E, Ricketts L. Let the rich be flooded: The unequal impact of hurricane Harvey on household debt. Social Science Research Network. 2020. Available: https://ssrn.com/abstract=3396611 or http://dx.doi.org/10.2139/ssrn.3396611

6. Zhou M, Hertog E, Kolpashnikova K, Kan M-Y. Gender inequalities: Changes in income, time use and well-being before and during the uk covid-19 lockdown. SocArXiv. 2020; 1–16. doi:10.31235/osf.io/u8ytc

7. Davillas A, Jones AM. The covid-19 pandemic and its impact on inequality of opportunity in psychological distress in the uk. SSRN. 2020. doi:10.2139/ssrn.3614940

8. Pierce M, Hope H, Ford T, Hatch S, Hotopf M, John A, et al. Mental health before and during the covid-19 pandemic: A longitudinal probability sample survey of the UK population. The Lancet Psychiatry. 2020. Available: https://doi.org/10.1016/S2215-0366(20)30308-4

9. Hu Y. Intersecting ethnic and native–migrant inequalities in the economic impact of the covid-19 pandemic in the UK. Research in Social Stratification and Mobility. 2020;68: 100528. doi:https://doi.org/10.1016/j.rssm.2020.100528

10. Witteveen D. Sociodemographic inequality in exposure to covid-19-induced economic hardship in the united kingdom. Research in Social Stratification and Mobility. 2020;69: 100551. doi:https://doi.org/10.1016/j.rssm.2020.100551

11. Collins C, Landivar LC, Ruppanner L, Scarborough WJ. COVID-19 and the gender gap in work hours. Gender, Work & Organization. 2020. doi:10.1111/gwao.12506

12. Craig L, Churchill B. Dual-earner parent couples' work and care during covid-19. Gender, Work & Organization. 2020; 1–16. doi:10.1111/gwao.12497

13. Bottan BAV-C Nicolas AND Hoffmann. The unequal impact of the coronavirus pandemic: Evidence from seventeen developing countries. PLOS ONE. 2020;15: 1–10. doi:10.1371/journal.pone.0239797

14. UK Government. Check if your employer can use the coronavirus job retention scheme. GOV.UK. 2020. Available: https://www.gov.uk/guidance/check-if-you-could-be-covered-by-the-coronavirus-job-retention-scheme

15. University of Essex, Institute for Social and Economic Research. 2020. Understanding Society: COVID-19 Study, 2020. [data collection]. 3rd Edition. UK Data Service. SN: 8644, doi:10.5255/UKDA-SN-8644-3

16. Benzeval M, Burton J, Crossley T, Fisher P, Jöckle A, Low H, et al. The idiosyncratic impact of an aggregate shock: The distributional consequences of covid-19. Understanding Society Working Paper Series. 2020. Available: https://www.understandingsociety.ac.uk/sites/default/files/downloads/working-papers/2020-09.pdf

17. Uhrig SCN. The nature and causes of attrition in the British household panel study. Colchester: University of Essex, Institute for Social; Economic Research (ISER); 2008. Report No.: 2008-05. Available: http://hdl.handle.net/10419/92025

18. Bethlehem J. Selection bias in web surveys. International Statistical Review. 2010;78: 161–188. doi:10.1111/j.1751-5823.2010.00112.x

19. Morrissey K, Kinderman P, Pontin E, Tai S, Schwannauer M. Web based health surveys: Using a two step heckman model to examine their potential for population health analysis. Social Science & Medicine. 2016;163: 45–53. doi:https://doi.org/10.1016/j.socscimed.2016.06.053

20. Chaix B, Billaudeau N, Thomas F, Havard S, Evans D, Kestens Y, et al. Neighborhood effects on health: Correcting bias from neighborhood effects on participation. Epidemiology. 2011;22: 18–26. Available: http://www.jstor.org/stable/29764675

21. Bushway S, Johnson BD, Slocum LA. Is the magic still there? The use of the Heckman two-step correction for selection bias in criminology. Journal of Quantitative Criminology. 2007. doi:https://doi.org/10.1007/s10940-007-9024-4

22. London Assembly M of L. Mayor calls for targeted extension of furlough scheme. London City Hall. 2020. Available: https://www.london.gov.uk/press-releases/mayoral/mayor-calls-for-targeted-extension-of-furlough

Click here to access/download
Supporting Information
Supplementary_PlosOne.docx
