# Supplementary material for: The varying impacts of COVID-19 and its related measures in the UK: A year in review
Source: PLoS One. 2021 Sep 29;16(9):e0257286. doi: 10.1371/journal.pone.0257286 (PMC8480884; doi:10.1371/journal.pone.0257286)
Supplement: Attachment — Submitted filename: 2020 PLOS1 32060 report.pdf [file pone.0257286.s008.pdf]

Report on: The Varying Impacts of COVID-19 on Labor Earnings, Time Use and Well-Being at Different Stages of the United Kingdom Lockdown (MS# PONE-D-20-32060)

As the title suggests, the paper quantifies changes in well-being for a national sample during April-June 2020 when COVID-19 and related policy measures had major impacts on United Kingdom residents. Across all measures, the paper finds worse outcomes in April, a small rebound in May and little or no impact in June. The Abstract concludes “different vulnerabilities of different social groups to the lockdown measures not only affect the extent of the Covid-19 impact, but also the speed at which these effects develop.”

The paper uses good methods to document how income and behavior changed over the focal three months but is less convincing in attributing *all* the changes to the Covid-19 pandemic and subsequent lockdown measures. In other words, the paper documents correlation but not causation. A timeline of major regulatory events and their implementation lags would have been very helpful. It seems implausible that all policy measures would have instantaneous effects as currently assumed, or that individuals would not have changed their behavior voluntarily even absent government mandates. The marked-up paper identifies minor editorial suggestions. More detailed comments follow:

1. Given the stated desire to study nationwide long-term impacts of COVID-19, it is strange that the paper relies only on stale (June 2020) survey data from the United Kingdom Household Longitudinal Survey (UKHLS) rather than supplementing with more recent unemployment data from the Office of National Statistics, available monthly through November 2020. (https://www.ons.gov.uk/employmentandlabourmarket/peopleinwork/employmentandemployeetypes/bulletins/employmentintheuk/november2020)
2. Similar tests have been published for the April and May 2020 waves of the UKHLS COVID-19 surveys. The paper adds tests for the June 2020 wave and finds little changes during this month.
3. Many variables in the Supplementary are undefined making it difficult to interpret the results. The Tables would ideally be self-contained.
4. The paper should better justify why standard errors are clustered at the household level rather than by gender or region, since the effects are theorized to covary within groups.
5. The Heckman procedure for self-selection in responses seems appropriate for the research setting. However, most of the *p*-values from the comparison tests (8 of 14) reported in the Supplementary are well above 0.10 significance level, suggesting that the

Heckman selection models have low predictive power. The paper does not discuss this low power in discussing results or drawing inferences.

6. Likewise, almost half of the OLS models (6 of 14) have model *p*-values that exceed the 0.10 significance level, indicating low reliability. Importantly, 4 of 5 models run on Wave 31 (July 2020) data are not significant at the 0.10 significance level. The reported R^2s are often close to zero, suggesting that the adjusted R2s are negative. In other words, the reported results are like using a random number generator to measure most of the independent variables in about half the tests.
7. Interpretation of the novel June 2020 results are clouded by the problem that "absence of evidence is not evidence of absence." Thus, while it is descriptively valid to say that there were no net changes in June 2020, this is not the same as saying that COVID-19 and the lockdown measures had no impact, because some measures such as the Coronavirus Job Retention Scheme may have fully offset the impact of COVID-19, but each of these components may have had large effects considered individually.
8. The paper should be more cautious in interpreting the results. Given that there may have been important policy changes beyond those related to COVID-19, as well as voluntary changes in behavior, all the paper reports is the net effect of all changes. The lack of change in June 2020 is still interesting, but at best the paper is still studying short-term or medium-term effects rather than long-term effects. Low explanatory power of the models should be discussed explicitly.
