# Supplementary material for: The varying impacts of COVID-19 and its related measures in the UK: A year in review
Source: PLoS One. 2021 Sep 29;16(9):e0257286. doi: 10.1371/journal.pone.0257286 (PMC8480884; doi:10.1371/journal.pone.0257286)
Supplement: Attachment — Submitted filename: PONE-D-20-32060_R1_marked up.pdf [file pone.0257286.s010.pdf]

Abstract

We examine how earnings, time use, and subjective wellbeing of different social groups had changed at different stages/waves of the pandemic in the United Kingdom (UK). We analyze longitudinal data from the latest UK Household Longitudinal Survey (UKHLS) COVID study and the earlier waves of the UKHLS to investigate within-individual changes in labor income, paid work time, housework time, childcare time, and distress level during the three lockdown periods and the easing period between them (from April 2020 to late March 2021). We find that as the pandemic develops, the UK COVID-19 and its related lockdown measures have made unequal and varying impacts on people's income, time use, and subjective well-being based on their gender, ethnicity, and educational level. In conclusion, the extent of the impacts of the COVID-19 and the COVID-induced measures as well as the speed at which these impacts develop, vary across social groups with different types of vulnerabilities.

Keywords: COVID-19; lockdown; Gender; Race; Ethnicity; Income; Time use; Wellbeing

Introduction

More than one year has passed since the United Kingdom (UK) officially announced its first national lockdown on 23 March 2020 due to the fast spread of the COVID-19. The massive lockdown measures temporarily slowed the spread of the COVID-19 virus in the UK but have also greatly changed people's lives. When people were instructed to stay at home and maintain physical distancing, the lives of millions of people were affected. For months, many people were unable to go to work and school, nor could they meet friends and relatives. What was unexpected was that people in the UK had experienced in total three national lockdowns over the past year. Up till now, people's lives are far from what they were before the first lockdown, and the pandemic is still not over.

Recent evidence has shown that the COVID-19 pandemic and the related social and economic measures, such as physical distancing and business closure, have made differential impacts on various social groups. In the UK, for example, women and parents are found to have experienced a larger reduction in subjective wellbeing [1,2]. Black, Asian, and minority ethnic (BAME) immigrants were more likely to experience economic hardship right after the first national lockdown [3]. In addition, among those who were known to have the COVID-19, people of BAME background in the UK had a death rate that was higher than white people [4]. As has been said in the poem: "we are in the same storm, but we are not all in the same boat."

These earlier findings confirmed the existence of immediate unequal impacts for different social groups, but our understanding of the longer-term impacts of COVID-19 and the COVID-19 induced measures remains limited. We know little about how the impacts might have changed since the first lockdown. The COVID-19 pandemic has already lasted for more than one year, and the UK has suffered from three national lockdowns.

Past research was often confined by data that covered only two time points—before and shortly after the announcement of the first lockdown, for example. Little is known as to how the unequal social impacts reveal themselves at different stages of the COVID-19 pandemic, especially with repeated lockdowns. This omission hinders our understanding of how COVID-19 and COVID-induced social policies, such as the physical distancing measures, working from home, and the closure of certain businesses, which have been changing on a weekly or even daily basis, progressively affect people's lives. Documenting the development of the impacts of the COVID-19 and the COVID-induced measures is important for us to understand the consequences of this fast-developing pandemic.

We need more comprehensive and up-to-date research on how inequalities might have changed as the COVID-19 pandemic spread out and the various measures to contain it were implemented over the past year. We conduct analyses on a national-representative population data from the latest UK Household Longitudinal Survey (UKHLS) collected before the first lockdown in March 2020, during the first lockdown from April to June 2020, the ease of the first lockdown (June to September 2020), and during the latter two lockdowns (November 2020, from January 2021 to March 2021). In this paper, we contribute to the COVID-19 research by providing a dynamic picture of how people's labor earnings, time use, and wellbeing have changed across different stages of the pandemic. We further investigate whether, and the extent to which, the inequalities on these outcomes by gender, ethnicity, and educational level, have changed over the past year.

In what follows, we first review the latest works on the impact of COVID-19 and the COVID-induced measures on people's lives, focusing on three dimensions of social inequality–gender, race/ethnicity, and education. We then outline the development of the COVID-19 pandemic and the lockdown measures in the UK from March 2020 to April 2021. Next, we introduce the data and its longitudinal design, which enables us to compare the information of the same individuals before the start of this pandemic and at different time points over the past year. Finally, we will report the results of fixed-effect regression analyses and discuss our conclusions in the end.

The impacts of the COVID-19 and its related measures

The primary aim of the COVID-19 induced measures is to contain the virus by reducing physical contacts of people. Many of these measures have immediately effect on people's behaviors, but others could have longer-term impacts. For example, the close of businesses and the work from home guidance altered people's working patterns tremendously. Reductions in paid work time and earnings have been immediately recorded in countries that have introduced lockdown measures such as Australia [5], the UK [3,6], and the United States (US) [7]. When more people stayed at home and the option of outsourcing domestic work was reduced due to business closure or the fear of contracting COVID-19, it is not surprising to see that people spent substantially more time on unpaid domestic work than they used to [5,6,8,9].

People's feelings also changed. Loss of employment, financial strain, and social isolation are some well-known factors that negatively affect mental health [10–12]. Not surprisingly, worsened subjective wellbeing was observed in Australia [5,13], the UK [14–16], and the US [17] soon after the start of the pandemic. Once the lockdown restrictions began to be lifted, people's subjective wellbeing started to recover. As Pierce et al. [16] have noted by using the first five waves of the same UKHLS COVID study data as in this paper, "Between April and October 2020, the mental health of most UK adults remained resilient or returned to pre-pandemic levels." But, "Around one in nine individuals had deteriorating or consistently poor mental health."

This COVID-19 pandemic and its related measures have raised surging concerns of exacerbated social inequalities. Since long before the pandemic, gender inequalities have existed in the labor market. In the UK, the labor force participation rate for men is higher than women, and men also are much more likely to work full time [8,18]. Women are more likely to be at-home workers. Reasons include inflexible workplace expectations, gender norms expecting men to be the primary earners and women the primary caregivers, and discrimination in the labor market. When people are required to work from home, the spatial boundary between market work and family life is blurred. Many studies have investigated whether the changes in time use due to lockdown measures are the same for women and men. Between March and May 2020 (UK 1st lockdown), British men were found to be more likely to be furloughed or dismissed from work than women [19]. However, studies focusing on the labor market performance of parents reveal a different pattern. In the UK, during the first lockdown period from April to May 2020, among parents with children aged between 4 and 15, mothers are found to be more likely to be laid off, furloughed, or quit their jobs [20]. Similarly, in Australia [5], Canada [21], and the US [22], mothers with young children experienced a larger change in their paid work time or were more likely to leave their jobs. On the other hand, several studies have reported improvements in the domestic division of labor: the increase in domestic work was larger for men than for women during the lockdown period in Australia[5], Canada [23], France [24], and the US [25]. However, contrary results were reported in Germany [26] and Spain [27]. The decline in subjective wellbeing also differs between women and men. In the UK and Australia, women were found to experience a larger reduction in subjective wellbeing than men [1,2,5,8,28].

In the UK, Black, Asian, and minority ethnic (BAME) *immigrants* are more likely to experience economic hardship just after the first lockdown [3]. BAME immigrants, compared with their white counterparts, were also found to suffer a larger decline in subjective wellbeing at the beginning of the March 2020 lockdown in the UK [3,29]. In Indiana, US, Black Americans were more than 3 times more likely to lose their job than whites [30]. In contrast, another study highlights that British whites in middle-income jobs are more likely to experience job loss and economic hardship, and this was primarily driven by the fact that many BAME people are employed in key sectors such as the health and social care services, which were exempt from the lockdown measures and instead had a surge in work demands, in the first UK lockdown [19].

People with less education and lower income suffered strongly during the pandemic. They were particularly hit hard with a higher chance to lose their job and earnings than others in countries including Canada [31], the UK [19], and the US [30]. Many of the less

educated are trapped in lower-skilled occupations with a large financial constrain. Consequently, the less educated group reported a heightened level of distress during the first lockdown in the UK [32]. However, one US study reports that the decline in subjective wellbeing was larger among the more educated, and the potential reason could be that the more educated might have felt a greater loss of control and wealth due to COVID-19-related uncertainties [17].

Again, current literature has focused extensively on the impacts of the relatively early stage of this pandemic. In particular, studies that have employed the same British data source of the present study have examined the changes in earnings, time use, and subjective wellbeing during the implementation of the first national lockdown in late March 2020, for example, [3,6,8,9,19]. Pierce et al.'s work [16] on subjective wellbeing is an exception. Their work has examined the recovery of subjective wellbeing when the first lockdown measures were eased from June to October 2020. However, their study did not cover the latter lockdowns in November 2020 and January 2021. This may be due to the different stages of this pandemic with different restrictive measures imposed by different countries. In this article, we will provide a first-year review of the COVID-19 development in the UK and document how people have responded to the first lockdown, the ease of the first lockdown, and the latter two lockdowns. This evaluation could answer whether people responded similarly to repeated lockdowns and whether these changes in earnings, time use, and feelings are temporary or long-lasting.

Timeline of the lockdown measures in the UK

On 31 January 2020, the first two positive cases of Covid-19 were confirmed in the UK. On 5 March 2020, the first patient who was tested positive for Covid-19 died. On 23 March 2020, the Prime Minister placed the UK on lockdown to slow down the outbreak of this pandemic. These measures included physical distancing, school closure, work from home, and the close of non-essential businesses, including pubs and cafes. Key sectors, which include health and social care, education and childcare, key public services, etc., were allowed to operate.

To maintain employment and to protect individuals and businesses from economic hardship, a *Coronavirus Job Retention Scheme* was implemented for the period between late March and the end of October 2021 to cover 80 percent of the regular salary of furloughed employees, up to a maximum of £2,500 per month [33]. In April, the UK had more than 10,000 deaths related to COVID-19. In May, phased reopening of shops and schools were announced, and those who were unable to work from home should return to the workplace.

From 1 June 2020, schools had been open for all Reception, Year 1 and Year 6 pupils, but the summer holiday arrived soon. Non-essential business reopened gradually from 15 June. From 4 July, pubs, cinemas, restaurants reopened. Physical distancing rules were relaxed from a "two-meter" to a "one-meter plus" rule. In August, restrictions were eased further, though the pandemic was far from over.

The UK variant of the coronavirus was first identified in September 2020, which was considered to be more transmissible and potentially more deadly. In late September, people were required to work from home with a 10 pm curfew for the hospitality sector. In October, England entered the 3-tier system where different regions were classified into different tiers depends on the level of the spread of the virus. Soon after, the second national lockdown came into force on 5 November and lasted until 2 December. People were told to stay at home. Other measures included the closure of hospitality and non-essential shops, but schools were open, and people could leave their home for outdoor exercise as many times per day. After 2 December, the UK then entered a stricter 3-tier restriction system.

However, this 3-tier system did not last long. After Scotland announced lockdown, on 4 January 2021, a third national lockdown was announced. Schools were shut again and people were urged to stay at home. This time, the measures were stricter than those in the second lockdown. They included “Stay at home at all times, wherever possible,” “Not allowed to meet others from outside your household (or support bubble),” “All retail and hospitality venues must close,” and “Personal care services have to close.” Schools were shut to most pupils, except for the children of critical workers and most vulnerable children. Nurseries were kept open.

Since 8 March, schools in the UK have completely re-opened. Non-essential retail and personal care services have reopened since 12 April. People have been allowed to meet outdoors as a number of the restrictive measures have been lifted since May 17. A complete easing will be on 19 July 2021. The Prime Minister pledged that all adults in the UK will be offered their first dose of a COVID-19 vaccine by the end of July.

Until 16 April 2021, the recorded death number related to C~~ovid~~-19 has reached over 127K in the UK. A more detailed timeline of the UK lockdowns can be found at [https://www.instituteforgovernment.org.uk/sites/default/files/timeline-lockdown-web.pdf].

Data and methods

Data and sample

We use data from the first eight waves of the UK Household Longitudinal Survey (UKHLS) COVID study data and preceding two waves (2017/18 and 2018/19) of the UKHLS main survey [34]. UKHLS is a household panel survey and started its first wave in 2009 with a nationally representative sample of 51,000 adults (aged 16 and above) from approximately 40,000 households. Individuals were followed up annually and were interviewed face-to-face. This research is based completely on the UKHLS data that are publicly available through the UK Data Service (Study number: 6614 and 8644) and are completely anonymous.

Regarding the COVID study, households who participated in previous UKHLS surveys were contacted to fill in a monthly online questionnaire since April 2020. The complementary telephone survey started in May 2020. Participation in the survey was

voluntary. Approximately 16,000 respondents (aged 16 and above) completed this first wave of the COVID survey with a response rate of 42%. Currently, data of the first eight waves of surveys conducted in the last week in April, May, June, July, September, November in the year 2020 and the last week in January and March in the year 2021 are available.

Our analytic sample contains individuals who have participated in the UKHLS main survey and at least one of the eight waves of the COVID study. The respondents all have access to the internet or telephone to participate in the surveys. This requirement might have caused a sample selection bias. In a supplementary analysis, the sample of the COVID study is found to be socioeconomically advantaged in terms of employment, occupation, education, and homeownership compared to the full UKHLS sample. If we assume that one's socioeconomic status has a protective effect on the negative consequences of the COVID-19 and related lockdown measures, the reported results may underestimate the potential negative impacts of the COVID-19 and related lockdown. Nonetheless, one paper discusses this issue of non-random sample selection and demonstrates that the bias due to sample selection is very limited once weight is considered [35]. In the following analysis, we apply the weights provided in the data, which have adjusted for "unequal selection probabilities and differential nonresponse" [36].

~~We focus on the impacts of the lockdown on five outcomes–labor income, paid work time, housework time, childcare time, and wellbeing.~~ Our sample includes respondents of prime working age (between 20 and 65) in 2020. 2% of the UKHLS Covid sample have missing values in the predictors to be used in regressions. The numbers of observations with no missing predictors are *10484, 9008, 8478, 8210, 7642, 7083, 7019, and 7525 in the first eight waves* of the COVID study. The final sample for each regression is dependent on the outcome variables with non-missing values (some outcome variables are not asked in certain waves) and the selection of subgroups (for example, people who had a job before the pandemic). Please refer to **Supporting information Table A1** for more details of the sample selection process. The focus on within-individual changes in the outcome variables indicates that the respondents should be followed up for more than one wave. Previous analyses using the same data and selecting individuals interviewed for more than one wave do not find that this selection would bias the results [37].

Measures

Monthly labor income, weekly paid work hours, subjective wellbeing, weekly housework hours, and weekly childcare hours are the five dependent variables or outcomes in which we are interested.

Monthly labor income

Respondent's labor income in January or February 2020 (before the lockdown) was collected retrospectively in the COVID survey. Respondents also provided their current labor income in each month thereafter. We calculate the natural log of the labor income. The sample selected to predict this outcome is those who had a job in January or February 2020, before the first national lockdown.

Weekly paid work hours

Respondents reported their current paid work hours per week and their usual working hours in January or February 2020 retrospectively. For the COVID-19 pandemic period, the question is “How many hours did you work, as an employee or self-employed, last week?” For the reference period, the question is “During January and February 2020, how many hours did you usually work per week?” The sample selected to predict this outcome is those who had a job in January or February 2020, the same as the above.

Subjective wellbeing

Subjective wellbeing is the mental wellbeing reported by the respondents in a General Health Questionnaire (GHQ). The value is the sum of 12 items (GHQ-12) scored on a Lickert scale from 0 to 3: “ability to concentrate,” “losing sleep,” “playing a useful role in life,” “capability of making decisions,” “feeling under stress,” “overcoming difficulties,” “ability to enjoy activities,” “ability to face problems,” “feeling unhappy or depressed,” “losing confidence,” “believing in self-worth,” and “feeling generally happy.” The overall scale ranges from 0 (least distressed) to 36 (most distressed). This measurement is a validated and widely used measure of non-specific mental distress in surveys [38]. The same information has been collected in earlier waves of the main survey of the UKHLS and in each wave of the COVID study. The full sample is used to predict this outcome.

Weekly housework hours

Respondent’s weekly housework hours were collected by the question “Thinking about last week, how much time did you spend on housework, such as time spent cooking, cleaning and doing the laundry?” Information before the COVID survey is derived from the earlier UKHLS waves (the latest one was collected in the years between 2018 and 2019). The full sample is used to predict this outcome.

Weekly childcare hours

Respondent’s childcare hours were collected by the question “About how many hours did you spend on childcare or home-schooling last week?” This information is only available in the COVID survey. Only those who had a child younger than 16 years old in the household (referred to as parents in later analyses) were asked and are used for analyses.

Independent variables

We include the wave dummies, which represent the time point when information was collected to examine the dynamics in those outcome variables.

The key socioeconomic independent variables are constant for the same individual across the waves. These variables are gender (52.2% females), whether being Black, Asian or other minority ethnic (BAM[illegible]or not (reference group: whites 95%), and educational level (university degree holders 34.7%).

All models control for respondents’ partnership status (whether they live with a partner) and parenthood status (the presence of a child younger than age 16 in the household) to

account for potential changes in the family status that are correlated with the outcomes [39,40]. We do not include COVID-related variables, such as whether the respondents were furloughed and whether they were keyworkers because the primary aim of this article is to document the *total net impact* of the COVID-19 and its related measures disregarding specific policies or the development of this pandemic that could mediate some of these effects.

Analytical strategies

We apply linear fixed-effect regressions to predict the five outcomes. By interacting the month indicator with gender, BAME group, and education levels, we examine how the change in income, time use, and wellbeing differ across individuals in the three different socio-demographic groups in different periods of the pandemic. The reference time point is January and February 2020 for the outcomes earnings and weekly paid work hours. The reference time point is the year 2018/2019 for the outcomes subjective wellbeing (distress level) and weekly housework hours. For weekly childcare hours, the reference time point is April 2020, which is during the first national lockdown. The outcome variables compare the information reported by the same individuals in each time point, and hence reveal within-person changes. This analytical approach enables us to investigate trajectories of the outcome variables over the past year conditional on the same individual.

The fixed-effect regression method takes full account of the time-constant individual characteristics that are correlated with both the independent variable and the outcome variables. They achieve this by demeaning the dependent and independent variables from person-specific means [41].

Weights are used to take into account unequal selection probabilities and differential non-response to approximate a population-level analysis. This approach has been used in previous work analyzing the same data, for example, [2,3,16,19]. Clustered standard errors are used to consider the sampling design of the UKHLS survey [42].

These analyses were conducted in Stata/SE 16.1. Replication codes are available at https://github.com/jomuzhi/ukcovidunderstandingsociety

Results

Descriptive results

We first report the weighted mean values of the key outcomes in **Table 1**.

First, among those who worked before the first lockdown (between January and February 2020), there was a clear drop in their average earnings from April 2020. Their income recovered slightly in May from the April level, which should have been mainly driven by the implementation of the *Job Retention scheme*. Some workers who could not work from home, such as those working on construction sites, also went back to the workplace in May. Since then, the average monthly net earnings were kept approximately at the level

of GBP1,550. Notably, ever since the first lockdown, people's take-home earnings never returned to their pre-pandemic level.

Table 1. Mean values of earnings, paid work hours, subjective wellbeing, housework hours and childcare hours in each month.

Months / Mean	Net earnings	Weekly working hours	Subjective wellbeing (higher value indicates higher distress level)	Weekly housework hours	Weekly childcare hours
Jan/Feb 2020 or 2017/19	1,673.9	34.7	11.8	9.0	-
Apr 2020	1,428.3	21.9	13.1	12.3	16.7
May 2020	1,570.2	23.9	13.1	12.2	15.8
Jun 2020	1,546.5	26.0	13.1	11.3	13.3
Jul 2020	1,559.7	27.1	12.4	-	-
Sep 2020	1,553.2	30.2	12.4	10.4	12.9
Nov 2020	1,573.1	29.0	13.4	-	-
Jan 2021	1,561.0	28.1	13.4	10.5	13.4
Mar 2021	1,547.6	28.6	12.9	-	-

Note: Cross-sectional baseline respondent weight applied to represent the population of all adults (16+) who were resident in private households in the UK at the time of wave 9 (2017/18), and who did not die or emigrate before the relevant web survey.

Data source: UKHLS main survey waves 9 to 10 (up to 2019) and COVID study waves 1 to 8

Before the pandemic, those who worked in January and February 2020 worked 34.7 hours per week on average. A record low of 21.9 hours per week was observed in April 2020. The persistent decline in paid work time over the past year was evident, though working hours had recovered gradually since May and reached a peak of approximately 30 hours per week in September 2020. The latter two national lockdowns (November 2020 and January 2021) did not reduce the working hours as much as the first national lockdown. Weekly paid work hours were maintained at approximately 28 hours.

People had felt more distressed from March 2020. The worst number of 13.4 was recorded in the last two rounds of lockdown–November 2020 and January 2021.

People's housework hours increased and reached the highest level of 12.3 hours per week in April and May 2020. Afterward, housework time declined gradually and was maintained at 10.5 hours per week. Compared with the figure recorded in September 2020 when most lockdown restrictions were eased, the figure in January 2021 did not change significantly, even though a stricter lockdown was in place. This finding concurs with the small drop in paid work hours from September 2020 to January 2021.

The average childcare hours per week reached 16.7 hours for parents in April, but this figure gradually declined to approximately 13 hours per week before the third national lockdown. In January 2021, childcare hours had only increased 0.5 hours per week from the September figure, even though schools were shut to most pupils during the third

lockdown. Overall, people's time use had become less responsive to the repeated lockdowns.

Changes in earnings, paid work time, subjective wellbeing, housework and childcare time

Figure 1 reports within-individual changes in the five indicators across waves. The red lines indicate the time point when the national lockdowns started to enforce. Detailed coefficients are reported in **Supporting information Table A2**.

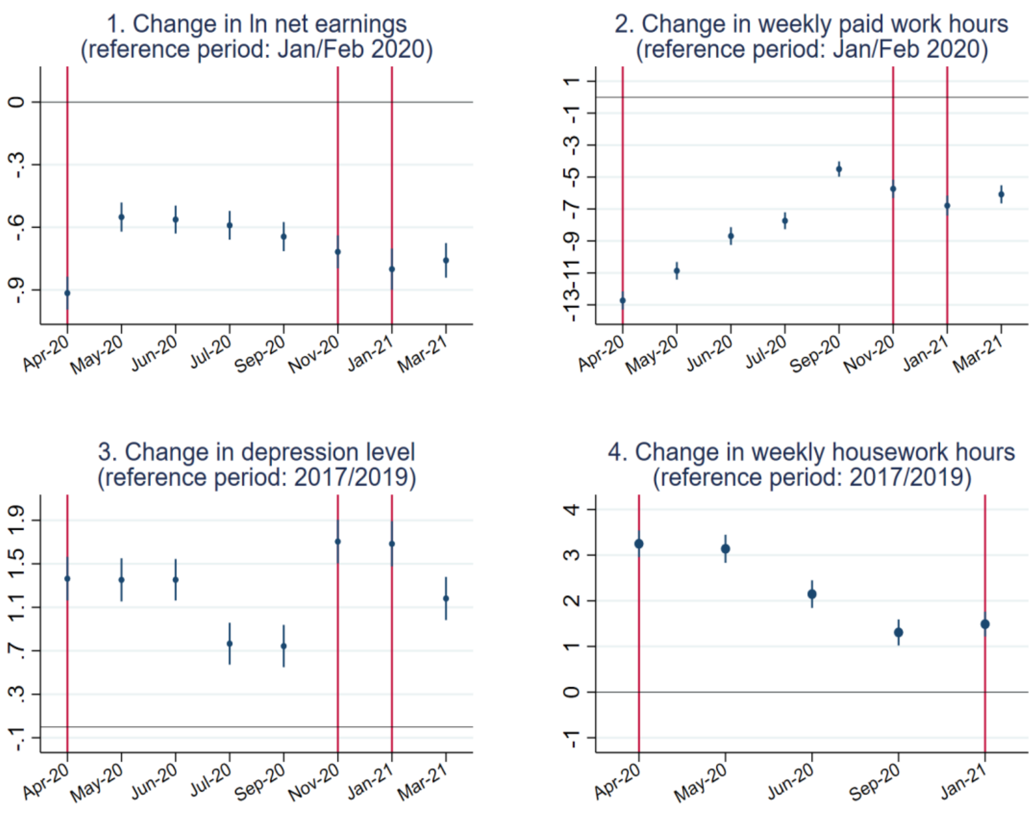

Figure 1. Point estimates and 95% confidence interval of the baseline models

One's earnings stayed lower than the pre-pandemic level over the entire year, with the largest decline (~9%) recorded in April, the first month after the announcement of the first national lockdown. Earnings recovered slightly after the gradual relaxation of the restrictive measures and the implementation of the Job Retention Scheme. Since the third lockdown, when almost the same strict measures as the first lockdown were imposed, we have found a similar level of decline in earnings (~8%) compared with the pre-pandemic period as in the first lockdown. One year after the onset of the pandemic in the UK, one still experienced a 7.6% decline in earnings compared with the pre-pandemic level.

Paid work hours remained much lower than the pre-pandemic level over the entire year. The largest drop of nearly 13 hours was observed in the first month since the March 2020 lockdown. After the first month of the first national lockdown, paid work hours bounced and had never returned to the same lowest point. People worked the longest hours in September 2020 in the past year when restrictive measures were minimal. Interestingly, despite the implementation of the second and the stricter third national lockdowns, paid work hours dropped only slightly compared to the September figure and were even higher than the July 2020 figure, even though all shops were allowed to open back in July 2020. This observation implies an increased adaptation to the work-from-home practice. After the first lockdown, more firms announced a long-term strategy to allow employees to work from home [43]. Accordingly, people have increased their paid work time even though they might still work from home.

In this pandemic, people's subjective well-being has been hurt. As can be seen, the distress level (a higher score indicating more distress) stayed higher than the pre-pandemic level over the past year. In the three-month after the first lockdown, a high level of distress was recorded. An improvement in subjective wellbeing was observed from July and before the enforcement of the second lockdown. The November lockdown brought a further decline in subjective wellbeing. This is consistent with one earlier study [16]. The distress level in November 2020 and January 2021 was even higher than that in the first lockdown period. It appears that people were much less optimistic and suffered tremendously as the pandemic dragged longer. People have become slightly less negatively affected in their subjective wellbeing in March 2021, though the level was only similar to that in April last year. One year after the onset of the pandemic in the UK, one's subjective wellbeing returned to their level back in April 2020, which was one month after the announcement of the first national lockdown.

The increase in housework hours was the highest during the first lockdown. Compared with the housework hours during the easing period in September 2020, the January 2021 lockdown was not associated with an increase people's housework time. This change echoes the relatively high level of paid work time in the latter two lockdown periods.

Next, we examine childcare time since the first national lockdown. In **Figure 2**, we can see that since in April 2020 (during the first lockdown period), childcare hours have been dropping. The lowest level was observed in September 2020, when schools completely reopened. Interestingly, childcare hours in January 2021 were similar to those in September 2020, despite the closure of schools to most children in the January 2021.

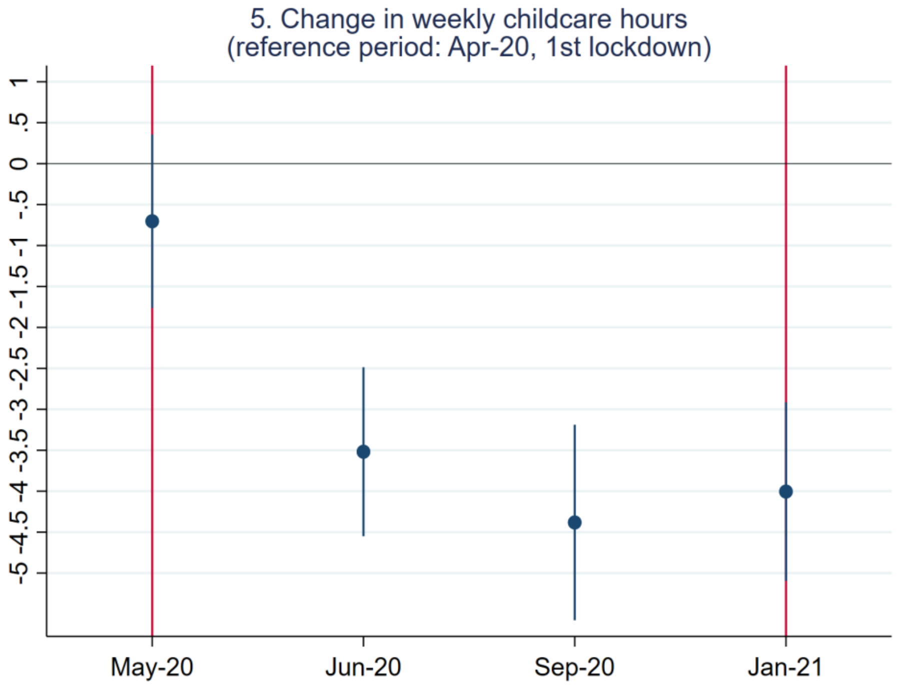

Figure 2. Point estimates and 95% confidence interval of the baseline model to predict childcare time

Differential impacts on women and men

Figures 3 and 4 report whether changes in the five indicators differ between women and men. For monthly net earnings and weekly paid work hours, we analyzed an additional sample that only includes non-keyworkers. We will examine whether the disproportional female workers in certain key sectors, such as health and social care, drive the results.

First, the drop in earnings for female workers (those who worked in Jan/Feb 2020) was smaller than male workers during the first lockdown in April 2020 (p=0.010). Since then, there has been no difference between women and men in changes in earnings, reflecting a faster recovery of men's earnings. We noted that this differential impacts on women and men were not found among non-keyworkers. Therefore, the higher level of females working in key sectors that were much more actively operating than other sectors during the first lockdown period should be the main reason for the gender difference in earning decline during the first lockdown.

During the first lockdown, the decline in paid work hours was smaller for female workers than for male workers, disregarding their keyworker status (p<0.001). The gender difference in the reduction in paid work hours had become smaller as the first lockdown ended and had become statistically insignificant at 0.05-level from July to September 2020, indicating a faster recovery of paid work time for men than women. The gender differential impacts on paid work hours observed in the first lockdown was not observed in latter lockdowns among non-keyworkers.

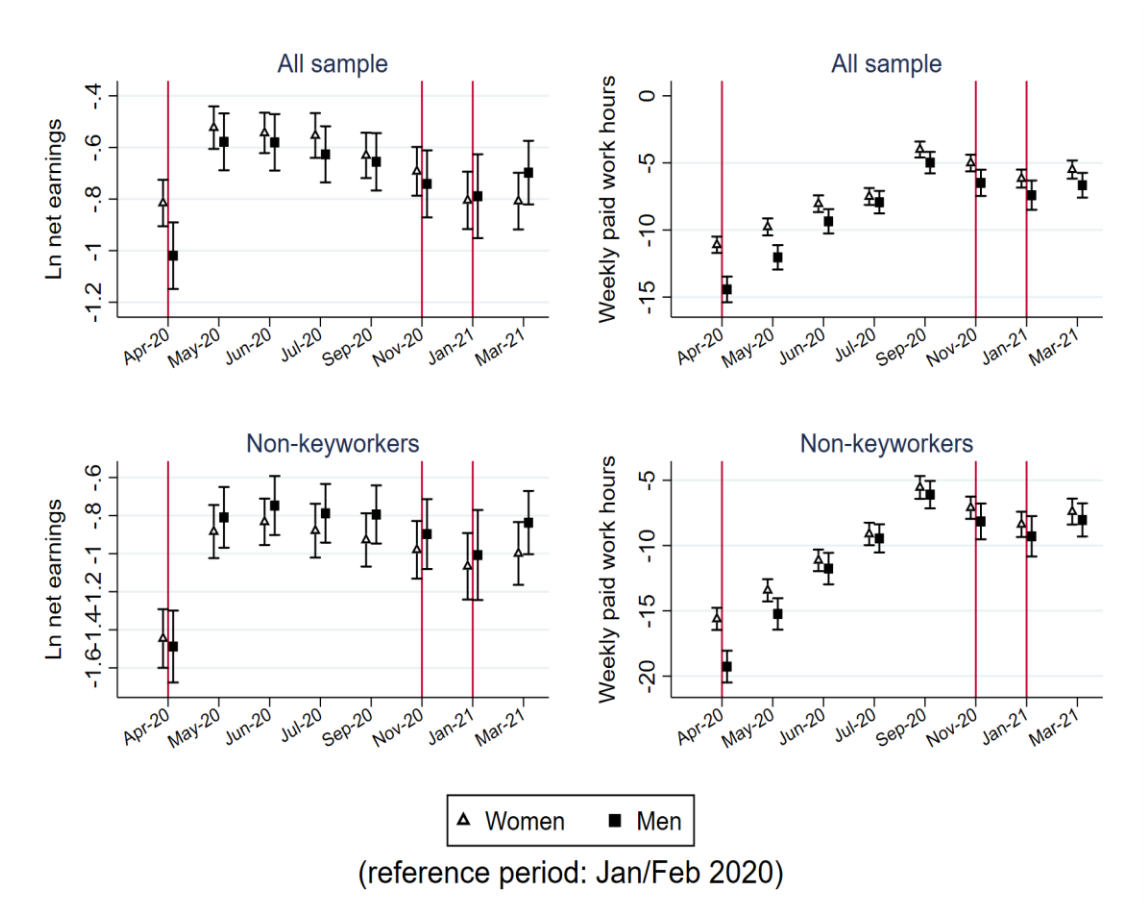

Figure 3. Point estimates and 95% confidence interval of the gender-period interaction models to predict earnings and working time

In **Figure 4**, the growth in distress level was much higher for women than for men in the first month of the first lockdown ($p<0.001$). Then, women's subjective wellbeing recovered, and men's distress levels began to rise. These findings suggest that men's response to this pandemic lagged behind women's in terms of their subjective wellbeing in the first lockdown. The distress level of both women and men decreased to a lowest level from July to September 2020, when lives in general had returned to normal. Once the lockdown restrictions were re-imposed, for example, in November 2020 ($p=0.050$) and January 2021 ($p=0.063$), women again suffered from a larger increase in distress levels than men. The distress level of women reached a similar high point across the three lockdowns. For men, their distress level was higher in the latter lockdowns than that in the first lockdown.

We do not observe a gender-specific impact on housework time. The gender gap in housework time was largely maintained over the past year.

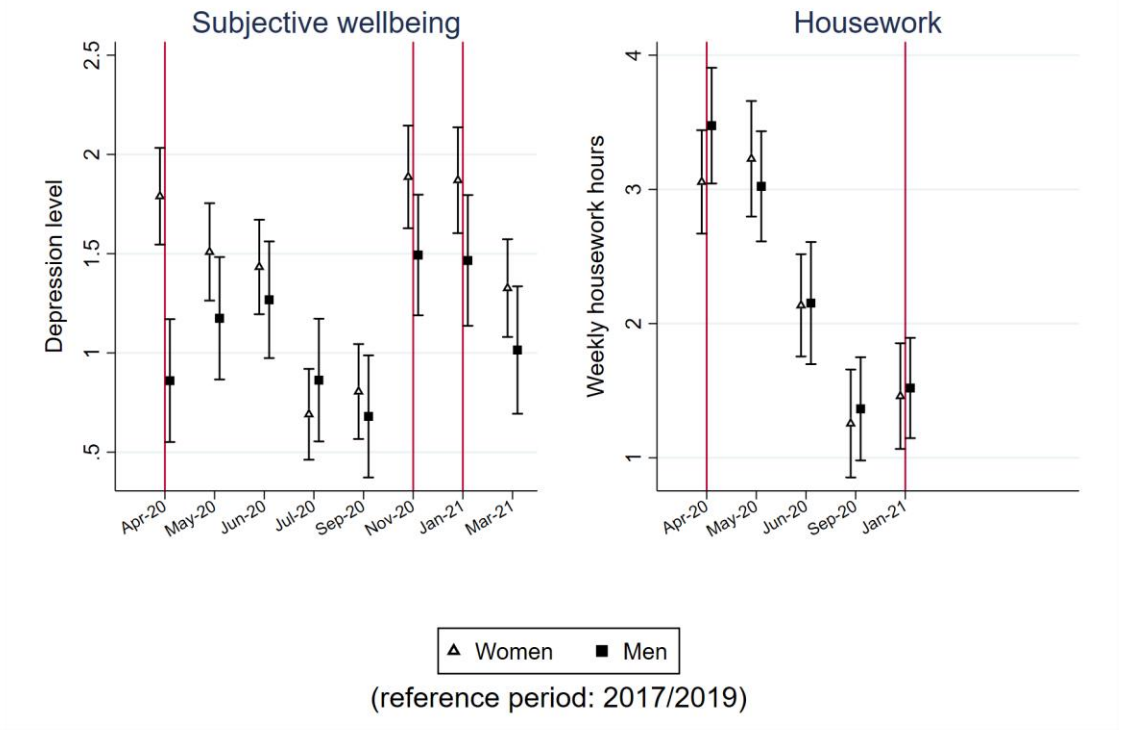

Figure 4. Point estimates and 95% confidence interval of the gender-period interaction models to predict subjective wellbeing and housework time

Differential impacts on BAME and white people

Figure 5 reports whether those changes in the five indicators differ between BAME and whites.

Compared with whites, the earnings of the BAME group were particularly negatively affected by the pandemic. And the differential impacts on earnings persisted across almost all months over the past year, except in the latter lockdowns. The gap was larg[illegible] even when most lockdown restrictions were eased in September 2020 (p=0.006). Over the past year, the decline in market working time was similar for the BAME group and whites, except in June 2020 and November 2020 lockdown, during which the paid work time of the BAME group suffered from a larger reduction (p=0.057 and p=0.067, respectively). In March 2021, the drop in paid work time became smaller for the BAME group, though the difference is not statistically signific at 0.05-level (p=0.176). The earnings and paid work time tended to be less stable for the BAME group than for the whites.

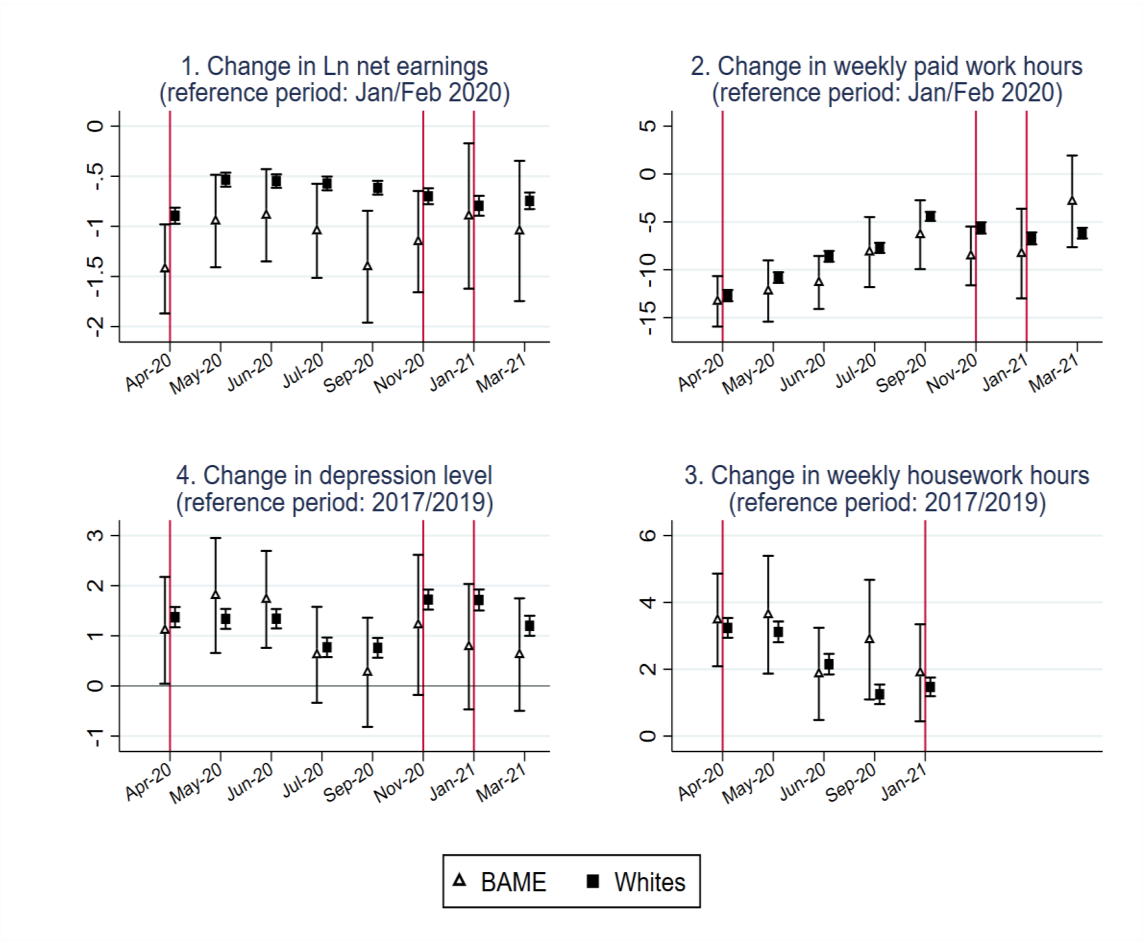

Figure 5. Point estimates and 95% confidence interval of the race-period interaction models

Regarding the distress level, the increase for the BAME group was larger than that for the whites during the first lockdown, but the difference is not statistically significant at 0.05-level due to the large standard error of the estimates of the BAME group. Between July and September 2020, the distress level of the BAME group had almost returned to the pre-pandemic level and had increased in the latter two lockdowns. In the third national lockdown, whites seemed to suffered more with a larger increase in their distress level, but the large standard error of the estimates of the BAME group prevents the difference to be statistically significant at 0.05-level. We do not observe a clear pattern showing differences in the change in housework hours between the two groups.

Differential impacts on degree and non-degree holders

Figure 6 reports whether changes in the five indicators differ between degree and non-degree holders.

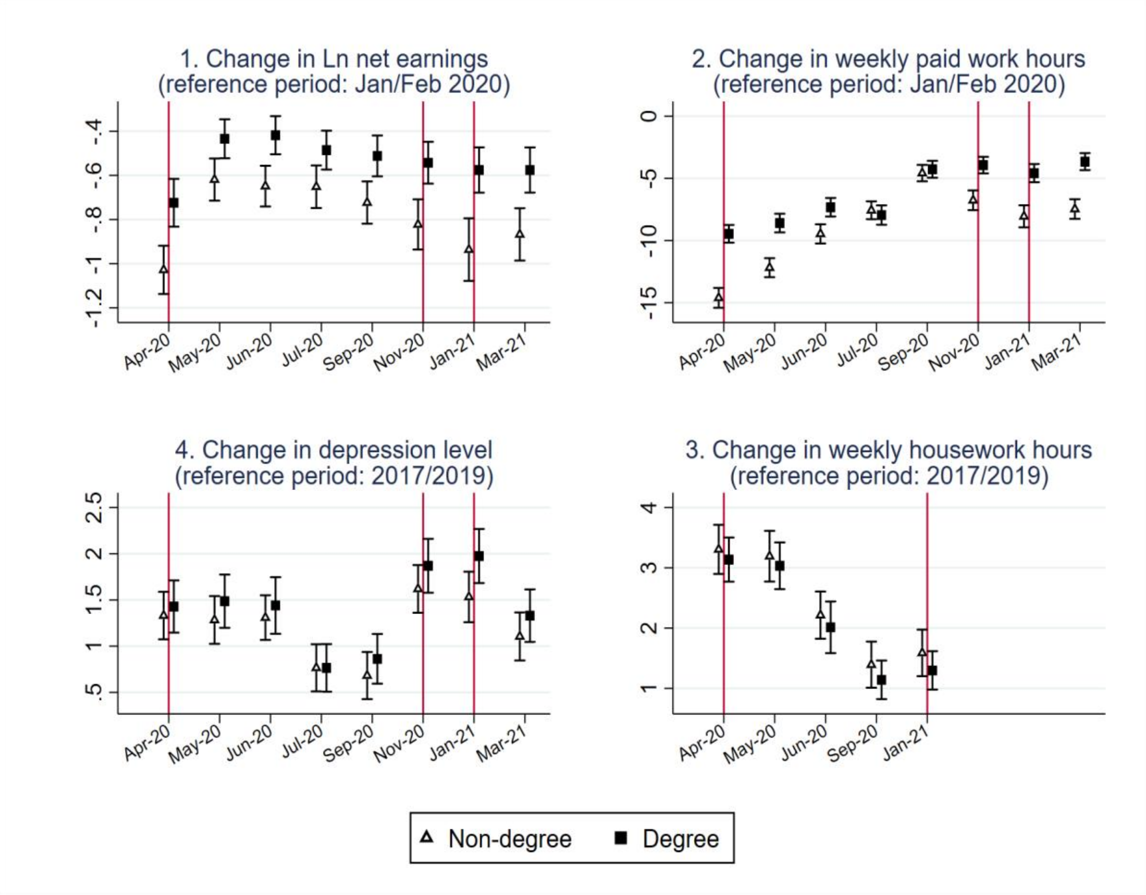

Figure 6. Point estimates and 95% confidence interval of the ~~race~~-period interaction models

As expected, the decline in earnings and paid work hours was particularly acute among non-degree holders. When most of the restrictive measures were eased from July to September 2020, the difference in the impacts on non-degree and degree holders became smaller but sustained. For paid work hours, the difference was insignificant between July and September 2020. Once lockdown restrictive measures were re-imposed, the difference has become substantial again ($p<0.001$).

There was no significant difference in the change in subjective wellbeing between degree and non-degree holders before January 2021. However, degree holders experienced a larger increase in distress level during the third national lockdown that started in January 2021 ($p=0.029$). But the differential effect disappeared in March 2021.

We do not observe a statistically significant difference in the changes in housework time between the two groups.

Changes in weekly childcare hours since April 2020

Figure 7 reports whether those changes in the weekly childcare hours differ across these groups.

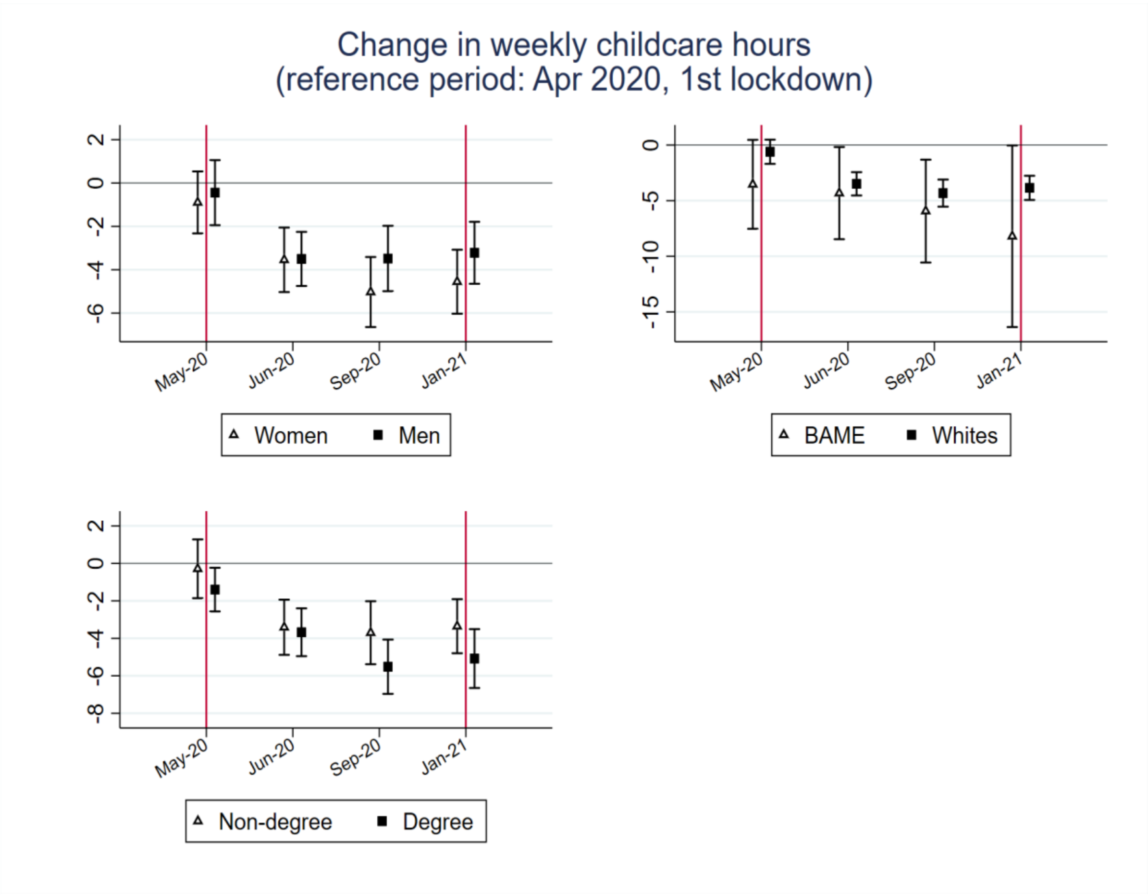

Figure 7. Point estimates and 95% confidence interval of the rac[illegible]eriod interaction models

Our findings show that women and men, BAME and whites, or degree and non-degree holders did not differ significantly in the change in their childcare time since April 2020. However, there is a tendency that the drop in childcare time in September, which should be associated with pupils' returning to schools after summer vacation, was larger for mothers and the more educated group, suggesting that women and the more educated might have spent more time to taking care of children at home.

For more details of the results, please refer to **Supporting information Tables A2 to A5**. The within-individual R-squares are small when predicting earnings, subjective wellbeing, housework time, and childcare time. The small within-individual R-squares are not uncommon in fixed-effect regressions, especially when predicting housework time and subjective wellbeing [44,45]. These results suggest that a limited numbers of individuals have changed their partnership and parenthood status, but their outcome variables—earnings, time use, and subjective wellbeing have changed a lot over the past year. The inclusion of more time-varying variables might be able to improve the explanatory power, such as whether furloughed, whether participated in the Job Retention Scheme, or whether contracted COVID-19. However, the purpose of this paper is to provide an overall net impact of the COVID-19 and its related measures on an individual instead of focusing on a specific policy or COVID-19 per se. Given the focus on the

trajectories of earnings, time use, and subjective wellbeing at different stages of the pandemic, we do not include more time-varying variables.

Discussion and Conclusion

In this article, we have utilized the latest UK COVID panel data to provide a comprehensive analysis of how the COVID-19 and the COVID-induced policies have made unequal and dynamic impacts on different social groups over the past year in the UK. Our research, with a much extended time scope, surpasses past UK studies that only followed a short period after the first lockdown imposed in March 2020 [for example, 3,6,8,19]. Our analysis has incorporated multiple domains of outcomes across several social groups. We have provided new insights into the dynamics of earnings, time-use, and subjective wellbeing at different stages of the pandemic. Our findings also provide important insights into whether the inequalities in the changes in income, time-use and well-being are likely to be long-lasting or temporary or responded similarly in each of these lockdowns.

Overall speaking, the first national lockdown brought the largest change in earnings and time use. The latter two lockdowns stroke people's subjective wellbeing the most. Although strict measures that aimed to reduce people's physical contacts were imposed in the latter two lockdowns, people's time use did not respond as strongly as they did during the first lockdown. Among the five indicators, none of them had returned to their pre-pandemic level until late March 2021. It remains uncertain as to when and whether earnings, working patterns, family life, and subjective wellbeing will return to the pre-pandemic level.

Female workers experienced less reduction in their earnings than male workers, which is largely due to the relatively high proportion of women working in key sectors, especially in the health and social care industry. Women have made an important contribution to the fight against the COVID-19 by working in key sectors. However, even among non-keyworkers, the decline in paid work hours was smaller for women, but only during the first lockdown period. These findings concur with an earlier research that reported that men in the UK were more likely to be laid off or furloughed than women during the first lockdown [19]. Once lockdown measures were gradually lifted from June 2020, men's paid work time recovered faster than women's. This finding is similar to previous work on the gendered impact of natural disasters on market labor [46]. In summary, our analysis has shown that in the UK, men's paid work time was more responsive to the first lockdown restrictive measures, but women's and men's paid work time responded similarly in latter two lockdowns.

The subjective wellbeing of women was more sensitive to the lockdown measures than that of men. For example, the increase in women's distress level was substantial within the first month of the March 2020 lockdown, but it then gradually improved until the next lockdown. Men's response lagged behind of women's. Past COVID-19 research highlighted the gender difference in social networks, where women tend to have more friends [28]. The larger exposure to news related to COVID-19 for those with more close friends might be the factor that explains the diverging trajectories of women's and men's

subjective wellbeing [47,48]. Men's lagged response was not observed in latter two lockdowns. Both women and men responded immediately with a similarly heightened level of distress. At the beginning of the pandemic, women and men seemed to have perceived the danger of this infectious disease differently.

The gender gap in housework time was largely maintained over the past year. Overall, the changes in the gender gap in earnings, paid work time, and subjective wellbeing were only observed when strict restrictions were in place, the gender gap returned to its pre-pandemic level once those measures were lifted.

People of a BAME background experienced a larger loss in earnings than whites. This finding is consistent with an earlier finding on BAME immigrants in the UK [3]. We have further shown that the enlarged earning gap between BAME and white people persisted almost over the entire year. Interestingly, the subjective wellbeing gap between BAME and whites became smaller in the latter two rounds of lockdowns. We call for future studies to investigate potential explanations.

The persistently enlarged earning gap was observed between non-degree and degree holders. Non-degree holders had suffered from a larger reduction in earnings across all months over the past year. This gap was particularly large during the national lockdown periods. A similar observation was found for the weekly paid work hours. Lockdown restrictions are associated with enlarged gap in paid work time between non-degree and degree holders. This effect is likely to be temporary because the differential impacts were not observed from July to September 2020, when lockdown measures were mostly lifted.

The increase in distress level was higher for the more educated group, but only during the period of the latter two lockdowns. This finding is consistent with one study on the US population [17]. These findings suggest that the more educated might be more sensitive to surging uncertainties brought by the development of this pandemic.

One limitation of this study is that some changes could be brought by seasonal fluctuations beyond the COVID-19 and its related restrictions. For example, people's paid work time in winter may differ from that in summer. The general psychological health was usually worse in winter than in summer [49]. The ideal solution is to compare information collected in the same month before the pandemic and in 2020. However, this approach is not possible with the current data. If the current survey retains the current monthly or bi-monthly data collection frequency, future work can compare the same month in 2020 and the years after to examine pandemic and post-pandemic differences. In summary, our results show the trajectories of earnings, time use, and subjective wellbeing at different time point over the past year but cannot identify the exact impact of a specific lockdown restrictive policy.

To conclude, our findings suggest that the long-lasting pandemic and related restriction to contain the virus over the past year have produced persistent negative consequences to earnings, work patterns, and subjective wellbeing. The national lockdowns at different stages in the UK had distinct measures, and their impacts on labor earnings, time use and subjective well-being varied. Time use patterns became less sensitive to the latter lockdowns, but the distress levels reached a new high with the repeated lockdowns. The differential impacts of the lockdown measures based on gender became insignificant once

lockdown measures were lifted. However, some social groups, including BAME and white people and the non-degree holders and degree holders, experienced persistently enlarged gaps in earnings. In conclusion, the negative impacts of the COVID-19 and its related measures vary not only by their extent but also their speed among different social groups. We need further research to understand factors that have driven these social inequalities and to monitor how the inequalities by gender, educational level, and ethnic minority status might be persistent or even exacerbated in the long term.

References

1. Davillas A, Jones AM. The COVID-19 pandemic and its impact on inequality of opportunity in psychological distress in the UK. SSRN. 2020. doi:10.2139/ssrn.3614940

2. Pierce M, Hope H, Ford T, Hatch S, Hotopf M, John A, et al. Mental health before and during the COVID-19 pandemic: A longitudinal probability sample survey of the UK population. The Lancet Psychiatry. 2020. Available: https://doi.org/10.1016/S2215-0366(20)30308-4

3. Hu Y. Intersecting ethnic and native–migrant inequalities in the economic impact of the COVID-19 pandemic in the UK. Research in Social Stratification and Mobility. 2020;68: 100528. doi:https://doi.org/10.1016/j.rssm.2020.100528

4. Patel P, Hiam L, Sowemimo A, Devakumar D, McKee M. Ethnicity and covid-19. BMJ. 2020;369. doi:10.1136/bmj.m2282

5. Craig L, Churchill B. Dual-earner parent couples' work and care during COVID-19. Gender, Work & Organization. 2021;28: 66–79. doi:https://doi.org/10.1111/gwao.12497

6. Zamberlan A, Gioachin F, Gritti D. Work less, help out more? The persistence of gender inequality in housework and childcare during UK COVID-19. Research in Social Stratification and Mobility. 2021;73: 100583. doi:https://doi.org/10.1016/j.rssm.2021.100583

7. Collins C, Landivar LC, Ruppanner L, Scarborough WJ. COVID-19 and the gender gap in work hours. Gender, Work & Organization. 2020; 1–16. doi:10.1111/gwao.12506

8. Zhou M, Hertog E, Kolpashnikova K, Kan M-Y. Gender inequalities: Changes in income, time use and well-being before and during the UK COVID-19 lockdown. SocArXiv. 2020; 1–16. doi:10.31235/osf.io/u8ytc

9. Xue A Baowen AND McMunn. Gender differences in unpaid care work and psychological distress in the UK covid-19 lockdown. PLOS ONE. 2021;16: 1–15. doi:10.1371/journal.pone.0247959

10. Leigh-Hunt N, Bagguley D, Bash K, Turner V, Turnbull S, Valtorta N, et al. An overview of systematic reviews on the public health consequences of social isolation and

loneliness. Public Health. 2011;152: 157171. doi:https://doi.org/10.1016/j.puhe.2017.07.035

11. Fitch C, Hamilton S, Bassett P, Davey R. The relationship between personal debt and mental health: A systematic review. Mental Health Review Journal. 2011;16: 153166. doi:https://doi.org/10.1108/13619321111202313

12. Kim TJ, Knesebeck O von dem. Perceived job insecurity, unemployment and depressive symptoms: A systematic review and meta‑ analysis of prospective observational studies. International Archives of Occupational and Environmental Health. 2016;89: 561573. doi:https://doi.org/10.1007/s00420-015-1107-1

13. Dawel A, Shou Y, Smithson M, Cherbuin N, Banfield M, Calear AL, et al. The effect of COVID-19 on mental health and wellbeing in a representative sample of australian adults. Frontiers in Psychiatry. 2020;11: 1026. doi:10.3389/fpsyt.2020.579985

14. Li LZ, Wang S. Prevalence and predictors of general psychiatric disorders and loneliness during COVID-19 in the united kingdom. Psychiatry Research. 2020;291: 113267. doi:https://doi.org/10.1016/j.psychres.2020.113267

15. Chandola T, Kumari M, Booker CL, Benzeval M. The mental health impact of COVID-19 and lockdown-related stressors among adults in the UK. Psychological Medicine. 2020; 1–10. doi:10.1017/S0033291720005048

16. Pierce M, McManus S, Hope H, Hotopf M, Ford T, Hatch SL, et al. Mental health responses to the COVID-19 pandemic: A latent class trajectory analysis using longitudinal UK data. The Lancet Psychiatry. 2021;8: 610–619. doi:https://doi.org/10.1016/S2215-0366(21)00151-6

17. Wanberg CR, Csillag B, Douglass RP, Zhou L, Pollard MS. Socioeconomic status and well-being during COVID-19: A resource-based examination. Journal of Applied Psychology. 2020. Available: http://dx.doi.org/10.1037/apl0000831

18. Office for National Statistics. Full report - women in the labour market: 2013. Office for National Statistics. 2013. Available: http://www.ons.gov.uk/employmentandlabourmarket/peopleinwork/employmentandemployeetypes/articles/womeninthelabourmarket/2013-09-25

19. Witteveen D. Sociodemographic inequality in exposure to COVID-19-induced economic hardship in the united kingdom. Research in Social Stratification and Mobility. 2020;69: 100551. doi:https://doi.org/10.1016/j.rssm.2020.100551

20. Andrew A, Cattan S, Dias MC, Farquharson C, Kraftman L, Krutikova S, et al. The gendered division of paid and domestic work under lockdown. Institute for Fiscal Studies Working Paper. 2020. Available: https://ifs.org.uk/uploads/WP2117-The-gendered-division-of-paid-and-domestic-work-under-lockdown.pdf

21. Fuller S, Qian Y. Covid-19 and the gender gap in employment among parents of young children in canada. Gender & Society. 2021;35: 206–217. doi:10.1177/08912432211001287

22. Collins C, Landivar LC, Ruppanner L, Scarborough WJ. COVID-19 and the gender gap in work hours. Gender, Work & Organization. 2020. doi:10.1111/gwao.12506

23. Shafer K, Milkie M, Scheibling C. How the covid-19 lockdown affected gender inequality in paid and unpaid work in spain. SocArXiv. 2020. doi:https://doi:10.31235/osf.io/24j87

24. Dominguez-Folgueras M. Difficult times: The division of domestic work under lockdown in france. Social Sciences. 2021;10. doi:10.3390/socsci10060219

25. Carlson DL, Petts R, Pepin JR. Changes in parents' domestic labor during the COVID-19 pandemic. SocArXiv. 2020. doi:https://doi:10.31235/osf.io/jy8fn

26. Czymara CS, Langenkamp A, Cano T. Cause for concerns: Gender inequality in experiencing the COVID-19 lockdown in germany. European Societies. 2021;23: S68–S81.

27. Farre L, Fawaz Y, Gonzalez L, Graves J. The division of labour before & during the COVID-19 pandemic in canada. IZA Discussion Paper. 2020.

28. Etheridge B, Spantig L. The gender gap in mental well-being during the covid-19 outbreak: Evidence from the UK. Colchester: University of Essex, Institute for Social; Economic Research (ISER); 2020. Report No.: 2020-08. Available: http://hdl.handle.net/10419/227789

29. Giovanis E. Income losses and subjective well-being: Gender and ethnic inequalities during the covid-19 lockdown period in the UK. Research Square; 2021. doi:10.21203/rs.3.rs-132600/v2

30. Perry BL, Aronson B, Pescosolido BA. Pandemic precarity: COVID-19 is exposing and exacerbating inequalities in the american heartland. Proceedings of the National Academy of Sciences. 2021;118. doi:10.1073/pnas.2020685118

31. Lemieux T, Milligan K, Schirle T, Skuterud M. Initial impacts of the COVID-19 pandemic on the canadian labour market. Canadian Public Policy. 2020;46: S55–S65. doi:10.3138/cpp.2020-049

32. Chen DT-H, Wang Y-J. Inequality-related health and social factors and their impact on well-being during the COVID-19 pandemic: Findings from a national survey in the UK. International Journal of Environmental Research and Public Health. 2021;18. doi:10.3390/ijerph18031014

33. UK G. Check if your employer can use the coronavirus job retention scheme. GOV.UK. 2020. Available: https://www.gov.uk/guidance/check-if-you-could-be-covered-by-the-coronavirus-job-retention-scheme

34. Social I for, Economic Research U of E. 2020. doi:10.5255/UKDA-SN-8644-3

35. Benzeval M, Burton J, Crossley T, Fisher P, Jöckle A, Low H, et al. The idiosyncratic impact of an aggregate shock: The distributional consequences of COVID-19. Understanding Society Working Paper Series. 2020. Available: https://www.understandingsociety.ac.uk/sites/default/files/downloads/working-papers/2020-09.pdf

36. Social I for, Economic Research. Understanding society COVID-19 user guide. Version 8.0. Colchester: University of Essex. 2021.

37. Uhrig SCN. The nature and causes of attrition in the british household panel study. Colchester: University of Essex, Institute for Social; Economic Research (ISER); 2008. Report No.: 2008-05. Available: http://hdl.handle.net/10419/92025

38. Pevalin DJ. Multiple applications of the GHQ-12 in a general population sample: An investigation of long-term retest effects. Soc Psychiatry Psychiatr Epidemiol. 2000;35. doi:10.1007/s001270050272

39. Schober PS. The Parenthood Effect on Gender Inequality: Explaining the Change in Paid and Domestic Work When British Couples Become Parents. European Sociological Review. 2011;29: 74–85. doi:10.1093/esr/jcr041

40. Zhou M, Kan M-Y. A new family equilibrium? Changing dynamics between the gender division of labor and fertility in Great Britain, 1991–2017. Demographic Research. 2019;40: 1455–1500. doi:10.4054/DemRes.2019.40.50

41. Allison PD. Fixed effects regression models. SAGE Publications; 2009. Available: https://books.google.co.uk/books?id=3UxaBQAAQBAJ

42. Abadie A, Athey S, Imbens GW, Wooldridge J. When Should You Adjust Standard Errors for Clustering? National Bureau of Economic Research, Inc; 2017 Nov. Report No.: 24003. Available: https://ideas.repec.org/p/nbr/nberwo/24003.html

43. Read S. No full-time return to the office for over a million. BBC. 2021 [cited 6 May 2021]. Available: https://www.bbc.co.uk/news/business-56972207

44. Borra C, Browning M, Sevilla Sanz A. Marriage and housework. IZA Discussion Paper. 2017. Available: https://ssrn.com/abstract=2960549

45. Foa R, Gilbert S, Fabian MO. COVID-19 and subjective well-being: Separating the effects of lockdowns from the pandemic. 2020. Available: https://ssrn.com/abstract=3674080

46. Bradshaw S. Socio-economic impacts of natural disasters: A gender analysis. United Nations Economic Commission for Latin America and the Caribbean. 2004. Available: https://www.cepal.org/en/publications/5596-socio-economic-impacts-natural-disasters-gender-analysis

47. Bu F, Steptoe A, Mak HW, Fancourt D. Time-use and mental health during the COVID-19 pandemic: A panel analysis of 55,204 adults followed across 11 weeks of lockdown in the UK. medRxiv. 2020. doi:10.1101/2020.08.18.20177345

48. Holman EA, Thompson RR, Garfin DR, Silver RC. The unfolding COVID-19 pandemic: A probability-based, nationally representative study of mental health in the united states. Science Advances. 2020;6. doi:10.1126/sciadv.abd5390

49. McLaughlin C, Bowman ML, Bradley CL, Mistlberger RE. A prospective study of seasonal variation in shift-work tolerance. Chronobiology international. 2008;25: 455470. doi:https://doi.org/10.1080/07420520802118269
