# Supplementary material for: The varying impacts of COVID-19 and its related measures in the UK: A year in review
Source: PLoS One. 2021 Sep 29;16(9):e0257286. doi: 10.1371/journal.pone.0257286 (PMC8480884; doi:10.1371/journal.pone.0257286)
Supplement: Attachment — Submitted filename: PONE-D-20-32060_R1_supplement.pdf [file pone.0257286.s011.pdf]

Supporting information

Table A1. Samples and sample selection

COVID study survey wave number	Period	Full sample (20-65 years old)	Samples with no missing value in predictors	People worked in January/February 2020 and followed in each wave	*Worker sample*: no missing value in labor income	*Worker sample*: no missing value in weekly working hours	No missing value in subjective wellbeing score	No missing value in weekly housework hours	No missing value in weekly childcare hours (asked *parents*)
1	Apr 20	13,119	12,943	10,106	9,257	10,018	11,512	11,687	5,065
2	May 20	10,649	10,532	8,410	7,695	8,360	10,253	10,117	4,129
3	Jun 20	10,048	9,943	7,883	7,114	7,833	9,618	9,505	3,742
4	Jul 20	9,739	9,630	7,608	6,773	7,565	9,347	-	-
5	Sep 20	9,060	8,966	7,050	6,266	6,998	8,591	8,515	3,163
6	Nov 20	8,392	8,317	6,541	5,728	6,490	8,033	-	-
7	Jan 2021	8,325	8,237	6,459	5,601	6,390	7,902	7,748	2,796
8	Mar 2021	8,975	8,880	6,963	6,078	6,916	8,560	-	-
Total		78,307	77,448	61,020	54,512	60,570	73,816	47,572	18,895
Number of person-years with repeated observations					52,774	58,372	70,371	48,368	14,897

Table A2. Baseline model: Changes in the five indicators across waves

UK lockdown stage	Month-Year	Ln net earnings	Weekly working hours	Subjective wellbeing	Weekly housework hours	Weekly childcare hours
		Reference period: Jan/Feb 2020		Reference period: 2018/19		Reference period: Apr 2020
1st lockdown	Apr-20	-0.915***	-12.726***	1.363***	3.248***	
		(0.040)	(0.290)	(0.102)	(0.149)	
1st lockdown	May-20	-0.551***	-10.867***	1.353***	3.138***	-0.704
		(0.036)	(0.280)	(0.101)	(0.157)	(0.540)
Easing	Jun-20	-0.563***	-8.693***	1.353***	2.146***	-3.518***
		(0.034)	(0.283)	(0.097)	(0.154)	(0.526)
Easing	Jul-20	-0.590***	-7.735***	0.765***		
		(0.035)	(0.269)	(0.098)		
Easing	Sep-20	-0.645***	-4.498***	0.744***	1.307***	-4.382***
		(0.036)	(0.244)	(0.099)	(0.145)	(0.609)
2nd lockdown	Nov-20	-0.717***	-5.736***	1.705***		
		(0.040)	(0.291)	(0.102)		
3rd lockdown	Jan-21	-0.801***	-6.788***	1.684***	1.489***	-4.005***
		(0.050)	(0.320)	(0.106)	(0.138)	(0.556)
3rd lockdown, but schools reopen	Mar-21	-0.759***	-6.082***	1.181***		
		(0.042)	(0.288)	(0.101)		
	Living with a partner	0.073	1.223*	0.251	0.151	0.506
		(0.076)	(0.581)	(0.177)	(0.308)	(1.581)
	Child<=15yrs	-0.144	-1.928*	0.082	1.087*	NA
		(0.150)	(0.916)	(0.210)	(0.452)	
	Constant	7.190***	34.544***	11.538***	8.599***	16.493***
		(0.081)	(0.557)	(0.141)	(0.249)	(1.288)
	R2	0.012	0.039	0.003	0.027	0.006
	Within R2	0.037	0.117	0.020	0.045	0.019
	Between R2	0.012	0.004	0.001	0.032	0.001
	Rho	0.614	0.589	0.613	0.627	0.658
	Number of individuals	8624	9047	11043	10946	4542
	Number of person-years	52774	58372	70371	48368	14897

Data: UKHLS main survey waves 9-10 & Understanding Society Covid survey waves 1-8.
Note: * p<0.05 ** p<0.01 *** p<0.001

Table A3. Gender and period interaction model results

	Ln net earnings	Ln net earnings - non-keyworker	Weekly working hours	Weekly working hours - non-keyworker	Subjective wellbeing	Weekly housework hours	Weekly childcare hours
Month-Year	Reference period: Jan/Feb 2020				Reference period: 2018/19		Reference period: Apr 2020
Apr-20	-1.019***	-1.488***	-14.430***	-19.272***	0.860***	3.475***	
	(0.066)	(0.096)	(0.488)	(0.621)	(0.158)	(0.220)	
May-20	-0.578***	-0.810***	-12.038***	-15.237***	1.175***	3.023***	-0.444
	(0.056)	(0.081)	(0.463)	(0.613)	(0.157)	(0.209)	(0.765)
Jun-20	-0.580***	-0.747***	-9.353***	-11.767***	1.268***	2.153***	-3.502***
	(0.056)	(0.079)	(0.462)	(0.616)	(0.150)	(0.232)	(0.636)
Jul-20	-0.627***	-0.788***	-7.927***	-9.450***	0.863***		
	(0.055)	(0.079)	(0.426)	(0.549)	(0.158)		
Sep-20	-0.656***	-0.794***	-4.977***	-6.099***	0.680***	1.364***	-3.482***
	(0.057)	(0.078)	(0.409)	(0.535)	(0.157)	(0.196)	(0.769)
Nov-20	-0.741***	-0.897***	-6.485***	-8.151***	1.493***		
	(0.066)	(0.094)	(0.498)	(0.699)	(0.155)		
Jan-21	-0.789***	-1.007***	-7.407***	-9.293***	1.466***	1.520***	-3.219***
	(0.083)	(0.120)	(0.555)	(0.788)	(0.168)	(0.191)	(0.729)
Mar-21	-0.698***	-0.837***	-6.667***	-8.039***	1.014***		
	(0.063)	(0.085)	(0.470)	(0.648)	(0.164)		
Women # Apr-20	0.204*	0.042	3.326***	3.653***	0.929***	-0.419	
	(0.080)	(0.124)	(0.576)	(0.742)	(0.194)	(0.290)	
Women # May-20	0.055	-0.075	2.268***	1.808*	0.334	0.205	-0.451
	(0.068)	(0.105)	(0.558)	(0.749)	(0.196)	(0.295)	(1.037)
Women # Jun-20	0.037	-0.086	1.311*	0.633	0.165	-0.017	-0.041
	(0.068)	(0.098)	(0.545)	(0.724)	(0.188)	(0.292)	(0.967)
Women # Jul-20	0.073	-0.091	0.424	0.338	-0.172		
	(0.071)	(0.107)	(0.521)	(0.684)	(0.191)		
Women # Sep-20	0.025	-0.134	0.977	0.550	0.125	-0.109	-1.547
	(0.072)	(0.106)	(0.520)	(0.713)	(0.195)	(0.280)	(1.060)
Women # Nov-20	0.049	-0.082	1.477*	1.049	0.393*		
	(0.082)	(0.122)	(0.586)	(0.822)	(0.200)		
Women # Jan-21	-0.016	-0.059	1.239	0.915	0.404	-0.060	-1.335
	(0.098)	(0.147)	(0.646)	(0.915)	(0.217)	(0.280)	(1.007)
Women # Mar-21	-0.110	-0.162	1.170*	0.642	0.312		
	(0.083)	(0.118)	(0.579)	(0.820)	(0.206)		
Constant	7.187***	7.217***	34.506***	34.013***	11.530***	8.600***	16.401***
	(0.080)	(0.118)	(0.554)	(0.684)	(0.142)	(0.248)	(1.282)
R2	0.011	0.023	0.027	0.057	0.007	0.025	0.003
Within R2	0.037	0.062	0.119	0.192	0.022	0.045	0.020
Between R2	0.008	0.034	0.004	0.000	0.001	0.027	0.000

	Ln net earnings	Ln net earnings - non-keyworker	Weekly working hours	Weekly working hours - non-keyworker	Subjective wellbeing	Weekly housework hours	Weekly childcare hours
Month-Year	Reference period: Jan/Feb 2020				Reference period: 2018/19		Reference period: Apr 2020
Rho	0.615	0.611	0.596	0.593	0.612	0.627	0.659
Number of individuals	8624	5340	9047	5631	11043	10946	4542
Number of person-years	52774	30917	58372	34401	70371	48368	14897

Data: UKHLS & Understanding Society Covid survey waves 1-8. Models include whether they live with a partner and whether there is a child younger than 16 years old in the household.
Note: * $p<0.05$ ** $p<0.01$ *** $p<0.001$

Table A4. Ethnicity and period interaction models

	Ln net earnings	Weekly working hours	Subjective wellbeing	Weekly housework hours	Weekly childcare hours
Month-Year	Reference period: Jan/Feb 2020		Reference period: 2018/19		Reference period: Apr 2020
Apr-20	-0.894***	-12.698***	1.373***	3.237***	
	(0.041)	(0.299)	(0.103)	(0.151)	
May-20	-0.534***	-10.815***	1.338***	3.119***	-0.606
	(0.036)	(0.285)	(0.101)	(0.158)	(0.554)
Jun-20	-0.549***	-8.600***	1.341***	2.151***	-3.488***
	(0.034)	(0.288)	(0.098)	(0.158)	(0.534)
Jul-20	-0.572***	-7.714***	0.771***		
	(0.035)	(0.271)	(0.099)		
Sep-20	-0.615***	-4.427***	0.761***	1.250***	-4.323***
	(0.035)	(0.242)	(0.100)	(0.148)	(0.621)
Nov-20	-0.700***	-5.631***	1.723***		
	(0.040)	(0.297)	(0.102)		
Jan-21	-0.795***	-6.729***	1.715***	1.472***	-3.850***
	(0.051)	(0.319)	(0.106)	(0.142)	(0.553)
Mar-21	-0.746***	-6.182***	1.201***		
	(0.042)	(0.285)	(0.102)		
BAME=1 # Apr-20	-0.531*	-0.598	-0.263	0.238	
	(0.231)	(1.387)	(0.549)	(0.716)	
BAME=1 # May-20	-0.413	-1.404	0.466	0.512	-2.928
	(0.237)	(1.656)	(0.588)	(0.906)	(2.113)
BAME=1 # Jun-20	-0.341	-2.726	0.385	-0.291	-0.835
	(0.237)	(1.434)	(0.499)	(0.720)	(2.133)
BAME=1 # Jul-20	-0.473	-0.440	-0.150		
	(0.242)	(1.885)	(0.494)		
BAME=1 # Sep-20	-0.788**	-1.911	-0.488	1.635	-1.615
	(0.286)	(1.842)	(0.562)	(0.936)	(2.405)
BAME=1 # Nov-20	-0.453	-2.924	-0.505		
	(0.261)	(1.593)	(0.716)		
BAME=1 # Jan-21	-0.102	-1.579	-0.932	0.419	-4.351
	(0.374)	(2.399)	(0.642)	(0.763)	(4.185)
BAME=1 # Mar-21	-0.300	3.322	-0.575		
	(0.360)	(2.455)	(0.579)		
Constant	7.186***	34.522***	11.534***	8.602***	16.458***
	(0.081)	(0.559)	(0.141)	(0.249)	(1.288)
R2	0.012	0.039	0.003	0.027	0.007
Within R2	0.038	0.118	0.021	0.045	0.020
Between R2	0.011	0.004	0.001	0.031	0.002
Rho	0.614	0.590	0.613	0.627	0.658
Number of individuals	8624	9047	11043	10946	4542
Number of person-years	52774	58372	70371	48368	14897

Data: UKHLS & Understanding Society Covid survey waves 1-8. Models include whether they live with a partner and whether there is a child younger than 16 years old in the household. Note: * p<0.05 ** p<0.01 *** p<0.001

Table A5. Education and period interaction model results

Month-Year	Ln net earnings	Weekly working hours	Subjective wellbeing	Weekly housework hours	Weekly childcare hours
	Reference period: Jan/Feb 2020		Reference period: 2018/19		Reference period: Apr 2020
Apr-20	-1.028***	-14.609***	1.331***	3.305***	
	(0.056)	(0.406)	(0.131)	(0.208)	
May-20	-0.619***	-12.184***	1.284***	3.193***	-0.290
	(0.049)	(0.389)	(0.132)	(0.215)	(0.800)
Jun-20	-0.649***	-9.466***	1.309***	2.215***	-3.410***
	(0.047)	(0.393)	(0.123)	(0.200)	(0.750)
Jul-20	-0.651***	-7.570***	0.766***		
	(0.049)	(0.365)	(0.130)		
Sep-20	-0.723***	-4.577***	0.682***	1.393***	-3.702***
	(0.049)	(0.336)	(0.130)	(0.195)	(0.857)
Nov-20	-0.822***	-6.765***	1.619***		
	(0.058)	(0.404)	(0.132)		
Jan-21	-0.936***	-8.054***	1.533***	1.589***	-3.354***
	(0.072)	(0.452)	(0.139)	(0.197)	(0.736)
Mar-21	-0.868***	-7.468***	1.105***		
	(0.060)	(0.402)	(0.132)		
Has 1st degree # Apr-20	0.304***	5.149***	0.098	-0.169	
	(0.079)	(0.551)	(0.188)	(0.283)	
Has 1st degree # May-20	0.185**	3.589***	0.203	-0.159	-1.111
	(0.064)	(0.548)	(0.195)	(0.290)	(1.019)
Has 1st degree # Jun-20	0.231***	2.141***	0.131	-0.203	-0.266
	(0.064)	(0.549)	(0.198)	(0.289)	(1.004)
Has 1st degree # Jul-20	0.166*	-0.385	-0.001		
	(0.067)	(0.551)	(0.182)		
Has 1st degree # Sep-20	0.212**	0.306	0.181	-0.251	-1.816
	(0.067)	(0.494)	(0.185)	(0.240)	(1.118)
Has 1st degree # Nov-20	0.280***	2.829***	0.250		
	(0.077)	(0.516)	(0.195)		
Has 1st degree # Jan-21	0.361***	3.472***	0.443*	-0.290	-1.726
	(0.087)	(0.586)	(0.202)	(0.260)	(1.074)
Has 1st degree # Mar-21	0.292***	3.810***	0.225		
	(0.080)	(0.528)	(0.195)		
Constant	7.194***	34.580***	11.542***	8.598***	16.514***
	(0.081)	(0.559)	(0.141)	(0.249)	(1.288)
R2	0.022	0.050	0.003	0.028	0.003
Within R2	0.038	0.124	0.021	0.045	0.020
Between R2	0.032	0.019	0.001	0.034	0.000
Rho	0.612	0.587	0.613	0.626	0.659
Number of individuals	8624	9047	11043	10946	4542
Number of person-years	52774	58372	70371	48368	14897

Data: UKHLS & Understanding Society Covid survey waves 1-8. Models include whether they live with a partner and whether there is a child younger than 16 years old in the household. Note: * p<0.05 ** p<0.01 *** p<0.001
