# Supplementary material for: The varying impacts of COVID-19 and its related measures in the UK: A year in review
Source: PLoS One. 2021 Sep 29;16(9):e0257286. doi: 10.1371/journal.pone.0257286 (PMC8480884; doi:10.1371/journal.pone.0257286)
Supplement: Attachment — Submitted filename: 2021 PLOS1 32060R1 report.pdf [file pone.0257286.s012.pdf]

Report on: The varying impacts of the three COVID-19 lockdowns: A year in review (MS# PONE-D-20-32060R1)

As the title suggests, the paper quantifies changes in well-being for a national sample during April 2020-March 2021 when COVID-19 and related policy measures had major impacts on United Kingdom residents. Across all measures, the paper finds worse outcomes during lockdown periods (April 2020, November 2020 and January 2021) compared to non-lockdown periods, which are concentrated among women (except earnings), Black, Asian and Minority ethnic (BAME) immigrants (except depression), and non-degree holders. The Abstract concludes "the extent of the impacts of the COVID-19 and the COVID-induced measures as well as the speed at which these effects develop, vary across social groups with different types of vulnerabilities."

The paper uses good methods to document how income and behavior changed over the last twelve months but is less convincing in attributing *ALL* the changes to the Covid-19 pandemic and subsequent lockdown measures. In other words, the paper documents correlation but not causation. It seems implausible that all policy measures would have instantaneous effects as currently assumed, or that individuals would not have changed their behavior voluntarily even absent government mandates. Put more starkly, the key driving variable is likely COVID infection rates (and death rates) rather than lockdown policies, which are a lagged response reflecting common causation. The paper should plot the national COVID-19 infection and death rates for the same months as the surveys (and include as an explanatory variable in regressions) to test if these rates explain outcomes better than the lockdown policies. The marked-up paper identifies minor editorial suggestions. More detailed comments follow:

1. The longer sample period suggests more reliable inferences.
2. The paper says on p. 8, "clustered standard errors are used to consider the sampling design of the UKHLS survey" but provides no further information, even in the Supplement. Without these details, it is difficult to assess whether the statistical significance is overstated, although the pattern of mean effects is quite convincing.
3. The paper controls for keyworker status to understand the greater decline of male earnings and hours worked during the first lockdown period. A parallel analysis should have been conducted in the ethnicity and education level comparisons, since BAME and less-educated people may have been more likely to among keyworkers.
4. The paper finds that overall wellbeing was worse during lockdown periods than non-lockdown periods but does not try to distinguish between the effects of COVID-19 and the lockdown measures. Clearly COVID-19 infection and death rates and lockdown

policies have bidirectional causation: higher infection/death rates induce lockdown policies, which then (temporarily) reduce them. Does controlling for the monthly COVID-19 infection and death rates improve explanatory power in the models?

5. The paper should be more cautious in attributing the results to COVID-19 (and possibly seasonal affective disorder). Given that there may have been important policy changes beyond those related to COVID-19, as well as voluntary changes in behavior, all the paper reports is the net effect of all changes. For example, in the U.S., Blanchflower and Bryson (2021 NBER) examine mental health scores from April 2020 through June 2021, and find that anxiety, depression and worry peaked in November 2020, coinciding with the U.S. Presidential Election. Without searching for events that might have changed the national mood (e.g., Brexit fallout) other than COVID-19 and related policies, I think that the paper might be over-attributing changes to COVID-19.
6. The paper should be sent out for professional proof-reading before resubmission.
