# Supplementary material for: The varying impacts of COVID-19 and its related measures in the UK: A year in review
Source: PLoS One. 2021 Sep 29;16(9):e0257286. doi: 10.1371/journal.pone.0257286 (PMC8480884; doi:10.1371/journal.pone.0257286)
Supplement: Attachment — Submitted filename: Supplementary_PlosOne.docx [file pone.0257286.s007.docx]

# Supplementary

Table 1A. Samples and sample selection

| Wave number | Period | Full sample | Samples with no missing value in predictors | Sample with no missing value in working hour change | Sample with no missing value in housework hours change | Samples with no missing value in subjective wellbeing change | People worked in previous wave | Worker sample with no missing value in labour income change | Parent sample | Parent sample with no missing value in childcare time change |
| --- | --- | --- | --- | --- | --- | --- | --- | --- | --- | --- |
| Wave 29 | April | 12173 | 9497 | 9167 | 8496 | 8634 | 7379 | 6325 | 3504 | NA |
| Wave 30 | May | 10231 | 8126 | 7976 | 7826 | 7930 | 6266 | 5215 | 2791 | 2374 |
| Wave 31 | June | 9317 | 7496 | 7405 | 7216 | 7296 | 5745 | 4948 | 2438 | 2241 |

Table A2. Change in monthly labour earnings

|  | W29 OLS | W29 Heckman | W30 OLS | W30 Heckman | W31 OLS | W31 Heckman |
| --- | --- | --- | --- | --- | --- | --- |
| Childless women (ref: childless men) | 0.019 | 0.015 | -0.037 | -0.021 | -0.034 | -0.014 |
|  | (0.017) | (0.011) | (0.025) | (0.014) | (0.018) | (0.014) |
| Father | -0.040 | -0.024 | -0.061^*^ | -0.027 | -0.013 | -0.015 |
|  | (0.028) | (0.015) | (0.026) | (0.016) | (0.023) | (0.016) |
| Mother | -0.004 | 0.005 | -0.042 | -0.022 | -0.002 | 0.007 |
|  | (0.022) | (0.014) | (0.027) | (0.015) | (0.025) | (0.016) |
| Couple | 0.032 | 0.011 | -0.010 | 0.002 | -0.020 | -0.003 |
|  | (0.021) | (0.012) | (0.029) | (0.013) | (0.020) | (0.014) |
| BAME | -0.089^*^ | -0.059^***^ | -0.066^*^ | -0.008 | -0.019 | 0.002 |
|  | (0.038) | (0.017) | (0.031) | (0.018) | (0.024) | (0.020) |
| Higher second.(Ref: GCSE) | 0.046^*^ | 0.025 | -0.018 | -0.008 | 0.002 | 0.020 |
|  | (0.023) | (0.013) | (0.028) | (0.015) | (0.018) | (0.015) |
| University or above | 0.071^**^ | 0.040^**^ | -0.028 | -0.031^*^ | -0.003 | 0.021 |
|  | (0.022) | (0.014) | (0.028) | (0.015) | (0.017) | (0.016) |
| Age centered | 0.002^*^ | 0.000 | -0.001 | -0.001 | 0.000 | 0.001 |
|  | (0.001) | (0.000) | (0.001) | (0.001) | (0.001) | (0.001) |
| Age centered squared | -0.013 | -0.015^***^ | -0.006 | 0.009^*^ | 0.001 | -0.000 |
|  | (0.007) | (0.004) | (0.008) | (0.005) | (0.006) | (0.005) |
| Constant | -0.134^***^ | -0.107^***^ | 0.127^*^ | 0.126^***^ | 0.050 | 0.014 |
|  | (0.030) | (0.027) | (0.063) | (0.026) | (0.029) | (0.031) |
| Childless women (ref: childless men) |  | 0.229^***^ |  | 0.257^***^ |  | 0.245^***^ |
|  |  | (0.024) |  | (0.025) |  | (0.025) |
| Father |  | -0.144^***^ |  | -0.166^***^ |  | -0.237^***^ |
|  |  | (0.037) |  | (0.039) |  | (0.040) |
| Mother |  | -0.034 |  | -0.052 |  | -0.075^*^ |
|  |  | (0.034) |  | (0.036) |  | (0.037) |
| Couple |  | 0.539^***^ |  | 0.462^***^ |  | 0.487^***^ |
|  |  | (0.029) |  | (0.030) |  | (0.031) |
| BAME |  | -0.689^***^ |  | -0.701^***^ |  | -0.760^***^ |
|  |  | (0.037) |  | (0.039) |  | (0.041) |
| Born in the UK |  | 0.433^***^ |  | 0.431^***^ |  | 0.413^***^ |
|  |  | (0.036) |  | (0.038) |  | (0.039) |
| Higher second.(Ref: GCSE) |  | 0.307^***^ |  | 0.290^***^ |  | 0.318^***^ |
|  |  | (0.027) |  | (0.029) |  | (0.029) |
| University or above |  | 0.688^***^ |  | 0.711^***^ |  | 0.711^***^ |
|  |  | (0.028) |  | (0.030) |  | (0.031) |
| Ln household net income |  | -0.246^***^ |  | -0.269^***^ |  | -0.288^***^ |
|  |  | (0.011) |  | (0.013) |  | (0.013) |
| Age centered |  | -0.003^**^ |  | 0.000 |  | -0.000 |
|  |  | (0.001) |  | (0.001) |  | (0.001) |
| Age centered squared |  | -0.140^***^ |  | -0.161^***^ |  | -0.164^***^ |
|  |  | (0.009) |  | (0.010) |  | (0.010) |
| Owned mortgage (ref: Owned outright) |  | -0.015 |  | 0.000 |  | -0.013 |
|  |  | (0.032) |  | (0.033) |  | (0.034) |
| Rent-public |  | -0.579^***^ |  | -0.607^***^ |  | -0.625^***^ |
|  |  | (0.043) |  | (0.046) |  | (0.047) |
| Rent-private |  | -0.412^***^ |  | -0.401^***^ |  | -0.432^***^ |
|  |  | (0.043) |  | (0.046) |  | (0.047) |
| North (ref:London) |  | -0.179^***^ |  | -0.174^***^ |  | -0.152^**^ |
|  |  | (0.048) |  | (0.052) |  | (0.053) |
| Yorkshire & Humber |  | -0.157^**^ |  | -0.161^**^ |  | -0.146^*^ |
|  |  | (0.054) |  | (0.058) |  | (0.059) |
| Midlands |  | -0.098^*^ |  | -0.076 |  | -0.051 |
|  |  | (0.047) |  | (0.050) |  | (0.051) |
| East |  | 0.078 |  | 0.129^*^ |  | 0.148^*^ |
|  |  | (0.054) |  | (0.058) |  | (0.060) |
| South |  | 0.006 |  | 0.015 |  | 0.053 |
|  |  | (0.046) |  | (0.049) |  | (0.050) |
| Wales/Scot/NI |  | -0.153^**^ |  | -0.162^**^ |  | -0.148^**^ |
|  |  | (0.047) |  | (0.050) |  | (0.051) |
| month# till Covid study |  | -0.049^***^ |  | -0.047^***^ |  | -0.046^***^ |
|  |  | (0.001) |  | (0.001) |  | (0.001) |
| Constant |  | 2.350^***^ |  | 2.421^***^ |  | 2.520^***^ |
|  |  | (0.112) |  | (0.123) |  | (0.125) |
| athrho |  | 0.046 |  | -0.142^***^ |  | 0.001 |
|  |  | (0.047) |  | (0.035) |  | (0.056) |
| lnsigma |  | -1.084^***^ |  | -1.035^***^ |  | -1.073^***^ |
|  |  | (0.023) |  | (0.029) |  | (0.031) |
| R2 | 0.026 |  | 0.008 |  | 0.003 |  |
| Lambda |  | 0.016 |  | -0.050 |  | 0.000 |
| Rho |  | 0.046 |  | -0.141 |  | 0.001 |
| Chi-squared model test |  | 29.635 |  | 19.574 |  | 8.122 |
| P-value model test | 0.000 | 0.001 | 0.286 | 0.021 | 0.592 | 0.522 |
| Chi-squared comparison test |  | 0.967 |  | 16.686 |  | 0.000 |
| P-value comparison test |  | 0.325 |  | 0.000 |  | 0.990 |
| Number of iterations |  | 2 |  | 2 |  | 1 |
| Whether converged |  | 1 |  | 1 |  | 1 |
| Number of individuals | 6325 | 40981 | 5215 | 39871 | 4948 | 39604 |

Data: UKHLS Understanding Society wave 8-9 and Covid waves 1-3. Note: * p<0.05 ** p<0.01 *** p<0.001

Table A3. Change in weekly paid work hours

|  | W29 OLS | W29 Heckman | W30 OLS | W30 Heckman | W31 OLS | W31 Heckman |
| --- | --- | --- | --- | --- | --- | --- |
| Childless women (ref: childless men) | 2.946^***^ | 2.276^***^ | -0.054 | -0.195 | -0.609 | -0.625 |
|  | (0.735) | (0.450) | (0.693) | (0.341) | (0.490) | (0.347) |
| Father | -1.735 | -0.775 | -0.358 | -0.216 | 0.557 | 0.401 |
|  | (1.112) | (0.691) | (1.159) | (0.596) | (0.892) | (0.577) |
| Mother | 3.915^***^ | 3.448^***^ | -0.259 | -0.469 | -1.365^*^ | -0.581 |
|  | (0.834) | (0.554) | (0.790) | (0.447) | (0.613) | (0.466) |
| Couple | -0.578 | -0.363 | 0.796 | 0.280 | 0.395 | 0.344 |
|  | (0.693) | (0.447) | (0.577) | (0.327) | (0.461) | (0.332) |
| BAME | 3.144^**^ | 1.997^***^ | -2.574^**^ | -0.952^*^ | -0.023 | -0.653 |
|  | (0.999) | (0.526) | (0.822) | (0.432) | (0.770) | (0.391) |
| Higher second.(Ref: GCSE) | 0.556 | 0.705 | -0.860 | 0.231 | -0.089 | 0.215 |
|  | (0.769) | (0.469) | (0.897) | (0.379) | (0.521) | (0.361) |
| University or above | 3.525^***^ | 3.290^***^ | -1.490^*^ | -0.867^*^ | -1.069^*^ | -0.807^*^ |
|  | (0.722) | (0.454) | (0.742) | (0.361) | (0.522) | (0.352) |
| Age centered | 0.138^***^ | 0.097^***^ | -0.039 | -0.013 | -0.053^**^ | -0.032^*^ |
|  | (0.028) | (0.017) | (0.029) | (0.014) | (0.018) | (0.014) |
| Age centered squared | 0.166 | 0.361^*^ | 0.231 | -0.001 | -0.092 | -0.064 |
|  | (0.229) | (0.144) | (0.256) | (0.115) | (0.154) | (0.113) |
| Constant | -12.819^***^ | -11.224^***^ | 0.656 | 1.282^*^ | 2.585^***^ | 2.031^***^ |
|  | (1.066) | (0.815) | (1.106) | (0.588) | (0.716) | (0.565) |
| Childless women (ref: childless men) |  | 0.246^***^ |  | 0.279^***^ |  | 0.274^***^ |
|  |  | (0.021) |  | (0.022) |  | (0.022) |
| Father |  | -0.148^***^ |  | -0.179^***^ |  | -0.247^***^ |
|  |  | (0.034) |  | (0.036) |  | (0.037) |
| Mother |  | 0.066^*^ |  | 0.054 |  | 0.018 |
|  |  | (0.031) |  | (0.033) |  | (0.034) |
| Couple |  | 0.541^***^ |  | 0.453^***^ |  | 0.477^***^ |
|  |  | (0.026) |  | (0.027) |  | (0.028) |
| BAME |  | -0.622^***^ |  | -0.642^***^ |  | -0.677^***^ |
|  |  | (0.034) |  | (0.036) |  | (0.037) |
| Born in the UK |  | 0.427^***^ |  | 0.432^***^ |  | 0.424^***^ |
|  |  | (0.032) |  | (0.034) |  | (0.035) |
| Higher second.(Ref: GCSE) |  | 0.295^***^ |  | 0.287^***^ |  | 0.301^***^ |
|  |  | (0.024) |  | (0.025) |  | (0.026) |
| University or above |  | 0.632^***^ |  | 0.650^***^ |  | 0.657^***^ |
|  |  | (0.026) |  | (0.027) |  | (0.028) |
| Ln household net income |  | -0.290^***^ |  | -0.313^***^ |  | -0.320^***^ |
|  |  | (0.010) |  | (0.011) |  | (0.012) |
| Age centered |  | 0.001 |  | 0.005^***^ |  | 0.005^***^ |
|  |  | (0.001) |  | (0.001) |  | (0.001) |
| Age centred squared |  | -0.106^***^ |  | -0.124^***^ |  | -0.124^***^ |
|  |  | (0.008) |  | (0.009) |  | (0.009) |
| Owned mortgage (ref: Owned outright) |  | -0.100^***^ |  | -0.090^**^ |  | -0.106^***^ |
|  |  | (0.030) |  | (0.031) |  | (0.031) |
| Rent-public |  | -0.581^***^ |  | -0.619^***^ |  | -0.637^***^ |
|  |  | (0.038) |  | (0.040) |  | (0.041) |
| Rent-private |  | -0.498^***^ |  | -0.506^***^ |  | -0.527^***^ |
|  |  | (0.040) |  | (0.043) |  | (0.043) |
| North (ref:London) |  | -0.158^***^ |  | -0.186^***^ |  | -0.169^***^ |
|  |  | (0.045) |  | (0.048) |  | (0.049) |
| Yorkshire & Humber |  | -0.153^**^ |  | -0.170^**^ |  | -0.154^**^ |
|  |  | (0.049) |  | (0.053) |  | (0.054) |
| Midlands |  | -0.069 |  | -0.078 |  | -0.058 |
|  |  | (0.043) |  | (0.046) |  | (0.047) |
| East |  | 0.085 |  | 0.094 |  | 0.116^*^ |
|  |  | (0.050) |  | (0.053) |  | (0.055) |
| South |  | 0.017 |  | 0.008 |  | 0.040 |
|  |  | (0.042) |  | (0.045) |  | (0.046) |
| Wales/Scot/NI |  | -0.130^**^ |  | -0.169^***^ |  | -0.144^**^ |
|  |  | (0.043) |  | (0.046) |  | (0.047) |
| month# till Covid study |  | -0.051^***^ |  | -0.049^***^ |  | -0.048^***^ |
|  |  | (0.001) |  | (0.001) |  | (0.001) |
| Constant |  | 2.933^***^ |  | 3.050^***^ |  | 3.035^***^ |
|  |  | (0.104) |  | (0.113) |  | (0.117) |
| athrho |  | -0.121^***^ |  | -0.063^**^ |  | 0.023 |
|  |  | (0.027) |  | (0.023) |  | (0.023) |
| lnsigma |  | 2.784^***^ |  | 2.458^***^ |  | 2.430^***^ |
|  |  | (0.009) |  | (0.017) |  | (0.020) |
| R2 | 0.032 |  | 0.008 |  | 0.006 |  |
| Lambda |  | -1.955 |  | -0.740 |  | 0.260 |
| Rho |  | -0.121 |  | -0.063 |  | 0.023 |
| Chi-squared model test |  | 169.636 |  | 22.436 |  | 26.397 |
| P-value model test | 0.000 | 0.000 | 0.013 | 0.008 | 0.040 | 0.002 |
| Chi-squared comparison test |  | 20.834 |  | 7.315 |  | 0.971 |
| P-value comparison test |  | 0.000 |  | 0.007 |  | 0.325 |
| Number of iterations |  | 2 |  | 2 |  | 2 |
| Whether converged |  | 1 |  | 1 |  | 1 |
| Number of individuals | 9167 | 43823 | 7976 | 42632 | 7405 | 42061 |

Data: UKHLS Understanding Society wave 8-9 and Covid waves 1-3. Note: * p<0.05 ** p<0.01 *** p<0.001

Table A4. Change in weekly housework time

|  | W29 OLS | W29 Heckman | W30 OLS | W30 Heckman | W31 OLS | W31 Heckman |
| --- | --- | --- | --- | --- | --- | --- |
| Childless women (ref: childless men) | -0.310 | -0.115 | -0.235 | 0.181 | 0.088 | -0.061 |
|  | (0.403) | (0.254) | (0.440) | (0.244) | (0.440) | (0.222) |
| Father | 1.602^**^ | 1.173^***^ | -0.506 | -0.195 | 0.466 | 0.499 |
|  | (0.618) | (0.347) | (0.579) | (0.361) | (0.630) | (0.350) |
| Mother | -0.796 | -0.624 | 1.001 | 1.293^***^ | -0.455 | -0.063 |
|  | (0.543) | (0.360) | (0.556) | (0.349) | (0.649) | (0.328) |
| Couple | -1.338^*^ | -0.276 | 0.873 | 0.707^**^ | -0.988^*^ | -0.414 |
|  | (0.521) | (0.310) | (0.470) | (0.265) | (0.502) | (0.238) |
| BAME | 0.072 | 1.842^***^ | 2.214^*^ | 0.723 | -0.480 | -0.620 |
|  | (0.552) | (0.537) | (0.876) | (0.441) | (1.185) | (0.435) |
| Higher second.(Ref: GCSE) | 0.813 | 0.283 | -0.156 | 0.010 | -0.505 | 0.414 |
|  | (0.504) | (0.337) | (0.523) | (0.320) | (0.487) | (0.283) |
| University or above | 0.093 | -0.167 | -0.339 | 0.146 | -0.264 | 0.331 |
|  | (0.464) | (0.328) | (0.466) | (0.309) | (0.502) | (0.290) |
| Age centered | -0.018 | -0.028^**^ | -0.016 | -0.000 | 0.009 | 0.014 |
|  | (0.017) | (0.011) | (0.016) | (0.011) | (0.016) | (0.009) |
| Age centred squared | 0.044 | 0.262^**^ | -0.031 | -0.027 | 0.056 | -0.013 |
|  | (0.154) | (0.093) | (0.140) | (0.088) | (0.160) | (0.074) |
| Constant | 4.078^***^ | 3.058^***^ | -0.229 | -1.162^*^ | 0.016 | -0.915 |
|  | (0.753) | (0.574) | (0.743) | (0.506) | (0.804) | (0.478) |
| Childless women (ref: childless men) |  | 0.260^***^ |  | 0.277^***^ |  | 0.274^***^ |
|  |  | (0.022) |  | (0.022) |  | (0.022) |
| Father |  | -0.153^***^ |  | -0.178^***^ |  | -0.248^***^ |
|  |  | (0.035) |  | (0.036) |  | (0.037) |
| Mother |  | 0.073^*^ |  | 0.050 |  | 0.018 |
|  |  | (0.032) |  | (0.033) |  | (0.034) |
| Couple |  | 0.534^***^ |  | 0.456^***^ |  | 0.475^***^ |
|  |  | (0.027) |  | (0.027) |  | (0.028) |
| BAME |  | -0.666^***^ |  | -0.649^***^ |  | -0.691^***^ |
|  |  | (0.035) |  | (0.037) |  | (0.037) |
| Born in the UK |  | 0.428^***^ |  | 0.429^***^ |  | 0.427^***^ |
|  |  | (0.033) |  | (0.034) |  | (0.035) |
| Higher second.(Ref: GCSE) |  | 0.305^***^ |  | 0.297^***^ |  | 0.307^***^ |
|  |  | (0.024) |  | (0.025) |  | (0.026) |
| University or above |  | 0.655^***^ |  | 0.669^***^ |  | 0.664^***^ |
|  |  | (0.027) |  | (0.028) |  | (0.028) |
| Ln household net income |  | -0.291^***^ |  | -0.313^***^ |  | -0.322^***^ |
|  |  | (0.010) |  | (0.011) |  | (0.012) |
| Age centered |  | 0.003^**^ |  | 0.005^***^ |  | 0.005^***^ |
|  |  | (0.001) |  | (0.001) |  | (0.001) |
| Age centered squared |  | -0.112^***^ |  | -0.123^***^ |  | -0.126^***^ |
|  |  | (0.008) |  | (0.009) |  | (0.009) |
| Owned mortgage (ref: Owned outright) |  | -0.092^**^ |  | -0.090^**^ |  | -0.107^***^ |
|  |  | (0.030) |  | (0.031) |  | (0.032) |
| Rent-public |  | -0.596^***^ |  | -0.636^***^ |  | -0.643^***^ |
|  |  | (0.039) |  | (0.041) |  | (0.041) |
| Rent-private |  | -0.485^***^ |  | -0.501^***^ |  | -0.526^***^ |
|  |  | (0.041) |  | (0.043) |  | (0.044) |
| North (ref:London) |  | -0.165^***^ |  | -0.184^***^ |  | -0.179^***^ |
|  |  | (0.046) |  | (0.048) |  | (0.049) |
| Yorkshire & Humber |  | -0.142^**^ |  | -0.148^**^ |  | -0.156^**^ |
|  |  | (0.051) |  | (0.053) |  | (0.055) |
| Midlands |  | -0.069 |  | -0.069 |  | -0.068 |
|  |  | (0.044) |  | (0.046) |  | (0.047) |
| East |  | 0.070 |  | 0.103 |  | 0.102 |
|  |  | (0.052) |  | (0.054) |  | (0.055) |
| South |  | 0.022 |  | 0.018 |  | 0.025 |
|  |  | (0.043) |  | (0.045) |  | (0.046) |
| Wales/Scot/NI |  | -0.136^**^ |  | -0.161^***^ |  | -0.155^**^ |
|  |  | (0.044) |  | (0.046) |  | (0.047) |
| month# till Covid study |  | -0.050^***^ |  | -0.049^***^ |  | -0.048^***^ |
|  |  | (0.001) |  | (0.001) |  | (0.001) |
| Constant |  | 2.866^***^ |  | 3.014^***^ |  | 3.036^***^ |
|  |  | (0.106) |  | (0.113) |  | (0.117) |
| athrho |  | 0.005 |  | 0.043 |  | -0.008 |
|  |  | (0.031) |  | (0.030) |  | (0.031) |
| lnsigma |  | 2.300^***^ |  | 2.223^***^ |  | 2.099^***^ |
|  |  | (0.025) |  | (0.024) |  | (0.030) |
| R2 | 0.011 |  | 0.010 |  | 0.004 |  |
| Lambda |  | 0.053 |  | 0.396 |  | -0.065 |
| Rho |  | 0.005 |  | 0.043 |  | -0.008 |
| Chi-squared model test |  | 53.841 |  | 34.194 |  | 9.696 |
| P-value model test | 0.000 | 0.000 | 0.040 | 0.000 | 0.453 | 0.376 |
| Chi-squared comparison test |  | 0.029 |  | 1.981 |  | 0.064 |
| P-value comparison test |  | 0.864 |  | 0.159 |  | 0.800 |
| Number of iterations |  | 1 |  | 2 |  | 1 |
| Whether converged |  | 1 |  | 1 |  | 1 |
| Number of individuals | 8496 | 43152 | 7826 | 42482 | 7216 | 41872 |

Data: UKHLS Understanding Society wave 8-9 and Covid waves 1-3. Note: * p<0.05 ** p<0.01 *** p<0.001

Table A5. Change in subjective wellbeing

|  | W29 OLS | W29 Heckman | W30 OLS | W30 Heckman | W31 OLS | W31 Heckman |
| --- | --- | --- | --- | --- | --- | --- |
| Childless women (ref: childless men) | -0.759^**^ | -0.742^***^ | 0.671^**^ | 0.503^***^ | 0.359 | 0.281^*^ |
|  | (0.293) | (0.159) | (0.229) | (0.131) | (0.244) | (0.123) |
| Father | -0.617 | -0.692^**^ | -0.052 | 0.213 | 0.166 | -0.201 |
|  | (0.376) | (0.231) | (0.343) | (0.187) | (0.321) | (0.191) |
| Mother | -1.309^***^ | -1.384^***^ | 0.534 | 0.642^***^ | 0.508 | 0.034 |
|  | (0.347) | (0.221) | (0.399) | (0.186) | (0.293) | (0.182) |
| Couple | -0.309 | -0.149 | 0.308 | 0.159 | -0.178 | 0.213 |
|  | (0.298) | (0.183) | (0.244) | (0.141) | (0.236) | (0.143) |
| BAME | -0.339 | -0.321 | -0.610 | -0.122 | -0.087 | -0.187 |
|  | (0.697) | (0.248) | (0.414) | (0.200) | (0.407) | (0.199) |
| Higher second.(Ref: GCSE) | -0.013 | 0.015 | 0.217 | -0.011 | 0.234 | 0.095 |
|  | (0.337) | (0.186) | (0.285) | (0.149) | (0.266) | (0.144) |
| University or above | -0.388 | -0.274 | 0.032 | -0.209 | 0.284 | 0.210 |
|  | (0.311) | (0.197) | (0.252) | (0.151) | (0.263) | (0.146) |
| Age centered | 0.049^***^ | 0.033^***^ | -0.005 | -0.003 | 0.005 | -0.001 |
|  | (0.012) | (0.007) | (0.010) | (0.006) | (0.010) | (0.006) |
| Age centred squared | -0.277^**^ | -0.253^***^ | -0.035 | 0.088 | 0.107 | 0.029 |
|  | (0.094) | (0.058) | (0.105) | (0.049) | (0.087) | (0.049) |
| Constant | 0.239 | -0.016 | -0.722^*^ | -0.381 | -0.497 | -0.581^*^ |
|  | (0.433) | (0.328) | (0.300) | (0.268) | (0.386) | (0.262) |
| Childless women (ref: childless men) |  | 0.252^***^ |  | 0.275^***^ |  | 0.271^***^ |
|  |  | (0.021) |  | (0.022) |  | (0.022) |
| Father |  | -0.192^***^ |  | -0.177^***^ |  | -0.255^***^ |
|  |  | (0.035) |  | (0.036) |  | (0.037) |
| Mother |  | 0.044 |  | 0.042 |  | 0.003 |
|  |  | (0.032) |  | (0.033) |  | (0.034) |
| Couple |  | 0.531^***^ |  | 0.452^***^ |  | 0.479^***^ |
|  |  | (0.027) |  | (0.027) |  | (0.028) |
| BAME |  | -0.658^***^ |  | -0.648^***^ |  | -0.675^***^ |
|  |  | (0.035) |  | (0.037) |  | (0.038) |
| Born in the UK |  | 0.421^***^ |  | 0.428^***^ |  | 0.430^***^ |
|  |  | (0.033) |  | (0.034) |  | (0.035) |
| Higher second.(Ref: GCSE) |  | 0.301^***^ |  | 0.289^***^ |  | 0.306^***^ |
|  |  | (0.024) |  | (0.025) |  | (0.026) |
| University or above |  | 0.645^***^ |  | 0.659^***^ |  | 0.662^***^ |
|  |  | (0.026) |  | (0.027) |  | (0.028) |
| Ln household net income |  | -0.288^***^ |  | -0.311^***^ |  | -0.321^***^ |
|  |  | (0.010) |  | (0.011) |  | (0.012) |
| Age centered |  | 0.002^**^ |  | 0.005^***^ |  | 0.005^***^ |
|  |  | (0.001) |  | (0.001) |  | (0.001) |
| Age centred squared |  | -0.113^***^ |  | -0.124^***^ |  | -0.126^***^ |
|  |  | (0.008) |  | (0.009) |  | (0.009) |
| Owned mortgage (ref: Owned outright) |  | -0.098^**^ |  | -0.093^**^ |  | -0.106^***^ |
|  |  | (0.030) |  | (0.031) |  | (0.031) |
| Rent-public |  | -0.598^***^ |  | -0.628^***^ |  | -0.641^***^ |
|  |  | (0.039) |  | (0.040) |  | (0.041) |
| Rent-private |  | -0.502^***^ |  | -0.508^***^ |  | -0.536^***^ |
|  |  | (0.041) |  | (0.043) |  | (0.044) |
| North (ref: London) |  | -0.161^***^ |  | -0.177^***^ |  | -0.177^***^ |
|  |  | (0.046) |  | (0.048) |  | (0.049) |
| Yorkshire & Humber |  | -0.145^**^ |  | -0.152^**^ |  | -0.153^**^ |
|  |  | (0.051) |  | (0.053) |  | (0.055) |
| Midlands |  | -0.059 |  | -0.062 |  | -0.058 |
|  |  | (0.044) |  | (0.046) |  | (0.047) |
| East |  | 0.093 |  | 0.103 |  | 0.103 |
|  |  | (0.052) |  | (0.054) |  | (0.056) |
| South |  | 0.027 |  | 0.017 |  | 0.030 |
|  |  | (0.043) |  | (0.045) |  | (0.046) |
| Wales/Scot/NI |  | -0.124^**^ |  | -0.159^***^ |  | -0.151^**^ |
|  |  | (0.044) |  | (0.046) |  | (0.047) |
| month# till Covid study |  | -0.050^***^ |  | -0.049^***^ |  | -0.049^***^ |
|  |  | (0.001) |  | (0.001) |  | (0.001) |
| Constant |  | 2.888^***^ |  | 3.026^***^ |  | 3.037^***^ |
|  |  | (0.105) |  | (0.112) |  | (0.117) |
| athrho |  | 0.001 |  | -0.036 |  | 0.029 |
|  |  | (0.031) |  | (0.032) |  | (0.032) |
| lnsigma |  | 1.836^***^ |  | 1.580^***^ |  | 1.509^***^ |
|  |  | (0.011) |  | (0.013) |  | (0.015) |
| R2 | 0.020 |  | 0.006 |  | 0.003 |  |
| Lambda |  | 0.009 |  | -0.174 |  | 0.133 |
| Rho |  | 0.001 |  | -0.036 |  | 0.029 |
| Chi-squared model test |  | 107.649 |  | 28.070 |  | 12.782 |
| P-value model test | 0.000 | 0.000 | 0.010 | 0.001 | 0.447 | 0.173 |
| Chi-squared comparison test |  | 0.002 |  | 1.250 |  | 0.830 |
| P-value comparison test |  | 0.963 |  | 0.264 |  | 0.362 |
| Number of iterations |  | 1 |  | 1 |  | 1 |
| Whether converged |  | 1 |  | 1 |  | 1 |
| Number of individuals | 8634 | 43290 | 7930 | 42586 | 7296 | 41952 |

Data: UKHLS Understanding Society wave 8-9 and Covid waves 1-3. Note: * p<0.05 ** p<0.01 *** p<0.001

Table A6. Change in weekly childcare time among parents

|  | W30 OLS | W30 Heckman | W31 OLS | W31 Heckman |
| --- | --- | --- | --- | --- |
| Women | -0.285 | -0.360 | 0.643 | 0.140 |
|  | (1.586) | (1.095) | (1.652) | (1.085) |
| Child age 5-15 | 2.259 | 2.756 | 0.328 | -0.778 |
|  | (2.639) | (1.728) | (2.195) | (1.489) |
| Couple | -3.986 | -1.626 | -0.163 | -2.957 |
|  | (3.813) | (1.959) | (3.599) | (2.413) |
| BAME | -1.535 | 0.735 | 1.975 | 0.655 |
|  | (2.158) | (1.478) | (1.781) | (1.421) |
| Higher second.  (Ref: GCSE) | 1.236 | -0.153 | 0.718 | -1.720 |
|  | (2.757) | (1.747) | (2.703) | (1.644) |
| University or above | -1.242 | -0.730 | 3.144 | -0.094 |
|  | (2.136) | (1.643) | (2.597) | (1.607) |
| Age centered | 0.095 | 0.068 | 0.039 | 0.141 |
|  | (0.091) | (0.093) | (0.125) | (0.097) |
| Age centered squared | 0.961 | -0.955 | 0.456 | 0.345 |
|  | (1.079) | (0.672) | (1.033) | (0.682) |
| Constant | 1.964 | 0.123 | -6.916 | -0.637 |
|  | (4.978) | (4.279) | (5.484) | (3.868) |
| Women |  | 0.075^*^ |  | 0.100^**^ |
|  |  | (0.031) |  | (0.031) |
| Child age5-15 |  | 0.672^***^ |  | 0.653^***^ |
|  |  | (0.054) |  | (0.055) |
| Couple |  | 0.429^***^ |  | 0.485^***^ |
|  |  | (0.066) |  | (0.068) |
| BAME |  | -0.575^***^ |  | -0.540^***^ |
|  |  | (0.061) |  | (0.062) |
| Born in the UK |  | 0.541^***^ |  | 0.522^***^ |
|  |  | (0.059) |  | (0.060) |
| Higher second.  (Ref: GCSE) |  | 0.238^***^ |  | 0.277^***^ |
|  |  | (0.048) |  | (0.048) |
| University or above |  | 0.728^***^ |  | 0.735^***^ |
|  |  | (0.050) |  | (0.050) |
| Ln household net income |  | -0.389^***^ |  | -0.418^***^ |
|  |  | (0.032) |  | (0.037) |
| Age centered |  | -0.047^***^ |  | -0.048^***^ |
|  |  | (0.003) |  | (0.004) |
| Age centered squared |  | 0.064^*^ |  | 0.049 |
|  |  | (0.026) |  | (0.026) |
| Owned mortgage  (ref: Owned outright) |  | -0.045 |  | -0.057 |
|  |  | (0.072) |  | (0.073) |
| Rent-public |  | -0.824^***^ |  | -0.780^***^ |
|  |  | (0.091) |  | (0.091) |
| Rent-private |  | -0.670^***^ |  | -0.666^***^ |
|  |  | (0.093) |  | (0.094) |
| North (ref: London) |  | -0.229^**^ |  | -0.329^***^ |
|  |  | (0.086) |  | (0.085) |
| Yorkshire & Humber |  | -0.254^**^ |  | -0.356^***^ |
|  |  | (0.092) |  | (0.093) |
| Midlands |  | -0.154 |  | -0.190^*^ |
|  |  | (0.082) |  | (0.082) |
| East |  | 0.104 |  | 0.037 |
|  |  | (0.094) |  | (0.096) |
| South |  | -0.072 |  | -0.128 |
|  |  | (0.081) |  | (0.081) |
| Wales/Scot/NI |  | -0.242^**^ |  | -0.258^**^ |
|  |  | (0.083) |  | (0.084) |
| month# till Covid study |  | -0.046^***^ |  | -0.046^***^ |
|  |  | (0.001) |  | (0.002) |
| Constant |  | 2.726^***^ |  | 2.932^***^ |
|  |  | (0.293) |  | (0.326) |
| athrho |  | 0.014 |  | 0.027 |
|  |  | (0.078) |  | (0.065) |
| lnsigma |  | 3.288^***^ |  | 3.223^***^ |
|  |  | (0.036) |  | (0.040) |
| R2 | 0.008 |  | 0.003 |  |
| Lambda |  | 0.368 |  | 0.681 |
| Rho |  | 0.014 |  | 0.027 |
| Chi-squared model test |  | 10.380 |  | 6.177 |
| P-value model test | 0.153 | 0.239 | 0.878 | 0.627 |
| Chi-squared comparison test |  | 0.031 |  | 0.173 |
| P-value comparison test |  | 0.860 |  | 0.678 |
| Number of iterations |  | 2 |  | 2 |
| Whether converged |  | 1 |  | 1 |
| Number of individuals | 2374 | 14403 | 2241 | 14270 |

Data: UKHLS Understanding Society wave 8-9 and Covid waves 1-3.

Note: * p<0.05 ** p<0.01 *** p<0.001
