# Supplementary material for: The varying impacts of COVID-19 and its related measures in the UK: A year in review
Source: PLoS One. 2021 Sep 29;16(9):e0257286. doi: 10.1371/journal.pone.0257286 (PMC8480884; doi:10.1371/journal.pone.0257286)
Supplement: Attachment — Submitted filename: PlosOne_r1_Response to Reviewers.docx [file pone.0257286.s009.docx]

**Response to Reviewers**

**Reviewer #1:**

The purpose of this paper is to examine differential impact on societal groups of lockdown during the first wave of COVID-19 in UK. The ‘impact’ (the dependent variable of the analysis) is represented by four alternative measures of labour market participation indicators (monthly income, weekly working hours, weekly housework hours, weekly child care hours and subjective wellbeing (outcome variables). Twelve societal groups considered (independent variables) are delineated based on gender, ethnicity, educational level, and parental status. The analysis is based on a panel dataset constructed by putting together data from an earlier (pre-COVID-19) wave of the UK Household Longitudinal Survey (UKHLS) and online questionnaire survey of individuals who participated in UKHLS) conducted in March, April, and, June using the same sample frame.

The paper has examined how the impact of the lockdown varied among the identified societal groups compared to the pre-lockdown situation. This is obviously an important research issue for assessing the developmental impact of the pandemic. However, I do not find that the authors' claim that the paper provides ‘new insights into the long-term impact of the pandemic' is warranted. This is essentially a short-term analysis covering a period of just four months. The term ‘Longitudinal’ analysis is really a mismove for an analysis covering such a short period of time.

Thanks for the comments. We have used latest data until late March 2021 to examine the dynamics of earnings, time use, and subjective wellbeing as the pandemic developed in the UK over the past year.

It is important to clearly spell out the dependent and independent (explanatory) variables of the analysis and the hypothesized relationship between them as part of the introductory section.

Thanks for the comments. Please see the revised introductory section. In the second last paragraph (pp. 2), we mentioned that: “*In this paper, we contribute to the COVID-19 research by providing a dynamic picture of how people’s labor earnings, time use, and wellbeing have changed across different stages of the pandemic. We investigate whether, and the extent to which, the inequalities on these outcomes by gender, ethnicity, and educational level, have changed over the past year*.”

The short literature survey in the second and third paragraphs of the introduction section is not well integrated in the discussion. It is important to draw on this literature clearly spell out the hypothesised relationships between the dependent and explanatory variables in order to set the stage for the ensuing empirical analysis. Indeed, the absence of a clear statement of the underlying hypothesis is a major limitation of the paper.

Thanks for the suggestions. We have provided a more detailed and up-to-date literature review in the section titled “*The impacts of the COVID-19 and its related measures*” (pp. 2-4). We have simplified the analysis by focusing on (1) the trajectories of earnings, working time, subjective wellbeing, housework time, and childcare time and (2) differences in those trajectories across gender, race/ethnicity, and education groups. The conventional hypothesis derivation writing style does not fit well to this paper that is mainly descriptive. In addition, as the revised literature review part, previous findings are not always consistent (mentioned on pp.4).

We highlight the purpose of this research again in the last paragraph of this section (pp.4): “*In this article, we will provide a first-year review of the COVID-19 development in the UK and document how people have responded to the first lockdown, the ease of the first lockdown, and the latter two lockdowns. This evaluation could answer whether people responded similarly to repeated lockdowns and whether these changes in earnings, time use, and feelings are temporary or long-lasting.*”

The results reported in Figures 1 to 5 (to me these look like ‘Tables’, not ‘Figures’!).
It is not clear to me why the dependent variables are included in ‘difference’ forms, eg childless women vs childless men, fathers versus childless men, mothers versus childless men etc. Why not include each variable of societal characteristic in its own as separate variable: childless women, childless men, fathers, childless men, mothers, childless men etc. The pairwise inclusion of variables essential mean implicitly imposing the same regression coefficient on both variable in a given pair for a for a given explanatory variable, which is methodologically questionable. Including the variables separately helps appropriately interpreting the coefficients among the different societal groups based on statistical differences among them.

We have completely revised the results section with the latest data and a simple fixed-effect regression analysis. Please refer to the section titled “Analytical strategies” (pp.8) and the results section (pp.8-18). We hope that now the presentation of the results is easier to understand and those “coefficient plots” now look more like “figures”.

The results reported in the give Figures (Tables) 1 to 5 are very difficult to read because of the mixing of OLS and Hickman results in a given column using a very small font. You should report the two alternative estimates in separate columns. Also, it is important to report the t-ratio or standard error (or p-value) under each regression coefficient. Without this information it is not possible to meaningfully interpret the results.

We have completely revised the results section (pp.8-18) with the latest data and a simple fixed-effect regression analysis. We have revised those figures presenting the point estimate together with confidence intervals which would directly visualize the statistical significance level. We add in p values when discussing specific group differences.

For example, “*First, the drop in earnings for female workers (those who worked in Jan/Feb 2020) was smaller than male workers during the first lockdown in April 2020 (p=0.010).”* [pp.12]


Comparison of OLS and Hickman results suggest that there are notable differences in coefficient estimates. Given that Hickman results are methodologically superior to OLS results (for the reason discussed in the text) why not report only the Mickman results in the text and put OLS results for comparison in an appendix.

We have completely revised the results section with the latest data and a simple fixed-effect regression analysis. We dropped Heckman results because they did not add much to the findings.

It is important to explain how change in subjective wellbeing is defined/measured.

Please refer to the revised discussion on the measurement of subjective wellbeing in the section titled “Measures” [pp.7]. The use of the 12 items in the General Health Questionnaire (GHQ-12) is a common practice to measure subjective wellbeing in surveys.

The conclusion needs to be rewritten addressing the point I have made in the second paragraph above.

Please refer to the completely revised discussion section [pp18-20].

**Reviewer #2:**

This study provides a timely evaluation of changes in income, employment, household responsibilities, and general well-being throughout the course of the pandemic-related lockdown in the UK. The study advantageously uses panel data and measures monthly changes in the outcomes at each stage of the lockdown.

I believe the study provides important and interesting contributions, but the study could do more to maximize the strengths of its panel data. Otherwise, it’s not clear how it’s findings substantively improve over prior research using cross-sectional data that the study cites.

1. Clarify and Strengthen the Panel Data Analysis as the Central Contribution

a. A clearer explanation in the front end of the focus on within-person changes in the outcomes would communicate the article’s contribution more strongly. Currently, the manuscript’s front end emphasizes aggregate changes over time throughout the lockdown and inequalities across groups (pages 2-3). However, repeated cross-sectional data would be able to assess these topics. For example, repeated cross-sectional data could document “how initial inequalities by gender, ethnicity, educational level, and parental status, might have been altered at different stages of the lockdown.” Repeated cross-sections would be suitable for analyzing trends in these aggregate inequalities.

Many thanks for the comments. We have now completely revised the manuscript and the analysis. The contribution now is (1) to provide a one year review of the dynamics of earnings, time use, and subjective wellbeing at different stages of the pandemic, and (2) to analyze whether these trajectories differ across different genders, race/ethnicities, and educational groups. We highlight the purpose of this research again in the second last paragraph of this section [pp.2]: “*In this paper, we contribute to the COVID-19 research by providing a dynamic picture of how people’s labor earnings, time use, and wellbeing have changed across different stages of the pandemic. We further investigate whether, and the extent to which, the inequalities on these outcomes by gender, ethnicity, and educational level, have changed over the past year.*”

We adopted a fixed-effect regression approach to present within-individual changes in five outcomes—earnings, paid work time, subjective wellbeing, housework time, and childcare time at different stages of this pandemic. We have included partnership status and parenthood status to consider the impacts due to changes in family circumstances [see pp.7-8].

Repeated cross-sectional data should be able to access some of the outcomes, such as labor earnings. But for other outcomes such as time use and subjective wellbeing, so far cross-sectional data have not established good measures, especially measures before the pandemic. Also, given that we have followed up the pandemic for one year, those changes in personal circumstances are difficult to be captured and matched to the same individual using cross-sectional data.

b. The panel data analyzed in this article are uniquely able to examine within-person changes between these groups—a clear advantage over cross-sectional data. But as it stands, the mean changes (within persons) the paper currently presents are not so different from changes in mean differences between groups that one could obtain from repeated cross-sectional data.

Many thanks for the comments. Please see the response above.

The study could strengthen the panel analysis with more detailed within-person measures. For example, measures of earnings and hours volatility within persons across several periods (like the coefficient of variation or something) would give a more complete picture of between-group differences in overall instability throughout the lockdown.

Many thanks for the helpful comments. We have now completely revised the manuscript and the analysis to incorporate these comments. Now we have provided a month-by-month dynamics picture of those outcome variables in general and across three different social groups—gender, race/ethnicity, and education **from before the pandemic up to the point of late March 2021**.


2. Heckman Selection Models

Can the authors explain the assumptions and goals of the Heckman selection models in more detail (pages 6 & 7)?

Many thanks for the comments. We have now completely revised the manuscript and the analysis. We have adopted a simpler fixed-effect regression analysis. We use the weights provided in the dataset that have considered of sample selection and nonresponse to address this issue [pp.8]. Previous work using the same data has all adopted this weighting approach. We also find that the use of the Heckman model does not add much to our findings and thus dropped it.

My understanding is that the selection model essentially requires variables similar to instrumental variables (affecting selection but not the outcomes) for the selection process. Otherwise, the selection model could absorb too much variation from the explanatory variables in the outcome model, undermining the outcome model estimates’ precision. It’s not clear to me whether any of the listed variables would satisfy those assumptions. Moreover, Heckman selection models can only account for observable selection processes.

Many thanks for the comments. We have now completely revised the manuscript and the analysis. We have adopted a simpler fixed-effect regression analysis. Please see the response above.

**Reviewer #3:**

This research addresses the impact in the UK of COVID-19 at various stages of the lockdown using the UK Household Longitudinal Survey (UKHLS). More specifically, these impacts are labour income, time used, and subjective well-being and are measured before, during, and after the lockdown period (i.e., before March, April, and June 2020). The authors find that that the impacts along these three metrics differ depending on individuals' gender, parental status, ethnicity and education level.

This is an important and timely piece of work which adds to the (increasingly) voluminous literature on the social impacts of COVID-19. Given its timeliness, and the limited time to think about some of the issues raised by the paper, my remarks will be brief.

My biggest concern is that the actual effects of the pandemic are not being captured in this research given the relatively short-time period under consideration. Furthermore, while causality is never assumed throughout the paper, the authors are correct in pointing out (page 3, second paragraph) that there have been many factors that have changed over this brief period “on a weekly or even daily basis . . .” That said, there is little that can be done about this given the data, short of re-estimating the models with more up-to-date data (if available). Still, I would like to see this point made clearer and that these results are not dealing with the long-term (as I understand it), despite the implication at the beginning of this paragraph that this is what the paper does. Further, if the data can be updated in a timely manner, this would be useful and give us better insights into the medium-term impacts of the lockdown.

Many thanks for the helpful comments. We have now completely revised the manuscript and the analysis with latest data now covering the pre-pandemic period till late March 2021. The contribution now is to provide a first-year review of the dynamics of earnings, time use, and subjective wellbeing as the pandemic develop from March 2020 to March 2021. We hope the substantially extended examining period would be sufficient to justify our contribution.

We highlight the purpose of this research again in the second last paragraph of this section [pp.2]: “*In this paper, we contribute to the COVID-19 research by providing a dynamic picture of how people’s labor earnings, time use, and wellbeing have changed across different stages of the pandemic. We further investigate whether, and the extent to which, the inequalities on these outcomes by gender, ethnicity, and educational level, have changed over the past year.*”

On a related note, much of the discussion in the text has to do with the differences between the pre- and post-lockdown periods where the description is putting together the results from pre-lockdown versus April, April vs. May, and May vs. June (i.e., the three columns in Figures 1-3 and 5). I think it would be worthwhile to include a new set of results that would directly compare the pre- and post-lockdown data. These should be very similar to the results described in the text but would be much easier for the reader to interpret visually. This could easily be accomplished by adding a new column to the figures.

We have completely revised the analysis and provided new figures to easily show these changes in a month-by-month manner.

Minor points:

Page 2, first intro paragraph – Relative time measures should be avoided. Here it states “six months after the UK announced the lockdown on 23 March 2020 . . . “As I read this paper, it is now eight months after this date. Further, this two-month lag is an eternity given the number of papers that have been written in response to the pandemic. I would suggest that a quick update of the literature where necessary and possible.

Many thanks for the comments. We have used latest data up to the point of late March 2021 and provided a new literature review section titled “*The impacts of the COVID-19 and its related measures*” [pp.2-4].

Page 3, bottom – I assume that the measures taken in the UK were national in scope? This should be made clear since in many other countries any lockdown measures have been at the discretion of subnational levels of government.

The March 2020, November 2020, and January 2021 lockdowns are at the UK national level. Please refer to the revised introduction of the UK lockdown timeline in the section titled “*Timeline of the lockdown measures in the UK*” [pp.4-5].

Page 13, second paragraph – the difference in the increase in housework time of 0.6 to 0.8 hours for mothers and childless men is not statistically significant (according to Figure 3). Such results here (and elsewhere) should probably not be reported (or at the very least identified as statistically insignificant).

We have completely revised the analysis and provided new figures to report these estimates. We have dropped the parent vs childless analysis to simplify this work.

Page 15, paragraph 5 – do we know that childless women and mothers experienced less reductio in their working hours due to their relatively high proportions working in key sectors, or is this speculation?

Thanks for the comment. We have completely revised the analysis and provided new figures. We have dropped the parent vs childless analysis to simplify this work.

Throughout – it is quite possible that there may be some seasonal effects at play in these estimates. The proper treatment of this might be to do a difference-in-difference estimation which would compare the pre- and post-lockdown period in 2020 with the same data from earlier years. I do not expect this to be done but mention of these potential effects might be worthwhile. The authors allude to these in the conclusion when they discuss needing further waves of the data.

Thanks for the comment. We have mentioned this limitation in the *third last paragraph* in the discussion section and we highlight them below [pp.19]:

“*One limitation of this study is that some changes could be brought by seasonal fluctuations beyond the COVID-19 and its related restrictions. For example, people’s paid work time in winter may differ from that in summer. The general psychological health was usually worse in winter than in summer [49]. The ideal solution is to compare information collected in the same month before the pandemic and in 2020. However, this approach is not possible with the current data. If the current survey retains the current monthly or bi-monthly data collection frequency, future work can compare the same month in 2020 and the years after to examine pandemic and post-pandemic differences. In summary, our results show the trajectories of earnings, time use, and subjective wellbeing at different time point over the past year but cannot identify the exact impact of a specific lockdown restrictive policy.*”

**Reviewer #4:**

Report on: The Varying Impacts of COVID-19 on Labor Earnings, Time Use and Well-Being at Different Stages of the United Kingdom Lockdown (MS# PONE-D-20-32060)

As the title suggests, the paper quantifies changes in well-being for a national sample during April-June 2020 when COVID-19 and related policy measures had major impacts on United Kingdom residents. Across all measures, the paper finds worse outcomes in April, a small rebound in May and little or no impact in June. The Abstract concludes “different vulnerabilities of different social groups to the lockdown measures not only affect the extent of the Covid-19 impact, but also the speed at which these effects develop.”

The paper uses good methods to document how income and behavior changed over the focal three months but is less convincing in attributing ALL the changes to the Covid-19 pandemic and subsequent lockdown measures. In other words, the paper documents correlation but not causation.

Many thanks for the comments. The purpose of the revised manuscript is to provide a first-year review of the dynamics in earnings, time use, and subjective wellbeing in the UK as the pandemic developed. We have revised the manuscript to be careful in the wording to say that these relationships are associations and are specific to that time point. For example, we have flagged this in the first sentence of the Abstract: “*We examine how earnings, time use, and subjective wellbeing of different social groups had changed at different stages/waves of the pandemic in the United Kingdom (UK).”*

We have also put a caveat in the discussion section, highlighting that “*In summary, our results show the trajectories of earnings, time use, and subjective wellbeing at different time point over the past year but cannot identify the exact impact of a specific lockdown restrictive policy*.”” –the third last paragraph of the discussion section [pp.19].

A timeline of major regulatory events and their implementation lags would have been very helpful. It seems implausible that all policy measures would have instantaneous effects as currently assumed, or that individuals would not have changed their behavior voluntarily even absent government mandates.

Thanks for the comment. Please refer to the revised section titled “*Timeline of the lockdown measures in the UK*” [pp.4-5]. We also provide **a link** with a timeline map showing all the major changes of the COVID-induced measures. We have used the latest dataset lasting till late March 2021. Overall, this is a descriptive work evaluating trajectories of earnings, time use, and subjective wellbeing of the whole sample and by subgroups over the past year.

We highlight this on page 4: “*In this article, we will provide a first-year review of the COVID-19 development in the UK and document how people have responded to the first lockdown, the ease of the first lockdown, and the latter two lockdowns. This evaluation could answer whether people responded similarly to repeated lockdowns and whether these changes in earnings, time use, and feelings are temporary or long-lasting.*”

The marked-up paper identifies minor editorial suggestions. More detailed comments follow:
1. Given the stated desire to study nationwide long-term impacts of COVID-19, it is strange that the paper relies only on stale (June 2020) survey data from the United Kingdom Household Longitudinal Survey (UKHLS) rather than supplementing with more recent unemployment data from the Office of National Statistics, available monthly through November 2020. (<https://www.ons.gov.uk/employmentandlabourmarket/peopleinwork/employmentandemployeetypes/bulletins/employmentintheuk/november2020>)

Many thanks for providing the editorial suggestions. We have revised the manuscript accordingly.

We have now used a more updated data lasting until March 2021. The advantage of this data is the comprehensive collection of multiple variables that we are interested, such as housework time, subjective wellbeing, and childcare time and associated individual characteristics – such as their partnership status and parenthood status, which are likely to have changed over the past year.

Repeated cross-sectional data should be able to access some of the outcomes, such as labor earnings. But for other outcomes such as time use and subjective wellbeing, so far cross-sectional data have not established good measures, especially measures before the pandemic. Also, given that we have followed up the pandemic for one year, those changes in personal circumstances are difficult to be captured and matched to the same individual using cross-sectional data.

2. Similar tests have been published for the April and May 2020 waves of the UKHLS COVID-19 surveys. The paper adds tests for the June 2020 wave and finds little changes during this month.

We have now used a more updated data up to March 2021 to provide new dynamics in earnings, time use, and subjective wellbeing over the past year and across different social groups.

We have added a more updated and detailed literature review section in the revised manuscript—section titled “*The impacts of the COVID-19 and its related measures*” [pp.2-4].

3. Many variables in the Supplementary are undefined making it difficult to interpret the results. The Tables would ideally be self-contained.

We have completely revised our analysis. Now the estimates reported in the new **Supporting information** at the end of the manuscript are easier to read than in the previous version.

4. The paper should better justify why standard errors are clustered at the household level rather than by gender or region, since the effects are theorized to covary within groups.

We have now used a simpler fixed-effect regression approach to report within-individual changes [pp.8].

Based on the recently highly influential work of Alberto Abadie et al. (2017), clustering standard errors should be used for (1) a sampling design reason, which arises because you have sampled data from a population using clustered sampling, and want to say something about the broader population; and (2) an experimental design reason, where the assignment mechanism for some causal treatment of interest is clustered.

For the sampling method of this data, gender, race/ethnicity, and education are not used to sample. We do not randomly sample some gender, some race/ethnicity, or some educational groups. Therefore, generally, we do not cluster by gender, race/ethnicity, and educational level.

The second reason for clustering is that when cluster of units, rather than individuals, are assigned to a treatment. Our case does not apply to this reason because individuals of all genders, race/ethnicity, and education levels have experienced the pandemic in the UK.

In the current data, the samples are probability samples of postal address. They are clustered and stratified. This sampling method fits the cluster reason (1). Therefore, we have used clustered standard error considering this sampling design and do not apply clustered standard error at the gender, race/ethnicity, or education level. We have mentioned this in the second last paragraph of the section titled “Analytical strategies” [pp.8]—“ *Clustered standard errors are used to consider the sampling design of the UKHLS survey [42].”*

Reference

*[42] Alberto Abadie & Susan Athey & Guido W. Imbens & Jeffrey Wooldridge, 2017. "When Should You Adjust Standard Errors for Clustering?," NBER Working Papers 24003, National Bureau of Economic Research, Inc.*

5. The Heckman procedure for self-selection in responses seems appropriate for the research setting. However, most of the p-values from the comparison tests (8 of 14) reported in the Supplementary are well above 0.10 significance level, suggesting that the Heckman selection models have low predictive power. The paper does not discuss this low power in discussing results or drawing inferences.

We have now used a simpler fixed-effect regression approach to report within-individual changes [pp.8]. We now apply weights provided in the raw data to consider sample selection and nonresponse. This approach has been adopted by many studies using the same dataset. second last paragraph of the section titled “Analytical strategies [pp.8]—“Weights are used to take into account unequal selection probabilities and differential non-response to approximate a population-level analysis. This approach has been used in previous work analyzing the same data, for example, [2,3,16,19].”

We also find out that Heckman selection model does not add much to our findings and thus dropped this model.

6. Likewise, almost half of the OLS models (6 of 14) have model p-values that exceed the 0.10 significance level, indicating low reliability. Importantly, 4 of 5 models run on Wave 31 (July 2020) data are not significant at the 0.10 significance level. The reported R2s are often close to zero, suggesting that the adjusted R2s are negative. In other words, the reported results are like using a random number generator to measure most of the independent variables in about half the tests.

We have now used a simpler fixed-effect regression approach to report within-individual changes. Please refer to the updated results section.

7. Interpretation of the novel June 2020 results are clouded by the problem that “absence of evidence is not evidence of absence.” Thus, while it is descriptively valid to say that there were no net changes in June 2020, this is not the same as saying that COVID-19 and the lockdown measures had no impact, because some measures such as the Coronavirus Job Retention Scheme may have fully offset the impact of COVID-19, but each of these components may have had large effects considered individually.

Many thanks for the comments. We have now revised the whole results section and are more careful in interpreting the results. We added a caveat on page 19 of the Discussion and Conclusion section: “*In summary, our results show the trajectories of earnings, time use, and subjective wellbeing at different time point over the past year but cannot identify the exact impact of a specific lockdown restrictive policy.*”

8. The paper should be more cautious in interpreting the results. Given that there may have been important policy changes beyond those related to COVID-19, as well as voluntary changes in behavior, all the paper reports is the net effect of all changes. The lack of change in June 2020 is still interesting, but at best the paper is still studying short-term or medium-term effects rather than long-term effects. Low explanatory power of the models should be discussed explicitly.

We have now used a simpler fixed-effect regression approach to report within-individual changes. Data used now have lasted until late March 2021.

We have also put a caveat in the discussion section [pp.19], highlighting that “*In summary, our results show the trajectories of earnings, time use, and subjective wellbeing at different time point over the past year but cannot identify the exact impact of a specific lockdown restrictive policy*.”” –the third last paragraph of the discussion section.

For fixed-effect regressions, the low within-individual R-square is not uncommon, especially when predicting housework time and subjective wellbeing. For example, Borra, Browning, and Sevilla Sanz 2017, Foa, R.S., Gilbert, S. and M. Fabian. (2020), and Mari, G. (2019). In our case, this is most likely due to limited within-panel variation of time-varying predictors. So, the feature of our sample should be that only very few individuals have changed their partnership and parenthood status though their outcome variables—earnings, time use, and subjective wellbeing have changed a lot over the past year. The inclusion of more time-varying variables might be able to improve the explanatory power, such as whether furloughed, whether participated in the Job Retention Scheme, or whether infected by COVID-19. However, the purpose of this paper is to provide an overall net impact of the COVID-19 and its related measures instead of focusing on a specific policy or COVID-19 per se. Given the focus on the trajectories of earnings, time use, and subjective wellbeing as the pandemic develops, we do not include more time-varying variables.

We have added the above discussions in the paragraph **above the section** titled “Discussion and Conclusion” [pp.17-18].

Reference

*Borra, Cristina and Browning, Martin and Sevilla Sanz, Almudena, Marriage and Housework. IZA Discussion Paper No. 10740, Available at SSRN:* [*https://ssrn.com/abstract=2960549*](https://ssrn.com/abstract=2960549)

*Foa, R.S., Gilbert, S. and M. Fabian. (2020). “COVID-19 and Subjective Well-Being: Separating the Effects of Lockdowns from the Pandemic”. Cambridge, United Kingdom: Bennett Institute for Public Policy.*

*Mari, G. (2019), Is There a Fatherhood Wage Premium? A Reassessment in Societies With Strong Male-Breadwinner Legacies. J. Marriage Fam, 81: 1033-1052. https://doi.org/10.1111/jomf.12600*
