# Supplementary material for: The varying impacts of COVID-19 and its related measures in the UK: A year in review
Source: PLoS One. 2021 Sep 29;16(9):e0257286. doi: 10.1371/journal.pone.0257286 (PMC8480884; doi:10.1371/journal.pone.0257286)
Supplement: Attachment — Submitted filename: r2_Response to Reviewers.docx [file pone.0257286.s013.docx]

Response to Reviewers

As the title suggests, the paper quantifies changes in well-being for a national sample during April 2020-March 2021 when COVID-19 and related policy measures had major impacts on United Kingdom residents. Across all measures, the paper finds worse outcomes during lockdown periods (April 2020, November 2020 and January 2021) compared to non-lockdown periods, which are concentrated among women (except earnings), Black, Asian and Minority ethnic (BAME) immigrants (except depression), and non-degree holders. The Abstract concludes “the extent of the impacts of the COVID-19 and the COVID-induced measures as well as the speed at which these effects develop, vary across social groups with different types of vulnerabilities.”

The paper uses good methods to document how income and behavior changed over the last twelve months but is less convincing in attributing *ALL* the changes to the Covid-19 pandemic and subsequent lockdown measures. In other words, the paper documents correlation but not causation. It seems implausible that all policy measures would have instantaneous effects as currently assumed, or that individuals would not have changed their behavior voluntarily even absent government mandates. Put more starkly, the key driving variable is likely COVID infection rates (and death rates) rather than lockdown policies, which are a lagged response reflecting common causation. The paper should plot the national COVID-19 infection and death rates for the same months as the surveys (and include as an explanatory variable in regressions) to test if these rates explain outcomes better than the lockdown policies. The marked-up paper identifies minor editorial suggestions.

Thanks for the comment. As mentioned by the reviewer, we analyzed the changes in income, time use, and subjective wellbeing during a very special period of this COVID-19 pandemic. This pandemic is the most important incident that happened over the past year. We believe that the documented changes lend strong support that people’s income, time use, and subjective wellbeing are primarily affected by this pandemic together with its related policies in the UK.

We do not try to argue that we provide an exact estimation of these impact. We also have no intention to separate the impact of the spread of COVID-19 in the UK and policies that aim to contain this virus.

We aim to document the trends of these outcomes at different time points to shed light on the longer-term changes in those outcomes over the past year. This is an advancement from previous studies. It is important to note that these changes did correspond to the specific lockdown time points. It should be reasonable to attribute these changes to the spread of COVID-19 and its related measures. In the last year, the political environment in the UK has been largely stable. The deal regarding Brexit has been settled in late January 2020, before the start of the COVID-19 pandemic in the UK. It is reasonable to argue that the observed changes of people’s income, time use, and subjective wellbeing are correlated with the spread of COVID-19 and its related measures. We have made relevant changes in the title of this manuscript.

We agree with the reviewer that the COVID-19 induced symptoms themselves could alter people’s behavior and feelings. Accordingly, we further included people’s COVID-19 test outcomes (a variable with four categories at a person-year level: no test, tested positive, tested negative, or test result pending) in the models to consider the impact of contracting COVID-19 on income, time use, and subjective wellbeing. Please see the variable section for more details [pp. 9]. We have now provided the updated results. Now, the estimation of the period indicator has removed the effect due to the contraction of the COVID-19. The updated results are similar to what we have reported previously.

Moreover, we have further included the macro-level COVID-19 death rates in the UK in the models (including more COVID-19 indicator introduces multicollinearity issue). But we do not find out that these macro-level COVID-19 indicators are associated with these outcomes. We have tested other COVID-19 indicators at the macro-level, and the findings are the same. In addition, model fitness did not improve. The inclusion of the individual COVID test results may have mediated most of the impact of the macro-level COVID measures. Therefore, we do not report these results in the manuscript. The purpose of this paper is *not* to separate the impacts of the spread of COVID-19 from those brought about by policies that aim to contain this virus. Rather, throughout the manuscript, we have been discussing the trends in the given period and how these trends are correlated with both the spread of COVID-19 and its related measures. Please also refer to the highlighted places to note that we do not attribute all the observed changes to those lockdown policies.

More detailed comments follow:

1. The longer sample period suggests more reliable inferences.

Thanks for the positive comment.

2. The paper says on p. 8, “clustered standard errors are used to consider the sampling design of the UKHLS survey” but provides no further information, even in the Supplement. Without these details, it is difficult to assess whether the statistical significance is overstated, although the pattern of mean effects is quite convincing.

The UKHLS uses stratified sampling method. Samples are clustered in strata, which is based on postal addresses. Clustered standard errors have considered this survey design effect. We have added these details on page 10.

3. The paper controls for keyworker status to understand the greater decline of male earnings and hours worked during the first lockdown period. A parallel analysis should have been conducted in the ethnicity and education level comparisons, since BAME and less-educated people may have been more likely to among keyworkers.

We have added this additional analysis as suggested. Please see the updated figures in the Results section.

4. The paper finds that overall wellbeing was worse during lockdown periods than non-lockdown periods but does not try to distinguish between the effects of COVID-19 and the lockdown measures. Clearly COVID-19 infection and death rates and lockdown 2 policies have bidirectional causation: higher infection/death rates induce lockdown policies, which then (temporarily) reduce them. Does controlling for the monthly COVID-19 infection and death rates improve explanatory power in the models?

The purpose of this paper is not to separate the impacts of the spread of COVID-19 from those of policies that aim to contain this virus.

In a supplementary analysis, we have included the macro-level COVID-19 death rates in the UK in the models (including more COVID indicator introduces multicollinearity issue) to predict subjective wellbeing and noted that a higher level of the daily COVID-19 death rates is associated with worsened subjective wellbeing. However, the large standard error prevents us from drawing a reliable conclusion (beta= 1.122, p=0.681). The purpose of this paper is to provide an overview of multiple outcomes in the given period rather than discussing whether it is COVID-19 per se or any specific lockdown policy that are associated with the outcome. We believe that the current analysis should be sufficient to serve this purpose.

To incorporate the impact of the macro-level COVID-19 indicator on people’s subjective wellbeing, we have revised the part discussing the negative impact on the subjective wellbeing to include the impact of the negative news related with surging infection and death cases [pp.3]. We have also mentioned this in the final discussion to highlight that we do not assert that only these lockdown policies are associated with changes in the subjective wellbeing [pp.23].

This paper describes the trends in people’s income, time use, and subjective wellbeing from before this pandemic till late March 2021, during this period people in the UK have experienced this unprecedented pandemic together with many measures that aim to contain this virus. In line with most of the COVID-studies cited in this paper, we analyze outcomes at different time points over a period to improve our understanding of the impact of the pandemic on people’s lives. We believe that this analysis has offered important foundation to understand the potential impacts of this pandemic.

5. The paper should be more cautious in attributing the results to COVID-19 (and possibly seasonal affective disorder). Given that there may have been important policy changes beyond those related to COVID-19, as well as voluntary changes in behavior, all the paper reports is the net effect of all changes. For example, in the U.S., Blanchflower and Bryson (2021 NBER) examine mental health scores from April 2020 through June 2021, and find that anxiety, depression and worry peaked in November 2020, coinciding with the U.S. Presidential Election. Without searching for events that might have changed the national mood (e.g., Brexit fallout) other than COVID-19 and related policies, I think that the paper might be over-attributing changes to COVID-19.

The past year in the UK has been dominated by the COVID-19 pandemic. During this period, the political environment in the UK has been largely stable. The deal regarding Brexit has been settled in late January 2020, before the start of the COVID-19 pandemic in the UK. It is reasonable to argue that the observed changes of people’s income, time use, and subjective wellbeing are correlated with the COVID-19 and its related measures.

Blanchflower and Bryson (2021) adopted the same time-point approach with our paper. They have found a worsened subjective wellbeing during November 2020 when the US election outcome was about to announce/announced, although people might have been affected by other policy changes or social movements happening in November 2020. We cited this work on page 4 as suggested. Therefore, it is almost never possible to say that all the changes are due to a single factor in this kind of research.

We have remarked in the Discussion section that the some changes we have observed might be due to other parallel policies that are not related to the pandemic [pp. 23]. Please also refer to the highlighted sentences to note that we do not attribute all those observed changes to lockdown policies. We believe that this analysis has offered important foundation to understand the potential impacts of the pandemic.

6. The paper should be sent out for professional proof-reading before resubmission.

Thanks for the comments. This paper has gone through professional proof-reading.
